# 22 years of satellite imagery reveal a major destabilization structure at Piton de la Fournaise

**DOI:** 10.1038/s41467-022-30109-w

**Published:** 2022-05-12

**Authors:** Quentin Dumont, Valérie Cayol, Jean-Luc Froger, Aline Peltier

**Affiliations:** 1 Université Clermont Auvergne, CNRS, IRD, OPGC, Laboratoire Magmas et Volcans, F-63000 Clermont-Ferrand, France; 2 Université Jean Monnet - Faculté des Sciences et Techniques, Laboratoire de géologie de Lyon : Terre, Planètes, Environnement - UMR CNRS 5276 LGL-TPE, 42023 Saint-Etienne, France; 3 Université de Paris, Institut de Physique du Globe de Paris, CNRS, Paris, France; 4 Observatoire Volcanologique du Piton de la Fournaise, Institut de Physique du Globe de Paris, La Plaine des Cafres, France

**Keywords:** Geophysics, Natural hazards, Geology, Volcanology

## Abstract

Volcanic activity can induce flank failure, sometimes generating large earthquakes and tsunamis. However, the failure structures have never been fully characterized and the failure mechanism is still debated. Magmatic activity is a possible trigger, either through fault slip, which might be induced by dyke intrusions, or through sill intrusions, which might be undergoing coeval normal displacements and slip. At the Piton de la Fournaise volcano, satellite imagery combined with inverse modeling highlights the pathways of 57 magmatic intrusions that took place between 1998 and 2020. We show that a major arcuate dyke intrusion zone is connected at depth to a sill intrusion zone, which becomes a fault zone towards the sea, forming a spoon-shaped structure. Some sills are affected by coeval normal displacement and seaward slip. Overall, the structure is characterized by a continuum of displacement from no slip, to sheared sills and finally pure slip. Repeated intrusions into this spoon-shaped structure could trigger catastrophic collapses.

## Abstract (web summary)

At Piton de la Fournaise volcano, satellite deformation allows to constrain the geometry of 57 magmatic intrusions. Versatile modeling reveals that a major spoon-shaped destabilization structure accommodates intrusions and seaward flank displacement.

## Introduction

Flank destabilization of volcanoes is a major hazard as it can trigger tsunamis and large earthquakes, which, when combined, account for 24% of volcanic fatalities^1^. Large flank destabilizations are common on oceanic islands, as demonstrated by bathymetric studies which show ubiquitous debris avalanche deposits^2^. Evidence from geological^3,4^, geophysical observations^5–9^, and analysis using physical and analog models^10–14^ indicate that flank destabilization at these volcanoes may be triggered by magmatic activity.

This activity often occurs along preferential intrusion paths, generally referred to as “rift zones”^15^, which are, in turn, controlled by the stress field of the edifice^10,16^ and by structural weaknesses^17,18^. Rift zones can be fed by magma either sporadically or continuously, and vary in size from a kilometer to tens of kilometers long. Despite these differences, their surface expression is characterized by a high density of pyroclastic cones and eruptive fissures aligned along one or several preferential directions along which magma may be guided away from a central vertical conduit.

**Fig. 1:**
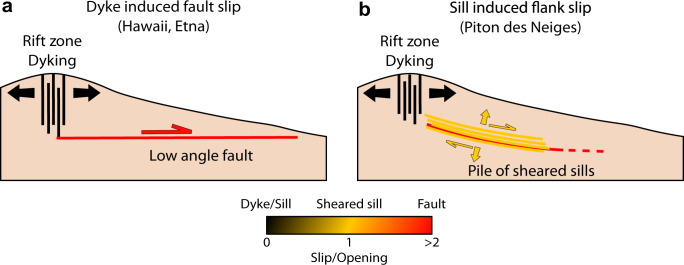
Cross-sections of conceptual models of flank destabilization triggered by magma intrusions. **a** Dyking in vertical rift zones is coupled to a low-angle fault, inducing flank movements, as found at Kilauea^5,20,54^ and Etna^6^ using geodetic data. **b** Sheared sills intruding a preexisting fault induce flank destabilization, as observed at Piton des Neiges from field studies^3,24^. Adapted from Chaput et al.^76^.

To date, two mechanisms of magma-induced flank destabilization have been proposed. The first mechanism (Fig. 1a), corresponding to subvertical intrusions (dykes) in rift zones coupled to a low angle fault^5–7,10–12,19^, was proposed for Kilauea (Hawaii, U.S.A), Mount Etna (Italy) and Cumbre Vieja (La Palma, Canary Islands) volcanoes. At Kilauea, geodetic data reveal that intrusions within vertical rift zones push the southern flank of the volcano seaward^5,7^, episodically leading to flank failure. Flank slip is accommodated by a deep décollement-type fault, located at the interface between the sea floor and the edifice, sometimes generating large earthquakes, such as the M7.2 in 1975^20^ and the M6.9 in 2018^21^. At Etna and Cumbre Vieja, the faults accommodating dyke intrusions are detachment-type faults, located at a shallow level in the edifice^6,19^. The second mechanism (Fig. 1b), corresponding to subhorizontal intrusions (sills) undergoing coeval opening and slip^3,22^, was proposed for Piton des Neiges, the extinct neighbor volcano of Piton de la Fournaise (Réunion Island). Identification of a detachment-type fault intruded by a pile of sills, separating an extinct gabbroic magma chamber from debris avalanche deposits, led to the suggestion that sill injections into the fault could induce rapid co-intrusive slip^3^, as shown by the seaward oriented magnetic fabric observed in the sills^4^. Because a previous structure acts as a guide for the intrusions, magma is not necessarily emplaced perpendicular to the minimum principal stress, in which case the intrusion may undergo coeval opening and slip^23^. If the previous structure is a fault and the fault extends beyond the intrusion zone^22^, the intrusion of magma may induce failure of the whole fault, possibly leading to flank collapse. In the longer term, heat from the cooling sills in the detachment promotes syn-deformation hydrothermal alteration which weakens the surrounding rocks, leading to low-temperature creep^3,24^.

**Fig. 2:**
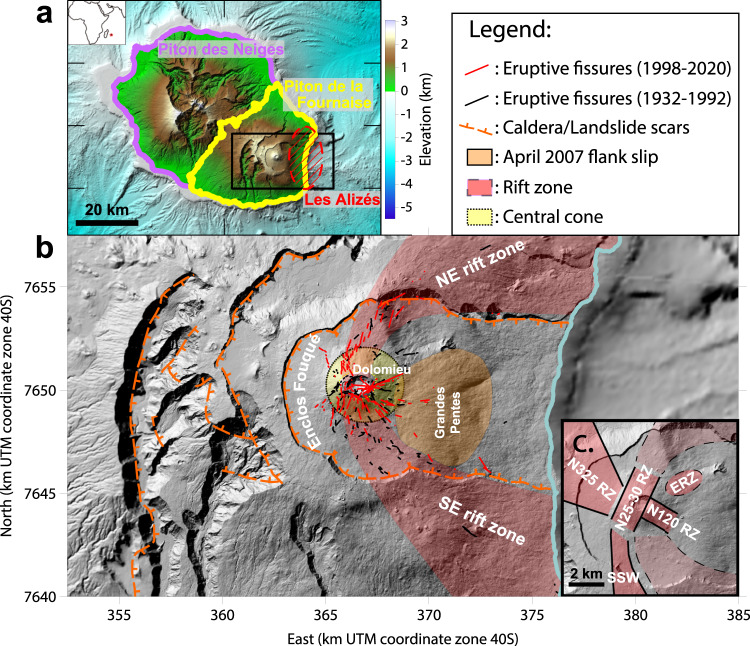
Location map. **a** Location of the three volcanic edifices on Réunion island. The black rectangle indicates the area shown in (**b**). **b** Map and structural features of Piton de la Fournaise edifice. **c** Main rift zones as outlined in previous studies^18,36–38^. Rift Zone (RZ), East Rift Zone (ERZ), South-south-west volcanic alignment (SSW).

**Fig. 3:**
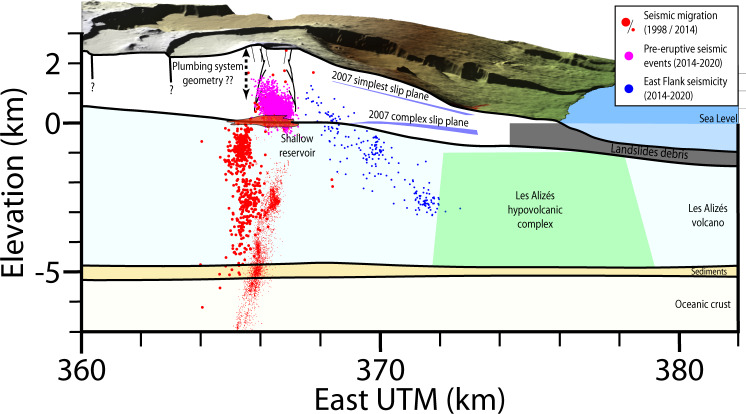
Internal structure of Piton de la Fournaise. Earthquake locations^31,39,40,77^. Blue surfaces show the 2007 post-eruptive slip surface determined for two different sets of model parameters^32^. Figure modified from Lénat et al.^78^.

At Piton de la Fournaise, collapse of the eastern flank represents one of the major hazards. Bathymetric studies have shown that debris avalanche deposits of up to 100 km^3^ are present on the submarine flanks of the volcano at distance up to 80 km from the shoreline^25^. Moreover, it has recently been demonstrated that flank slip is still ongoing: in March–May 2007, the largest eruption of the last hundred years took place on the lower eastern flank of the volcano. Synthetic aperture radar interferometry (InSAR) showed that the eruption was associated with an eastward slip of the eastern flank of the volcano of up to 1.4 m, with 0.35 m of upward motion^26^ (see Fig. 2). Since then, InSAR and global navigation satellite system (GNSS) data have shown that there is a continuous slip at a rate of 1.4 cm/yr^9,27,28^, which accelerates with magmatic activity^29–31^. The presence of a fault under the eastern flank (Grandes Pentes area in Fig. 2) was proposed to explain the co- and post-eruptive deformation recorded during the 2007 eruptive crisis^23,32,33^. Inverse modeling of post-eruptive deformation^32^ points to a fault plane sub-parallel to the topography with a depth ranging from ≈500 to ≈1500 m depending on the parameters considered in the inversion (Fig. 3). These observations raise the question of a link between the present magmatic activity and the possibility of a catastrophic flank collapse at the volcano. In addition, Piton de la Fournaise is one of the most active volcano in the world with an average of one eruption every five months and one of the best monitored with continuous displacement monitoring by both InSAR and GNSS since 1998.

Here, we take advantage of the exceptionally high activity of Piton de la Fournaise and the large amount of displacement data to image the shallow plumbing system in 3D, as well as the structures which accommodate flank slip. The geometry of 57 magmatic intrusions that occurred between 1998 and 2020 are determined relying on state-of-the-art inverse modeling in a fully 3D framework, allowing for curved intrusions and stress changes. We link the imaged intrusions to the rift zones previously recognized at the surface. A major spoon-shaped active structure, accommodating coeval opening and slip is highlighted. We compare this structure to other volcanoes and discuss the implication for the stability of the volcano.

## Results

### Piton de la Fournaise plumbing system

Piton de la Fournaise is located in the south-east of Réunion Island in the Indian Ocean (Fig. 2a). The volcano is buttressed by the older volcanic edifice of Piton des Neiges, located to the north-west. Piton de la Fournaise is built on an intrusive gabbroic complex possibly originating from an older edifice (Les Alizés), which has been inferred as lying to the east of the volcano from drill-hole^34^ and gravity studies^35^ (Figs. 2a and 3).

Located inside a U-shaped collapse structure (Enclos Fouqué), the current volcanic center has formed a 400 m-high central cone with a 1 km-wide and 300 m-deep summit crater (Dolomieu) (see Fig. 2b). Several rift zones (Fig. 2c), radiating from the central cone, have been identified from locations and orientations of superficial morpho-structural markers, such as eruptive fissures or cinder cones. The main rift zone has an overall NE-SE arcuate shape passing through the central cone^36–38^. It extends beyond the walls of the Enclos Fouqué, both to the north and south, and reaches down to the shore. Additional, less active, intrusive directions have also been identified: one, N120^∘^, running from the southeast edge of the Dolomieu crater to the base of the central cone^18,37^, an East Rift Zone striking N60^∘^^36,38^, a N300-325^∘^ direction, and a volcanic alignment toward the south-south-west^37^.

The deeper part of the plumbing system has been interpreted mainly through seismicity (Fig. 3). A sub-vertical conduit, extending between −5 km and sea level, and located 500–1000 m west of the summit was outlined by seismic event migrations during the 1998 and 2014 eruptive crises^39,40^. Its location was confirmed by P-wave velocity tomography^41^ and the modeling of the deformation recorded at the beginning of the 2014 crisis^42^. Around sea level, the presence of a main shallow reservoir is postulated from a discontinuity in the location of micro-earthquakes^39^ and the P-wave velocity structure^41^, as well as from pre-eruptive deformation^8,42^. Pre-eruptive seismic swarms also occur before eruptions between sea level and 1 km above sea level (asl)^40,43,44^. However, no event migration has been identified above 1 km asl, preventing to precisely image the shallow plumbing system. The shallower part is known through deformation. The analysis of InSAR and GNSS data associated with 16 previous eruptions^32,33,45–50^ highlights shallow discrete dykes, but no continuous structure. Our study provides the first extensive image of Piton de la Fournaise shallow plumbing system.

### Inverse modeling of intrusions

Our inverse modeling approach has two characteristic features (see “Methods”). The first is that intrusions can be curved. Indeed, curved intrusions are sometimes required to account for the strong asymmetrical displacement^50–53^, characteristic of most Piton de la Fournaise eruptions. This asymmetry is best explained by curved intrusions corresponding to sill-to-dyke transitions^48–50^. Intrusions are meshed by triangular elements, making it possible to create such complex geometries. The second feature is that stress changes are determined on fractures, as opposed to most geodetic studies which determine displacement on fractures^20,54^. Here, we chose to determine overpressure and shear stress changes (normal and tangential to the fracture elements, respectively), as these quantities are more physical and more informative than displacements. This study is the first that systematically searched for possible curvatures and allowed coeval shear and normal stress changes, when it improved data fit.

Between March 1998 and December 2020, 54 intrusions leading to eruptions and 15 failed eruptions (i.e., intrusions that did not reach the surface, but created a seismic crisis and deformation) occurred. All were captured by either InSAR or GNSS, or both (see “Methods”). Among these intrusions, we analyzed 57 events and discarded 12 events which did not produce deformation detectable by InSAR (>2 cm). Of the 57 intrusion models considered for our analysis, 16 had already been determined and 41 are brand new computations. Among the already determined models, 8—based on InSAR data—were not recomputed (March 1998, July and September 1999, February 2000 and June 2000^45,48^, March–May 2007^32,33^, May 2016^49^ and July 2017^50^), while 8 others—solely based on GNSS data—were recomputed based on InSAR data (August and September 2003^46^, May and August 2004, February, October, November and December 2005^47^). Indeed, the GNSS network is located around the summit and misses displacements associated with eruptions beyond the summit area^49^.

Of these models (see Supplementary Figs. S6–S62 for detailed model results), 8 have large uncertainties because of low signal-to-noise ratios of displacement measurements, which come from low displacement amplitudes (<5 cm). These models mainly correspond to small volume (<0.3 Mm^3^) summit intrusions in the Dolomieu crater^55^. Because of their small volume, these intrusions are not expected to provide any relevant information about the plumbing system at the scale of the edifice. The high uncertainties of the deformation data and the negligible associated volumes led us to discard them in our analysis.

**Fig. 4:**
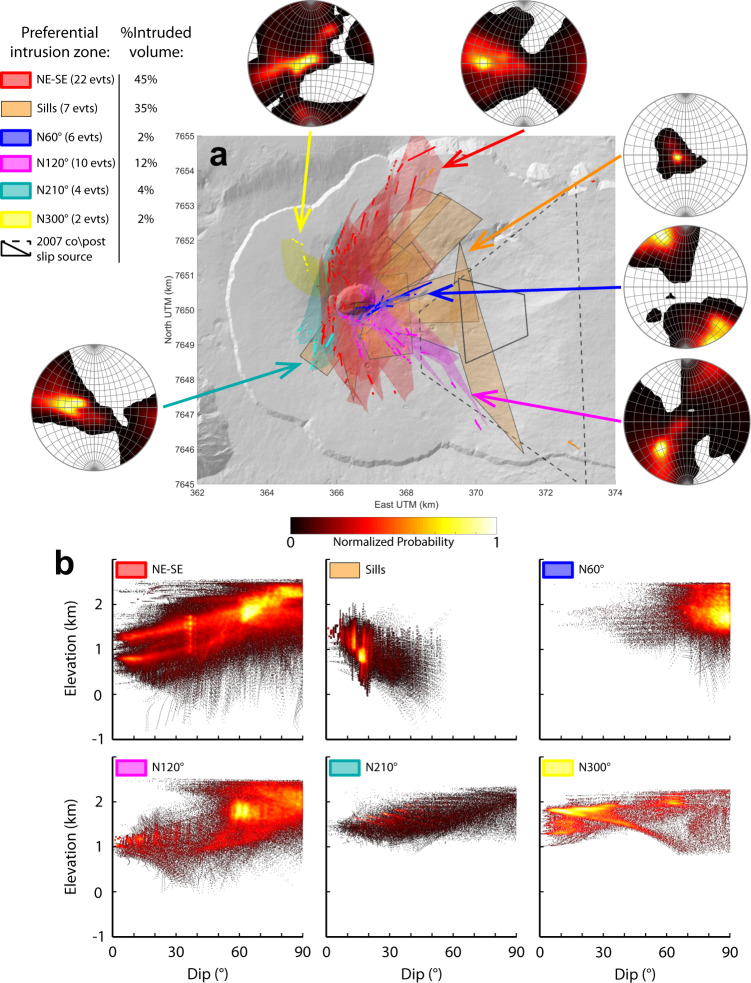
Intrusion pathways from inverse modeling. **a** Model locations are shown in six different colors based on the intrusion zone to which they belong. The number of events in each intrusion zone is indicated in the legend. Bold lines show the eruptive fissures, using the same color code as for the intrusions. Contours of the co- and post-eruptive model of March–May 2007^32,33^ are shown for comparison by dashed and solid gray lines, respectively. For each intrusion zone, the poles of triangular elements of models within their 95% confidence interval are given in a stereographic projection on the lower hemisphere (see “Methods”). **b** Variation of the elevation with the dip of triangular elements belonging to each intrusion zone.

All inverted models show a consistent spatial distribution, with swarms of intrusions close to each other, in agreement with the similar displacement patterns observed by InSAR at the volcano^49,50^. With the exception of one model (February 2007 at the summit), all models delineate six preferential intrusion zones radiating from the central cone. Five coincide with the rift zones previously identified from surface features^18,36–38^, while the sixth, corresponding to a sill intrusion zone at depth, is identified for the first time by our 3D comprehensive study (Fig. 4).

All intrusions are rooted beneath the Dolomieu crater at 0.5–1.5 km asl, consistent with the analysis of continuous tilt and GNSS data which show that magma starts migrating vertically in the conduit beneath the summit before being transmitted to the vent either vertically for summit eruptions or laterally for rift zone eruptions and sill intrusions^49,56^. Our study confirms that the Dolomieu crater is a turning point for magma.

### A major spoon-shaped intrusion zone accommodating seaward slip

The path with the highest number of intrusions (22 events, represented in red in the Fig. 4), connects at the surface to the already recognized major NE-SE rift zone shown on Fig. 2^36,38^. Since this rift zone accounts for the largest intruded volume (33.1 × 10^6^ m^3^, totaling 45% of the intruded volume), it confirms its role as the main preferential intrusion zone. The lack of eruptions outside the Enclos Fouqué since 1998 prevents to image the 3D shape of this rift zone outside the caldera wall. Intrusions in the summit area are planar and vertical, while those away from the summit to the NE and SE curve horizontally and vertically. Together, they form a spoon-shaped structure with a horizontal curvature, as shown by the range of strikes from N30^∘^ to NS on the north flank and NS to N120^∘^ on the south flank (Fig. 4a), and a vertical curvature, as shown by the transition from gently dipping to highly dipping intrusion with increasing elevation (Fig. 4b). This structure is consistent with a joint analysis of continuous GNSS and InSAR data which determined that an intrusion may start as a sill beneath the summit, propagate laterally before becoming vertical to reach the vent^49^.

**Fig. 5:**
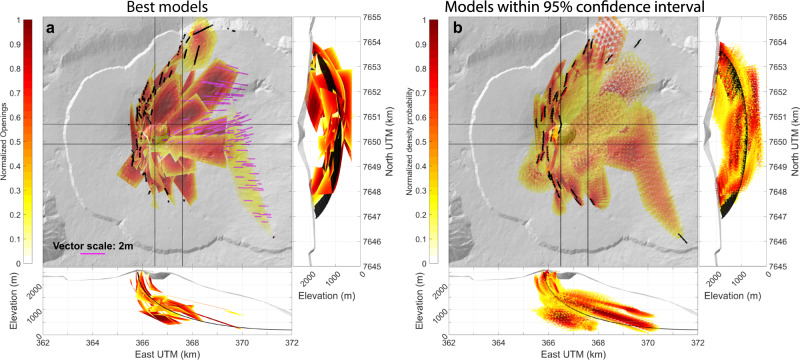
3D geometry of the main NE-SE preferential intrusion zone. **a** Best-fit models for the 29 intrusions emplaced in the major NE-SE and sill intrusion zones. The colors show the opening of the modeled intrusions (normalized for clarity of the representation). Magenta vectors indicate displacement of the sheared sills. **b** Spread of models within their 95% confidence interval. Colorscale shows the normalized density of points for each model (point densities under 0.2 are not shown for visibility). For (**a**) and (**b**), thin straight lines on the map views delineate the width of the slice shown in the adjacent cross-sections. The thin black curve shown in the cross-sections highlights the surface determined by polynomial regression of the models mesh points.

A sub-horizontal preferential intrusion zone is shown through 7 sill models beneath the eastern flank (shown in orange on Fig. 4). This intrusion zone can also be considered as major as it corresponds to the second largest intruded volume after the main rift zone (25.9 × 10^6^ m^3^), amounting to 35% of the intruded volume. All 7 intrusions have a low eastward dip (0–20^∘^), following the topography. At depth, they connect to the main NE-SE intrusion zone, extending the spoon-shaped structure to the east (Fig. 5).

Both the NE-SE and the sill intrusion zones form a single major structure, as indicated by the overlap of 95% confidence intervals of neighbor best-fit models and by the similar volumes that the NE-SE and the sill intrusion zones accommodate (45 and 35% of the intruded volume, respectively), which confirms a continuity of dilations. Together, both intrusion zones amount to 80% of the injected volume, while constituting only 50% of the number of intrusions. 3D animations highlight this structure in the Supplementary Movies S1, S2. The overall shape of the structure was estimated by quadratic polynomial regression considering the mesh points of (1) best-fit models and of (2) models randomly generated within the 95% confidence intervals. The surface determined is shown in black in Fig. 5.

**Fig. 6:**
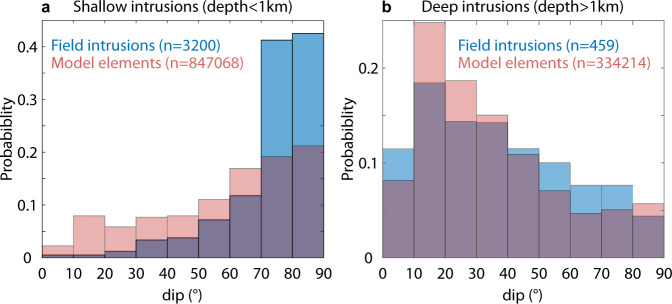
Dip distributions from field data and intrusion models in this study. **a** Shallow intrusions (depths <1 km). Data correspond to volcanoes worldwide. **b** Deep intrusions (depths >1 km). Data correspond to Piton des Neiges. Field data are taken from Cayol et al.^23^. They are compared to dips computed from triangular elements of intrusion models within their 95% confidence intervals. *n* is the number of field data or the number of triangular elements of source models.

This main spoon-shaped structure is characterized by vertical dips in the rift zone, progressively decreasing towards the east to reach ≈15^∘^ beneath the east flank, where the structure follows the topography. Dips of this structure are in agreement with field observations. Indeed, at Piton des Neiges^57^ and several other volcanoes worldwide (Koolau, Waianae, Tutulia, Tenerife, Stromboli, Piton de la Fournaise^23^), intrusion outcrops show preferential dips that correlate with emplacement depths (Fig. 6): sub-vertical intrusions are mainly found in the shallow part of edifices (<1 km), while low dip intrusions (10–40^∘^) are found at greater depths (>1 km). This distribution is close to that inferred from our models (Fig. 6), indicating that our curved models are highly plausible. Moreover, eruptions that occurred between 1998 and 2020 show vent locations and orientations similar to those of the 1932–1992 period (see Fig. 2b), which indicates that the results determined for the recent period probably also apply to the last 100 years.

A striking result is the continuously increasing slip towards the east. Indeed, the four westernmost sill intrusions show pure opening, while the three easternmost ones (January 2004 eruption, September 2020 failed eruption, and October 2019 eruption from west to east) require additional shear stress to the overpressure in order to explain the meter scale lateral displacement measured for each (see individual models in Supplementary Figs. S21, S31, S58, and S61). January 2004 and September 2020 events have shear stress changes of the same order as the overpressures. These sheared sills confirm the field observations^3,4^ of sheared intrusions accommodating flank slip. The easternmost intrusion (October 2019) is a shallow plane, predominantly undergoing slip, corresponding to a shear stress change one order of magnitude greater than the overpressure (see Supplementary Table S1). Thus, this intrusion behaves almost like a fault. East of the seven sills, the source of March–May 2007 flank displacement was only affected by shear stress, suggesting that this fracture was a fault^32,33^.

**Fig. 7:**
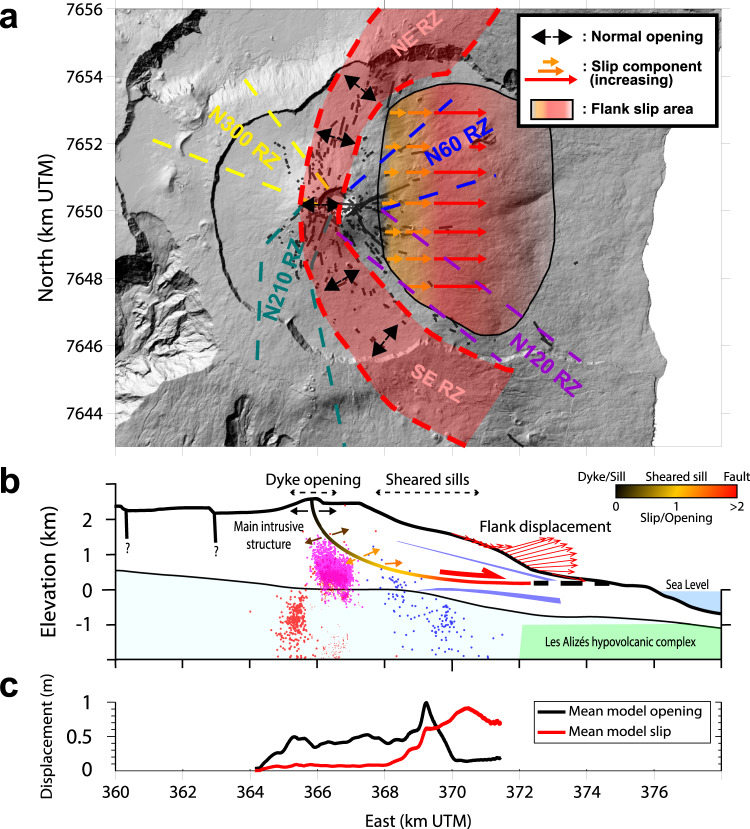
Main intrusive structure and mechanism of eastern flank displacement. **a** Map view of the identified rift zones (RZ). Flank slip associated with sheared intrusions took place in January 2004, March–May 2007, October 2019, and September 2020. **b** Cross-section showing the main intrusive destabilization structure, color-coded to reflect the amount of slip. Main geological features from Fig. 3 are indicated for reference. Earthquake locations^31,39,40,77^ are marked using the same legend as in Fig. 3. The spoon-shaped geometry is from the polynomial regression shown in Fig 5. **c** Mean opening and slip from all inverted models computed with a moving mean of 200 m radius.

Beneath the Dolomieu crater, the spoon-shaped structure follows the top of the pre-eruptive seismic swarm (Fig. 7). This swarm is consistent with a critically stressed volume beneath this structure and a mobile, less stressed, volume above. However, further east, the structure does not coincide with the seismicity of the eastern flank, which follows a 45^∘^ dip as shown by locations from the Observatoire Volcanologique du Piton de la Fournaise from the Institut de Physique du Globe de Paris (OVPF-IPGP) (in blue on Figs. 3 and 7). Therefore, this seismicity is unlikely to be directly related to slip of the eastern flank or to the propagation of intrusions. However, the temporal correlation of east flank seismicity with intrusive events in the upper part of the edifice above sea level (as in September 2020,^31^), suggests that this seismicity is triggered by stress transferred by magma intrusions.

### Secondary intrusion zones

Four secondary intrusion zones are highlighted. Two are located to the east of the summit. One, following a N60^∘^ direction, is inferred from six intrusive events (in blue on Fig. 4). This intrusion zone has been alluded to in previous work. It could correspond to the East Rift Zone^36–38^, which has a similar strike (N50-70^∘^), but is located closer to the central cone (Fig. 2). Dykes are planar and sub-vertical (see the stereographic projection in Fig. 4). They are also shallow (rooting between 1400 and 1800 m asl) and short, making the total volume of intruded magma, the smallest (1.33 × 10^6^ m^3^, 2% of the intruded volume) of all the intrusion zones. The other intrusion zone, following a N120^∘^ direction, is delineated by 10 intrusive events (Fig. 4 in magenta). It has been described previously^18,37^, but it was assumed to be limited to the summit cone. Our models show that it extends up to the Grandes Pentes. This magma pathway accommodates 12% of the intruded magma volume (8.5 × 10^6^ m^3^), which makes it the third-largest intrusion zone. Dips are mainly sub-vertical (Fig. 4), sometimes with curvature at depth. Two intrusions are planar and have 60^∘^ dips, resulting in 60^∘^ being the most frequent dip in Fig. 4. The bottom of this structure is shallow, following a mean slope of ≈10^∘^, sub-parallel to the topography (Fig. 4).

West of the summit, two secondary intrusion zones are indicated by curved eastward-dipping intrusions, which root beneath the summit before becoming vertical toward the vent. A N210^∘^ intrusion zone (Fig. 4 in cyan) is delineated by four eruptions. This path has been described previously^18^. It appears to be an extension of a volcanic alignment on the south-western flank of the volcano^37^ (SSW on Fig. 2). It is the fourth largest in terms of intruded volume (3.33 × 10^6^ m^3^), but nevertheless only corresponds to 4% of the total intruded volume. Finally, a N300^∘^ intrusion zone (Fig. 4 in yellow) is indicated by two intrusions, representing 2% of intruded volume (1.41 × 10^6^ m^3^).

It is likely that these secondary intrusion zones have been induced by the main spoon-shaped structure rather than inherited from crustal fractures. Indeed, the N120^∘^ rift zone is shallow with a lower limit following that of the NE-SE preferential intrusion zone (1000 m asl beneath the Dolomieu crater and 500 m asl beneath the Grandes Pentes). The shallow depth of this rift zone confirms, as previously suggested^58^, that it is probably induced by stress build-up in the main rift zone rather than by an inherited crustal fracture. The N60^∘^ intrusion zone also consists of shallow dykes. Its strike has the same angle from the SE branch of the main rift zone as the N120^∘^ intrusion zone from the NE branch of the main rift zone (Fig. 7), indicating that it might also result from stress build-up from the main rift zone. The N210^∘^ and the N300^∘^ intrusion zones could be related to the southern and northern extensions of the NE and SE branches of the main rift zone, respectively. Indeed, it is known that fluid-filled fractures tend to propagate along the same direction^59^. These rift zones lie in the continuation of the NE and SE branches of the main rift zone. The repeated intrusions in this rift zone might have increased the tensile stress along the main rift zone directions, despite it crossing the summit.

## Discussion

Combining InSAR and inverse modeling, we obtained an exceptionally high level of detail on a continuum of fractures, dips, and displacements (Figs. 5 and 7). The upper part of the imaged structure, which channels dykes, could result from tectonic and topographic loading, combined with buoyancy forces, as suggested for Sierra Negra volcano, Galapagos Islands^60^. However, its lower part is probably inherited. Indeed, slip is repeatedly induced along the sill part of the structure, indicating that magma is not intruded perpendicular to the least principal stress, but is instead guided by a pre-existing structure^22,23^. Moreover, the base of the structure is correlated with several known features in the edifice (Fig. 7). Its lower limit connects eastward to the base of the Piton de la Fournaise edifice^61^ (around sea level), which is known to guide magma, as indicated by a horizontal shift in seismic events recorded during the 1998 magma migration^39^. It could also correspond to ductile hyaloclastite layers^34^ or to the roof of the hypovolcanic complex of Les Alizés^34,35^, whose depths are in the same range.

The overall spoon-shaped geometry (Fig. 5) is suggestive of a rotational landslide, which could act as a destabilization surface. However, this structure differs from classical landslides, in that it accommodates magma. At the head, the structure undergoes pure opening pushing the eastern flank seaward. The associated deformation steepens the edifice slopes^52,58^ and transfers shear stress, which favors slip on the sub-horizontal surface. Further down, as the structure becomes horizontal, slip coeval with opening takes place, ultimately leading to pure fault slip at the toe of the structure (Fig. 7b, c). The easternmost part of the structure is probably locked as indicated by the abrupt transition from slip to a lack of slip towards the east.

The eastern flank steady displacement^9,28,62^ takes place in the same area as the co-intrusive flank slip (Fig. 7a). This displacement shows accelerations after intrusive events^29–31^. Thus, the slip surface is affected by rapid co-intrusive displacement, and slower steady inter-eruptive displacement. Rapid co-intrusive slip takes place during sill emplacement, such as in January 2004, or fault activation, such as in 2007. In contrast, slower steady displacement may arise from low temperature creep induced by sill emplacement which heats up the host rocks and induces fluid circulation, promoting hydrothermal alteration and weakening of the slip surface^3,24^. Creep on these surface is able to accelerate in response to co-intrusive stress perturbation. To the difference of Kilauea, where slip on a decollement (a reverse fault) is driven by continuous magma intrusions or the creep of dense cumulates in the rift zones^5,63^, the steady flank displacement takes place on a detachment (a normal fault) and thus may be driven by gravity, as assumed at Etna^6^ or Cumbre Vieja^19^. Both the steady and the co-intrusive slip release stresses within the rifts zones and probably participate to shape the rift zone as an arcuate NE-SE rift zone^13^. Thus, we propose a spoon-shaped intrusive structure characterized by a positive feedback: slip at the base of the structure is induced in response to dyke opening in NE-SE rift zone, which in turn favors the opening of new dykes in the arcuate NE-SE intrusion zone.

Because of friction, the fault to the east of the sill zone locks the system, accumulates stress, and sporadically relaxes them, either in slow slip events, as in 2007^32,33^, or suddenly, possibly triggering large earthquakes and tsunamis, as proposed at several ocean islands^2,5,10–14^.

The mechanism revealed by our study is in agreement with that inferred at Piton des Neiges where sills intrude a preexisting fault and induce slip (Fig. 1b;^3,4,22,64^). To the difference of these studies, we observe that fault slip may be induced even in absence of sill intrusion. Thus, the flank slip mechanism we propose is hybrid between models proposed for shield volcanoes: (1) dyke intrusions coupled with slip on low angle faults, whether decollement or detachments^5,6,10^ (Fig. 1a); and (2) sheared sills intruded into faults^3,22^ (Fig. 1b).

From the geometry of the structure, we estimate the average length of the failure surface of a potential collapse to be about 8.5 ± 2.5 km for a width of 10 ± 2.5 km, while the mean slope of the destabilization surface from head to toe is about 15^∘^. The estimated volume above the surface is 45 km^3^ (ranging between 14 and 96 km^3^ in the 95% confidence interval of the polynomial regression). This volume lies within the range of massive flank collapse scars observed on other oceanic islands. At Fogo (Cape Verde), the destabilization surface of the last collapse event is estimated to be ≈10 km wide and ≈12 km long for a volume of 110 km^3^ ^65^, and, at Tenerife (Canary Islands), the failure surface of the Icod landslide is estimated to be 15–20 km by 20–25 km^66^ for an estimated deposit volume of around 80 km^3^ ^67^. The structure we have imaged concerns Piton de la Fournaise, but it highlights the importance of comprehensive long-term studies of intrusive activity. At other volcanoes, similar geodetic analysis could reveal that displacements continuum—ranging from pure opening to coeval opening and slip, and to pure slip—induced by the intrusion of magma into spoon-shaped structures also control flank movements.

## Methods

### InSAR and GNSS data

The 57 intrusions with significant deformation (>2 cm) that took place between 1998 and December 2020 at Piton de la Fournaise were imaged by InSAR or by GNSS. InSAR data were acquired by the Indian Ocean InSAR Observatory (OI^2^), a component of the French National Service of Volcanological Observation (INSU/CNRS)^53^, which carries out continuous InSAR monitoring on Piton de la Fournaise using data from nine satellite missions (see Supplementary Fig. S5,^53^), while GNSS data were acquired through reiteration campaigns and by the permanent network of OVPF-IPGP. OI^2^ imaged 55 of the total of 57 eruptions and failed eruptions with at least one usable ascending or descending interferogram. For two eruptions (November 2002 and May 2003), for which InSAR data are not available, data from the GNSS campaigns were used.

### Inverse modeling

We carried out inverse modeling in order to characterize intrusions. Inverting consists of finding the intrusion that minimizes the difference between the observed and modeled displacements, in the least square sense. Model computations are based on a 3D Mixed Boundary Element Method^68^, which assumes a linearly elastic, homogeneous, and isotropic medium with a Young’s modulus of 5 GPa and a Poisson’s ratio of 0.25, following previous estimations for the volcano^45,69^. Realistic topographies are taken into account. At Piton de la Fournaise, failure to take topography into account can induce errors in the estimation of an intrusion depth and volume^45^ by 30 and 20%, respectively. Our models take fracture curvature into account, which can vary along the strike and dip directions. The along dip curvature accounts for the clear asymmetry of surface displacement often observed for magma intrusion at Piton de la Fournaise^50,53^. Previously, this asymmetry has been explained by (1) low eastward dip of dykes as in an elastic homogeneous medium^45,48,70^, or (2) complex mechanical behaviors, such as elastic heterogeneities^51^ or elasto-plasticity^52^. The complex mechanical behaviors were inferred from qualitative 2D data comparisons. In contrast, the homogeneous elastic models are 3D and inversions are used to explain the data quantitatively. Using these models, it was determined that along-dip curvature provides a better compromise between the data fit and the model simplicity than planar sources as indicated by the lower Akaike Information Criterion^48,50^. Thus, we approximate intrusion geometries by quadrangular surfaces which could be curved, with curvatures along the strike or dip directions. These fractures could be linked to the surface topography or not, depending on whether the intrusion led to the eruption of magma or resulted in a failed eruption. An intrusion geometry is defined by a set of geometrical parameters (see Supplementary Figs. S1–S3). Our models assume that fractures deform in response to constant overpressure, and constant shear stress changes, resulting in two extra parameters. The overpressure corresponds to the difference between the magma pressure and the normal stress exerted by the host rock on the fracture surface, while the shear stress change corresponds to the shear stress exerted by the host rock. We considered constant stress changes, as all parts of magma intrusions are assumed to be hydraulically connected and depth-dependent gradients can not be constrained on the scale of the intrusions at this volcano^48^. Using constant stress changes on fractures leads to smoothly varying displacements^32,68^, similarly to observations of natural fractures^71^. Our models differ from those traditionally used to invert geodetic data which determine fracture displacement, rather than stress^7,20^. Our rationale is twofold. First, the stress boundary conditions on fractures are physically more realistic than displacement boundary conditions^71^. Second, stress boundary conditions provide more information. For instance, the host rock stress may be inferred from the determined stress change^72^.

Inversions are conducted in two stages: a search and an appraisal stage. The search stage explores the parameter space by combining forward model computation and a neighborhood inversion algorithm^45,73^. The appraisal stage^74^ is based on the Bayesian inference, and uses models computed in the search stage. A mean model, one- and two-dimensional marginal probability density functions (PPD) are computed. The one-dimensional PPDs provide the confidence intervals, while the two-dimensional PPDs provide trade-offs between parameters. More detailed explanations on the iterative procedure, subsampling, and appraisal are given in the Supplementary.

### Statistical representation of the models

When inverting ground displacement, given the modeling assumptions, the result is not a unique best-fit source but a family of possible geometries and locations that equally account for the data within their uncertainties. For each inversion, we statistically define the preferential locations and the uncertainties of intrusions, based on the triangular element of fracture models. We randomly generated meshes in the 95% confidence interval of the best-fit model obtained from the appraisal stage. The family of mesh elements is used to graphically represent (1) the spatial distribution of the element centroids and their posterior probability density, which reflects the level of confidence of the presence of the intrusion at a given point in space, and (2) the dip and strike of the element normals on a stereographic projection (the poles), in a similar way to that used in structural geology. This procedure is shown in diagrams in the Supplementary Fig. S4. Pole distribution is computed using a Schmidt Distribution (which counts points within a counting circle comprising 1% of the total area of the hemisphere)^75^.

## Supplementary information

Supplementary Materials

Description of Additional Supplementary Files

Movie S1

Movie S2

## Data Availability

Interferograms used in this study are available for registered users on the CASOAR link (https://wwwobs.univ-bpclermont.fr/casoar). GNSS data used were acquired through reiteration campaigns conducted by OVPF-IPGP and are available on request.

## Code availability

Inverse models were conducted using the DefVolc software, which is available online on the OPGC website (http://opgc.fr/defvolc).
