# Supplementary material for: 22 years of satellite imagery reveal a major destabilization structure at Piton de la Fournaise
Source: Nat Commun. 2022 May 12;13:2649. doi: 10.1038/s41467-022-30109-w (PMC9098438; doi:10.1038/s41467-022-30109-w)
Supplement: Supplementary file 1 — Supplementary Materials [file 41467_2022_30109_MOESM1_ESM.pdf]

Supplementary Materials for

22 years of satellite imagery reveal a major destabilization structure at Piton de la Fournaise

Quentin Dumont[1]*, Valérie Cayol[1], Jean-Luc Froger[1,2], Aline Peltier[3,4]

[1] Université Clermont Auvergne, CNRS, IRD, OPGC, Laboratoire Magmas et Volcans,
F-63000 Clermont-Ferrand, France
[2] Université Jean Monnet - Faculté des Sciences et Techniques,
Laboratoire de géologie de Lyon : Terre, Planètes, Environnement - UMR CNRS 5276 LGL-TPE
42023 Saint-Etienne, France
[3] Université de Paris, Institut de Physique du Globe de Paris, CNRS, Paris, France
[4] Observatoire Volcanologique du Piton de la Fournaise, Institut de Physique du Globe de Paris,
La Plaine des Cafres, France

*Corresponding author. Email: quentin.dumont@uca.fr

This PDF file includes:

Inverse modeling methodology
Statistical representation of the models
InSAR data acquisition
Details of eruption models
3D geometry of the Rift Zones
Figs. S1 to S63
Tables S1

Other Supplementary Materials for this manuscript include the following:

Movies S1 to S2

Contents

Inverse modeling methodology **4**

Modeling and inversions 4

Goodness of fit estimation 6

Source type 7

Dyke: quadrangular dislocation connected to the surface 7

Deep intrusion: quadrangular dislocation non-connected to the surface 8

Deep sheared intrusion: quadrangular dislocation non-connected to the surface and submitted to shear stress 9

Statistical representation of the models **10**

InSAR data acquisition **11**

Details of eruption models **12**

March 1998 eruption, model from Fukushima et al. [1] 14

July 1999 eruption, model from Fukushima et al. [1] 15

September 1999 eruption, model from Fukushima et al. [1] 16

February 2000 eruption, model from Fukushima et al. [2] 17

June 2000 eruption, model from Fukushima et al. [1] 18

October 2000 eruption 19

March 2001 eruption 20

June 2001 eruption 21

January 2002 eruption 22

November 2002 eruption 23

May 2003 eruption 24

August 2003 eruption 25

September 2003 eruption 26

November 2003 intrusion 27

December 2003 eruption 28

January 2004 eruption 29

May 2004 eruption 30

August 2004 eruption 31

February 2005 eruption 32

October 2005 eruption 33

November 2005 eruption 34

December 2005 eruption 35

July 2006 eruption 37

August 2006 eruption 38

February 2007 eruption 39

March/April 2007 eruption, model from Tridon et al. [3] and Cayol et al. [4] 40

September 2008 eruption 41

November 2008 eruption 42

December 2008 eruption . . . 43
November 2009 eruption . . . 44
December 2009 eruption . . . 45
January 2010 eruption . . . 46
October 2010 eruption . . . 47
December 2010 eruption . . . 48
June 2014 eruption . . . 49
February 2015 eruption . . . 50
May 2015 eruption . . . 51
July 2015 eruption . . . 52
August 2015 eruption . . . 53
May 2016 eruption, model from Smittarello et al. [5] . . . 54
September 2016 eruption . . . 55
January 2017 eruption . . . 56
May 2017 intrusion . . . 57
July 2017 eruption, model from Dumont et al. [6] . . . 58
3 April 2018 eruption . . . 59
27 April 2018 eruption . . . 60
July 2018 eruption . . . 61
September 2018 eruption . . . 62
February 2019 eruption . . . 63
June 2019 eruption . . . 64
July 2019 eruption . . . 65
August 2019 eruption . . . 66
October 2019 eruption . . . 68
February 2020 eruption . . . 69
April 2020 eruption . . . 70
September 2020 intrusion . . . 71
December 2020 intrusion . . . 72

3D geometry of the Rift Zones **73**
3D geometry of N60, N120, N210 and N300 Rift Zones . . . 73

Inverse modeling methodology

Modeling and inversions

In the inverse modeling approach, observation data are used to infer the values of the parameters characterizing a system [7]. Mathematically, the relation between the observations and the model parameters is given by:

$$\boldsymbol{d} = \boldsymbol{F}(\boldsymbol{m}) + \boldsymbol{\epsilon} \tag{1}$$

where $\boldsymbol{d}$ is the vector of observed values, $\boldsymbol{F}$ is the forward modeling operator, which is a function of the input parameters $\boldsymbol{m}$, and $\boldsymbol{\epsilon}$ is the vector of measurements and modeling errors.

For ground deformation, $\boldsymbol{d}$ is the observed displacement and $\boldsymbol{F}(\boldsymbol{m})$ is a non-linear function of the source location, geometry and pressure. When inverting deformation data derived from Interferometric Synthetic Aperture Radar (InSAR), measurement errors mainly comes from atmospheric effects [8] and discrepancies in the time periods covered by the data, while the modeling errors come from inexact assumption about the source types, number of sources [9, 5], inadequate evaluation of the source interactions [10], or an inexact mechanical framework [11, 12, 13].

Inversion of geodetic data is an ill-posed problem with non-unique solution, leading to different set of parameter values explaining the data equally well. In consequence, any *a priori* constraints on the models (as the geological significance for instance) are valuable to restrict the range of possible parameter values and better determine a relevant model.

During inversions, the parameter space is explored to determine the range of parameter values that explain the data within their uncertainties by minimizing the difference between the data and the model. It is defined by the cost function:

$$\chi^2 = (\boldsymbol{u_{obs}} - \boldsymbol{u_{mod}})^T \, \boldsymbol{C_d}^{-1} \, (\boldsymbol{u_{obs}} - \boldsymbol{u_{mod}}) \tag{2}$$

where $\boldsymbol{u_{obs}}$ and $\boldsymbol{u_{mod}}$ are respectively the vector of observed and modeled displacements, and $\boldsymbol{C_d}$ is the covariance matrix of the data allowing to weight data according to their variance.

We compute the covariance matrix in order to take into account the correlated random noise of InSAR data [7]. As proposed by Fukushima et al. [2], we assumed an exponential decrease of noise correlation with distance. It is described by:

$$C_d(d) = \sigma^2 \times e^{-d/a} \tag{3}$$

where d is the distance between two points, σ^2 is the noise variance, and a is the correlation length. Variance comes from the combined effects of (1) interferogram uncertainties (estimated in underforming areas) and (2) the uncertainties of the model. Total variance is estimated through the residuals of preliminary inversions. We used a variance value of 5×10^{-4} m^2 and a correlation distance of 850 m previously estimated at Piton de la Fournaise [2, 6]. Moreover, as ascending and descending interferograms show very contrasted displacement fields at Piton de la Fournaise, it can generate unbalanced weight of the ascending/descending data in the inversion leading to possibly biased solutions [6]. We have therefore chosen to equally weight ascending and descending datasets by correcting covariance matrix from the

datasets relative magnitude as expressed by Dumont et al. [6]:

$$\boldsymbol{C_{d_i}^{correct}} = \boldsymbol{C_{d_i}} \times \left(\frac{N_d \cdot \left(\chi_0^2\right)_i}{\left(\chi_0^2\right)_{tot}} \right), \quad \text{with} \quad \chi_0^2 = \boldsymbol{u_{obs}^T C_d^{-1} u_{obs}} \tag{4}$$

where $\boldsymbol{C_{d_i}}$ is the covariance sub-matrix of the i-th dataset, N_d is the number of datasets, $\left(\chi_0^2\right)_i$ and $\left(\chi_0^2\right)_{tot}$ are the reference misfits (corresponding to a null displacement model) of the i-th dataset and of all datasets, respectively. In the Bayesian inference framework, this correction gives the same likelihood to each dataset.

The inversion procedures are conducted in 2 stages: a search and an appraisal stage. The search stage is done by combining forward model computations with a Neighborhood Algorithm [14], where the neighborhood is defined by Voronoi cells. Forward computations are conducted with a 3D Boundary element method which takes fractures, reservoirs and topographies into account. Source interactions are taken into account. Sources and topographies are meshed with triangular elements. To avoid edge effects, the topography mesh has an extension which is at least ten times larger than the intrusion [15]. Following previous estimations for Piton de la Fournaise [16, 2], a Young's modulus of 5 GPa and a Poisson's coefficient of 0.25 were used. These values are consistent with in-situ measurements for basaltic volcanoes [17]. For each forward model, the displacements are computed for a unit over-pressure of 1 MPa which is then scaled to fit the data magnitude (see details in Tridon et al. [3]). If needed to improve the data fit, a shear stress component is added and scaled jointly with the over-pressure. Here the geometry used to represent sheet intrusion is approximated by a quadrangular dislocations defined by a set of parameters (see source descriptions in the next sections). We assumed the *a priori* probability density function to be uniform for each parameter.

The inversion algorithm starts by randomly picking $N1$ points in the parameter space, where $N1$ is an exponential function of the number of parameters, k, $N1 = 1.88 \times e^{0.65 \times k}$ [3], which is determined by the number of natural neighbors [18]. For subsequent iteration, following Fukushima et al. [2], 50 points are drawn in the neighborhood of the 50 lowest misfit points. This enables the search to be explorative enough, while keeping the inversion time reasonable. Iterations stop when one of the following conditions is reached: (1) the standard deviation on χ^2 of the 50th last model is smaller than 0.05, (2) the scaled model parameter values standard deviation of the 50th last model is smaller than 0.05, (3) the maximum number of iterations have been reached (depending on the number of parameters considered).

In order to obtain numerically computable misfits, the interferograms are subsampled with a circular subsampling or a quadtree algorithm [19], to give a large number of points in high deforming areas, and a small number of points in low deforming areas.

In the appraisal stage of the inversions, 1D and 2D posterior probability functions are estimated using the Bayesian inference [20]. The model population calculated during the searching stage is resampled by a Monte Carlo integration, allowing posterior probability density functions to be reconstructed. The confidence interval and the mean model can then be derived from the 1D posterior probability density functions.

Goodness of fit estimation

We estimate the best model fit in a more comprehensive manner than misfit by defining the percentage of explained data:

$$\%ED = \left(1 - \sqrt{\frac{\left(\boldsymbol{u_{obs}} - \boldsymbol{u_{mod}}\right)^T \left(\boldsymbol{u_{obs}} - \boldsymbol{u_{mod}}\right)}{\boldsymbol{u_{obs}}^T \boldsymbol{u_{obs}}}}\right) \times 100 \tag{5}$$

This estimator indicates how well the model fits the data relative to the data magnitude expressed by a null reference model ($\boldsymbol{u_{obs}}^T \boldsymbol{u_{obs}}$). It allows cross comparison of results between inversions having different number of datasets, data points or weighting. In addition, we compute the complementary RMS estimator giving an absolute mean value of the residuals of the models allowing to compare the residuals relatively to the measurement uncertainties.

$$RMS = \sqrt{\frac{\left(\boldsymbol{u_{obs}} - \boldsymbol{u_{mod}}\right)^T \left(\boldsymbol{u_{obs}} - \boldsymbol{u_{mod}}\right)}{N}} \tag{6}$$

where N is the number of data points.

Source type

Three different type of sources were used in this study. For intrusions that reached the surface with the fissure locations and orientations well defined, we used a quadrangular dislocation link to the surface by a given number of en echelon fractures set manually (Dyke source). For intrusions that failed to reach the surface or when fissures can not be defined, we used a quadrangular dislocation not connected to the ground surface (Deep Intrusion source). Finally, for intrusions that involves non-negligible shear component, we simplify the intrusion geometry by the same quadrangular dislocation as Deep Intrusion source but with an additional shear stress component which is linearly inverted as for the pressure value (Deep Sheared Intrusion source).

Dyke: quadrangular dislocation connected to the surface

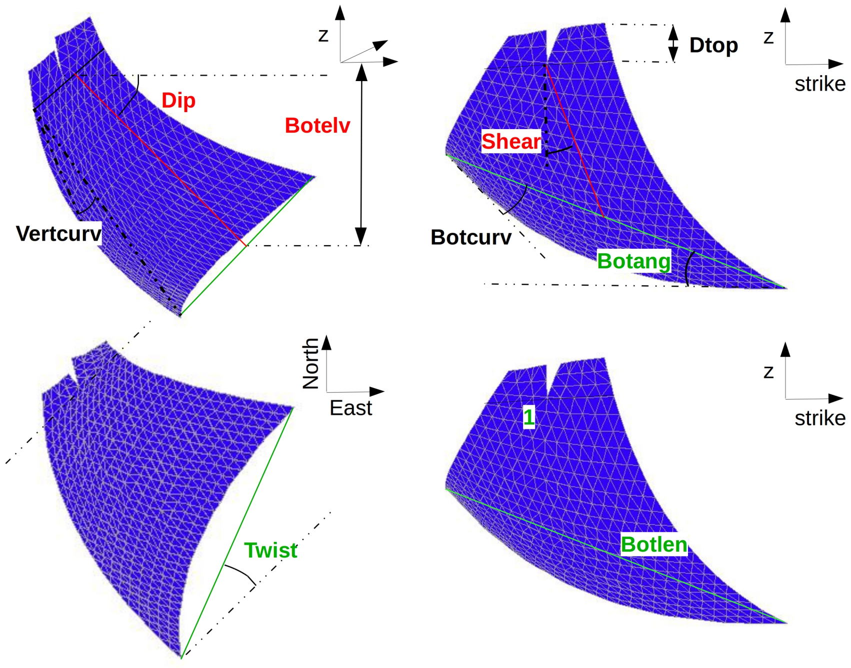

Fig. S1: Geometric parameters used to define dyke geometries. *Dip* is the dip angle; *Shear* is the angle between the dip direction and the middle line connecting the top and the bottom; *Botelv* is the elevation of the bottom midpoint; *Botlen* is the length ratio between top and bottom; *Twist* and *Botang* are the angle between the top and bottom line and the vertical angle of the bottom line, respectively; *Dtop* is the depth of the top line; *Botcurv* and *Vertcurv* are the angles defining the curvature of the bottom line and the along dip line, respectively. The surface fissures echelon and the top line are manually defined.

Deep intrusion: quadrangular dislocation non-connected to the surface

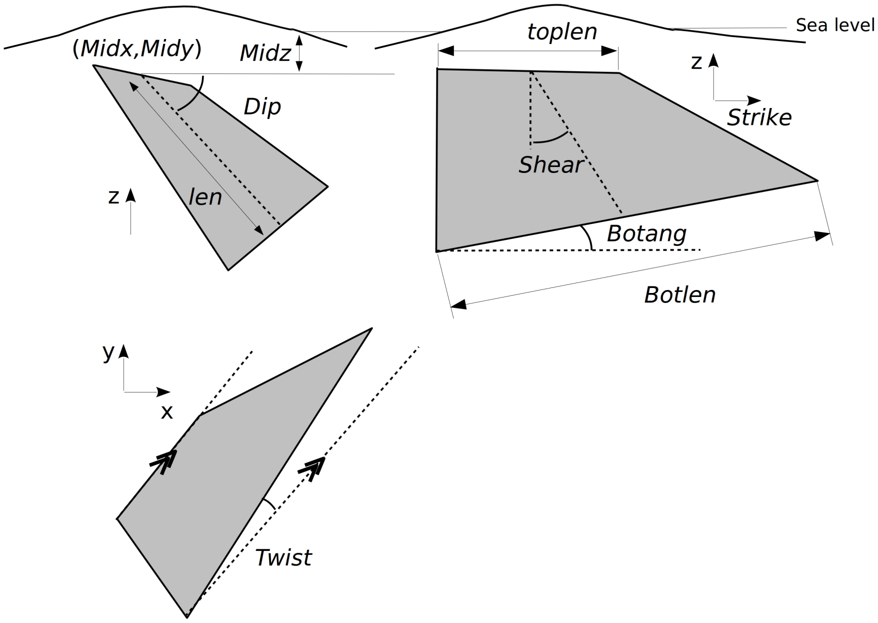

Fig. S2: Geometric parameters used to define deep intrusion quadrangle geometries. $Midx$, $Midy$, and $Midz$ are the coordinates of the midpoint of the top edge, $Strike$ is the angle to north, $Toplen$ and $Length$ are the lengths of the top edge and the length of the intrusion, respectively, and $Topang$ is the vertical angle of the top edge. Dip, $Shear$, $Botlen$, $Twist$, $Botang$, $Botcurv$ and $Vertcurv$ parameters are similar to those of dykes.

Deep sheared intrusion: quadrangular dislocation non-connected to the surface and submitted to shear stress

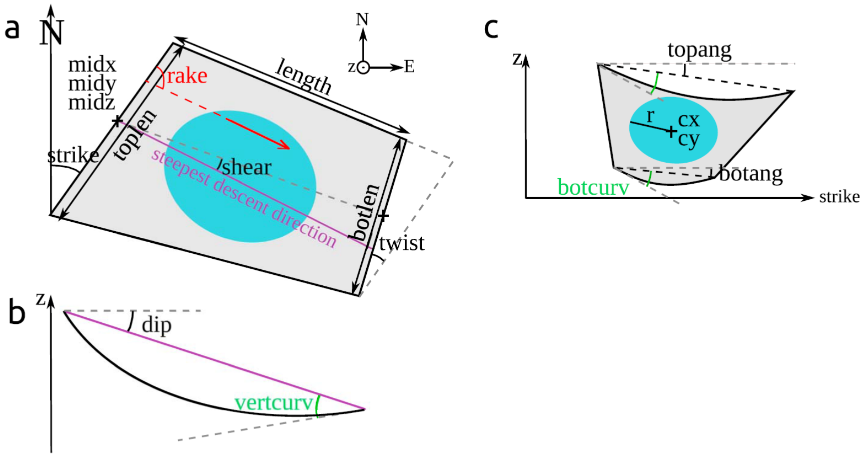

Fig. S3: Geometric parameters used to define deep intrusion quadrangle geometries subjected to pressure and shear stress changes on a circular patch. Geometrical parameters are the same as deep intrusion quadrangle except the addition of the parameters Cx, Cy, r and $rake$ which represent the x and y coordinates of the center of the circular patch, its radius and the rake of the shearing, respectively. Note that for all models done in this study, Cx, Cy, r are fixed to pressurized the whole the quadrangle. For more detail please refer to Tridon et al. [3] from which the figure is taken.

Statistical representation of the models

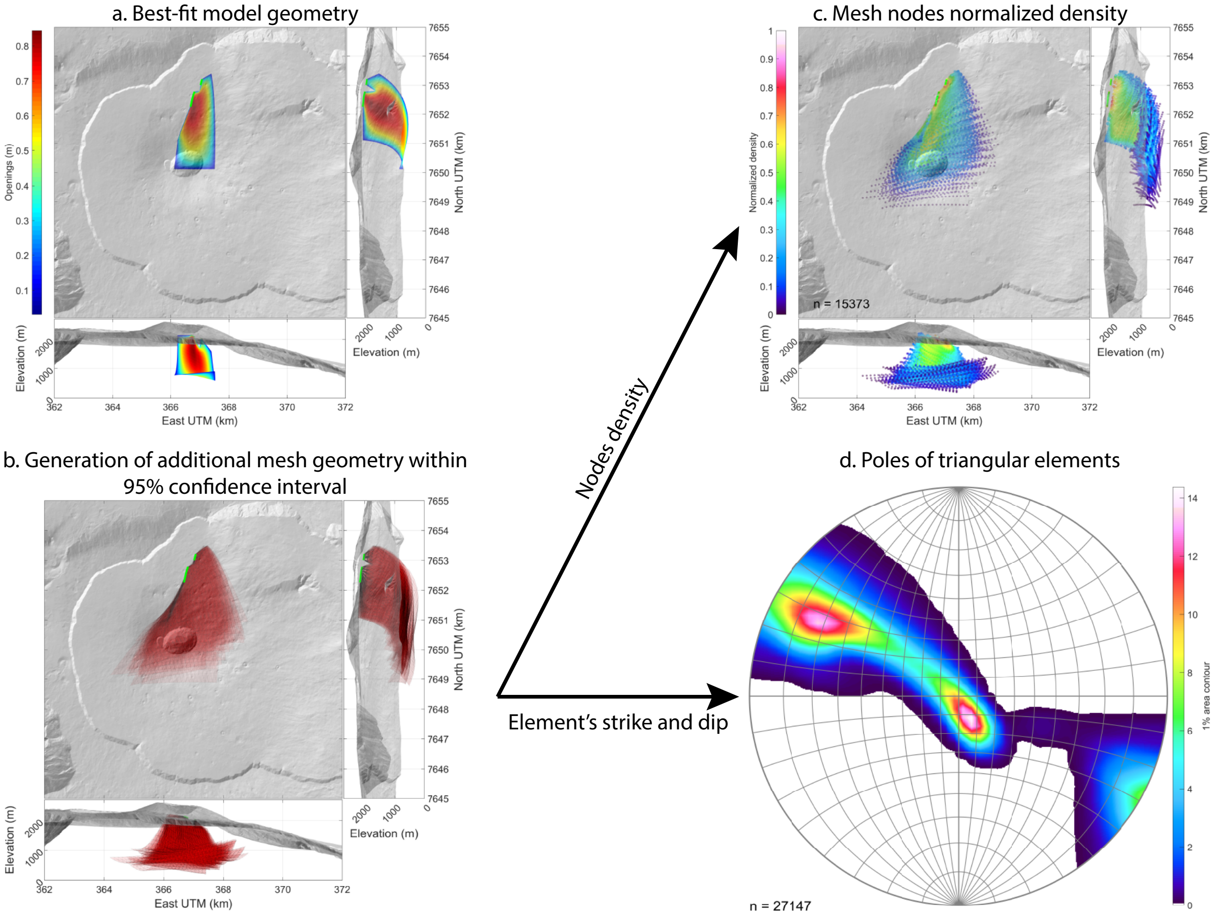

Fig. S4: Statistical representation of the models for the example of the July 2015 eruption. (a.) Best-fit model mesh color-coded by opening values. (b.) Surfaces of 50 intrusion models generated randomly within the 95% confidence interval. (c.) Centroid mesh nodes color-coded by the spatial density of nodes (n is the number of nodes). (d.) Poles of the mesh elements in a stereographic projection on the lower hemisphere projection. Color code indicates the 1% area contour of the number of poles (n is the number of poles).

InSAR data acquisition

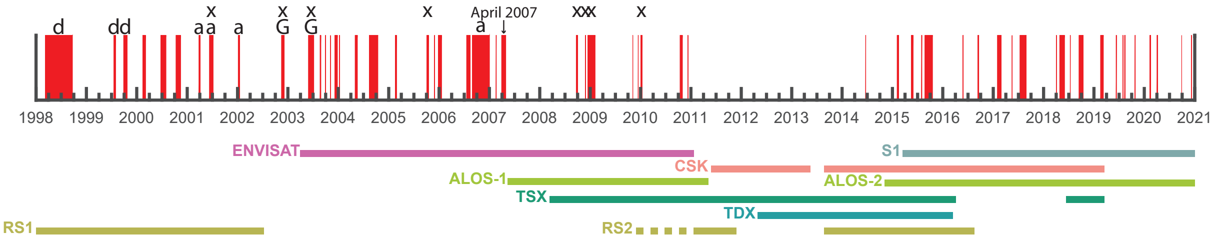

Fig. S5: Intrusive events since 1998 and available InSAR data. Each red patch indicates an intrusive event, with a width corresponding its duration. Events noted ”d” or ”a” indicate that only a descending or an ascending InSAR data is available, respectively, while ”G” indicate that only GNSS data are available. The ”x” notation indicates that a model has been determined for the eruption but not interpreted due to a too low signal to noise ratio. SAR data availability is given for each satellite: ENVISAT-ASAR (ENVISAT), Sentinel-1 (S1), RADARSAT-1 (RS1), RADARSAT-2 (RS2), COSMO-SkyMed (CSK), TerraSAR-X (TSX), TandDEM-X (TDX), ALOS-PALSAR-1 (ALOS1), ALOS-PALSAR-2 (ALOS2).

Details of eruption models

Table S1: List of Piton de la Fournaise's eruptions and model's goodness of fit. Rift zone intruded at each event is indicated based on models. Volume (Vol), overpressure (P), shear stress (S), mean opening ($\overline{Op}$) and shear ($\overline{Sh}$) values of the intrusion are given, note that shear component is typically weak except for 4 events showing more than one meter of slip. Percentage of explained data (%ED, eq. 5) and RMS in meter (eq. 6) are given for ascending data (Asc), descending data (Dsc) and all data (tot). Note that for 2002/11 and 2003/05 (*) eruptions, only GNSS data were used. Previously published models: [1] [2], [2] [1], [3] [3], [4] [5], [5] [6].

**Eruption Date**	**Rift zone**	**Vol (Mm^3)**	**P (MPa)**	**S (MPa)**	**$\overline{Op}$ (m)**	**$\overline{Sh}$ (m)**	**%ED**			**RMS (m)**		
							Asc	*Dsc*	*tot*	*Asc*	*Dsc*	*tot*
09/03/1998[2]	Main NE + sill + other	9.86	5.36/1.84/4.26	0/0/0	1.18	0.05		36	36		0.021	0.021
19/07/1999[2]	N120 (summit)	0.20	6.05/1.37	0/0	0.28	0.03		81	81		0.011	0.011
28/09/1999[2]	Main SE	0.19	3.75	0	0.71	0.20		70	70		0.010	0.010
13/02/2000[1]	Main NE	0.60	1.30	0	0.33	0.04	32	71	62	0.027	0.028	0.028
23/06/2000[2]	N120 (distal)	1.74	0.70	0	0.35	0.10	23	76	50	0.041	0.025	0.037
12/10/2000	N120 (proximal)	0.52	1.33	0	0.30	0.03	58	76	71	0.019	0.021	0.020
27/03/2001	N120 (proximal)	0.48	1.04	0	0.26	0.03	42		42	0.019		0.019
11/06/2001	other	0.60	0.53/4.20	0/0	0.17	0.03	40		40	0.018		0.018
05/01/2002	Main NE	2.46	0.95	0	0.38	0.05	57		57	0.027		0.027
16/11/2002*	other	1.46	6.67	0	1.00	0.10			41			0.059
30/05/2003*	other	0.18	10.00	0	0.89	0.00			35			0.024
22/08/2003	Main NE	0.96	0.92	0	0.30	0.05	7	85	79	0.030	0.022	0.025
30/09/2003	N210	0.90	1.26	0	0.36	0.06	29	77	67	0.026	0.023	0.025
06/11/2003	sill	1.85	1.20	0	0.48	0.03	81	71	78	0.025	0.024	0.024
07/12/2003	N60	0.21	3.53	0	0.38	0.01	20	63	32	0.034	0.012	0.025
08/01/2004	sill	7.32	2.50	3.03	1.05	1.11	42	84	76	0.057	0.048	0.053
02/05/2004	Main SE	1.07	0.75	0	0.27	0.08	22	81	71	0.034	0.029	0.032
12/08/2004	N60	0.29	0.64	0	0.20	0.04	-29	74	44	0.023	0.011	0.018
17/02/2005	Main NE	2.94	0.98	0	0.44	0.12	49	80	72	0.040	0.034	0.037
04/10/2005	Summit	0.15	14.39	0	0.85	0.03	36	36	36	0.013	0.016	0.015
29/11/2005	Main NE	0.41	3.06	0	0.44	0.05	18	71	62	0.020	0.021	0.020
26/12/2005	Main NE	4.71	2.39/0.52	0/1.40	0.78	0.14	64	43	63	0.033	0.037	0.036
20/07/2006	Main SE	0.66	1.10	0	0.27	0.05	41	62	59	0.015	0.029	0.023
30/08/2006	N120 (proximal)	0.07	2.09	0	0.20	0.02	35		35	0.012		0.012
18/02/2007	other	0.77	1.43	0	0.38	0.02	71	64	68	0.013	0.014	0.013
30/03/2007[3]	Fault ?	0.00	-0.07	3.16	0.12	1.39	26	28	28	0.107	0.126	0.011
21/09/2008	Summit	0.30	0.87	0	0.19	0.00	46	23	29	0.004	0.010	0.008
27/11/2008	Summit	0.09	19.07	0	0.88	0.02	33	25	26	0.007	0.018	0.013
14/12/2008	Summit	0.06	0.45	0	0.09	0.01	19	35	26	0.009	0.006	0.008
05/11/2009	N60	0.45	0.94	0	0.29	0.02	38	62	54	0.012	0.013	0.013
14/12/2009	N120 (summit)	0.29	1.01	0	0.23	0.04	61	36	51	0.017	0.021	0.019
02/01/2010	Summit	0.12	5.60	0	0.50	0.01	3	37	25	0.018	0.017	0.018
14/10/2010	Main SE	1.34	1.92	0	0.47	0.13	51	80	74	0.029	0.030	0.030
09/12/2010	Main NE	0.69	1.20	0	0.29	0.10	49	80	74	0.027	0.028	0.027
20/06/2014	N120 (summit)	0.46	1.64	0	0.30	0.07	18	62	36	0.025	0.011	0.020
04/02/2015	Main	0.49	0.95	0	0.30	0.04	53	62	60	0.029	0.046	0.038
17/05/2015	Main SE + N120 (proximal)	1.46	1.23	0	0.33	0.11	-3	67	56	0.028	0.030	0.029
31/07/2015	Main NE	2.82	1.13	0	0.51	0.15	65	76	74	0.021	0.034	0.029
24/08/2015	Main	0.83	3.15	0	0.51	0.07	39	72	63	0.034	0.033	0.034
26/05/2016[4]	Main SE	2.53	1.50	0	0.51	0.12	41	79	66	0.047	0.033	0.042
11/09/2016	Main NE	1.05	1.44	0	0.43	0.09	66	80	75	0.013	0.012	0.013
31/01/2017	Main SE	2.11	0.96	0	0.45	0.10	56	80	75	0.024	0.025	0.025
17/05/2017	Main NE	1.65	1.40	0	0.45	0.07	30	72	61	0.042	0.036	0.039
13/07/2017[5]	Main SE	0.75	1.34	0	0.32	0.08	54	77	74	0.018	0.028	0.024
03/04/2018	Main NE	1.92	7.18	0	1.06	0.10	38	69	62	0.041	0.049	0.045
27/04/2018	N210	0.34	1.47	0	0.27	0.06	34	71	66	0.016	0.022	0.019
12/07/2018	N300	0.79	6.37	0	0.77	0.11	38	74	65	0.025	0.023	0.024
15/09/2018	N210	1.09	4.80	0	0.75	0.14	20	71	52	0.040	0.024	0.033
18/02/2019	N60	0.66	1.29/1.35	0/0	0.35	0.03	60	72	68	0.017	0.018	0.017
11/06/2019	N60	0.27	0.77	0	0.25	0.03	6	62	50	0.021	0.021	0.021
29/07/2019	N300	0.62	0.85	0	0.21	0.05	53	70	59	0.034	0.017	0.027
11/08/2019	sill + N120 (distal)	3.99	8.81/4.87/7.23	0/0/0	0.90	0.04		73	73		0.041	0.041
25/10/2019	sill	2.00	0.92/0.11	0/1.72	0.20	0.50	28	77	64	0.070	0.051	0.061
10/02/2020	sill + N60	1.09	0.83/3.54/2.08	0/0/0	0.37	0.01	60	67	65	0.025	0.031	0.028
02/04/2020	N60	0.12	3.58	0	0.35	0.02	7	47	32	0.016	0.013	0.015
28/09/2020	sill	5.20	3.86	3.30	1.24	1.19	25	74	63	0.095	0.083	0.089
07/12/2020	N210	1.00	1.82	0	0.43	0.03	54	71	69	0.016	0.030	0.024

March 1998 eruption, model from Fukushima et al. [1]

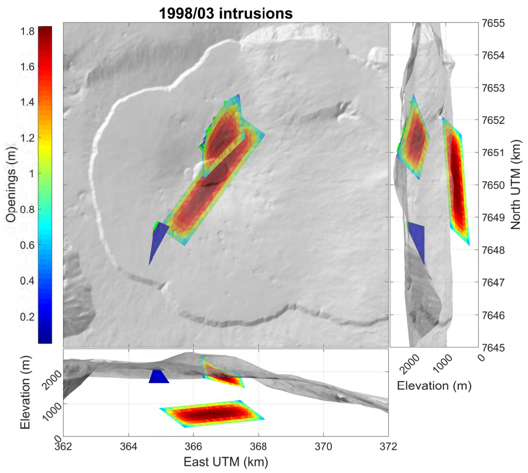

(a) 3D best geometry. Eruptive fissures are indicated by green lines.

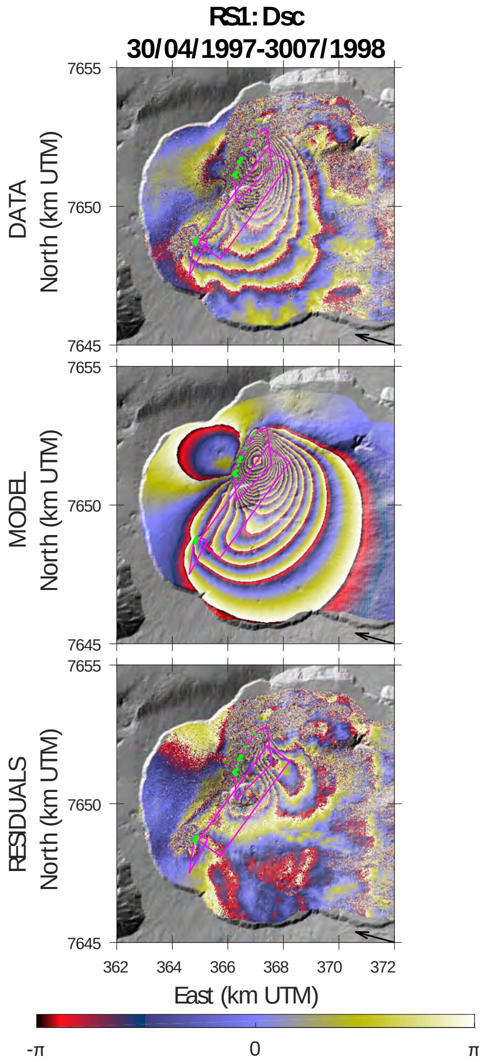

(b) Observed, modeled and residual displacements on wrapped data. Best model contour is in magenta and eruptive fissures are indicated by green lines. Line of sight of satellites acquisition is indicated by arrows.

Fig. S6: Model for the 1998 March intrusion

July 1999 eruption, model from Fukushima et al. [1]

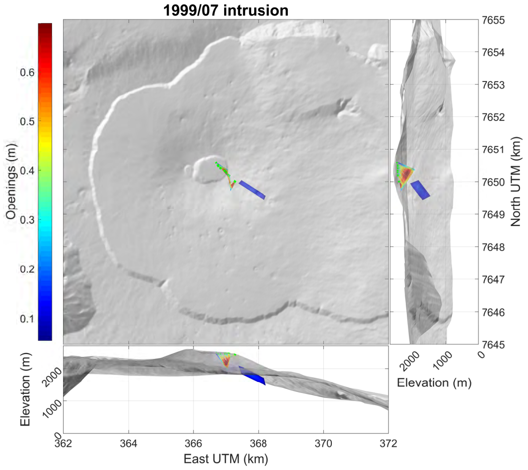

(**a**) 3D best geometry. Eruptive fissures are indicated by green lines.

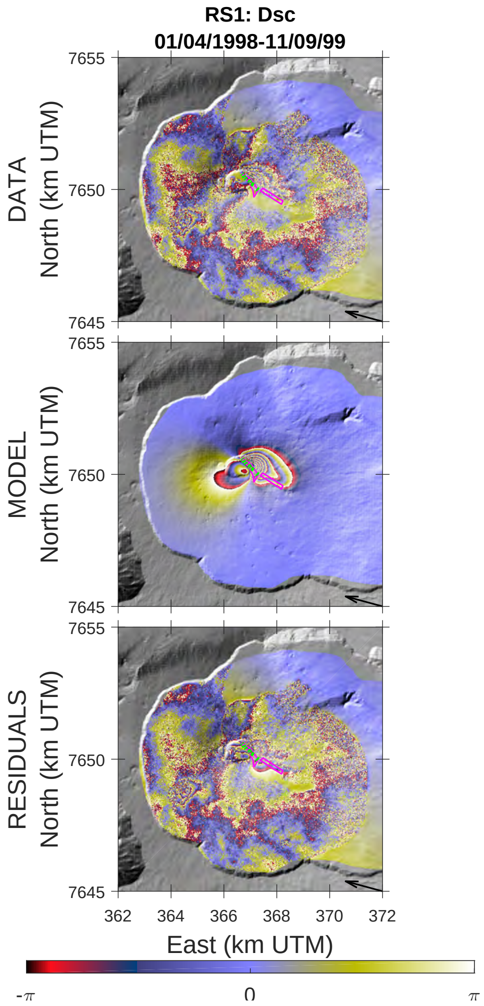

(**b**) Observed, modeled and residual displacements on wrapped data. Best model contour is in magenta and eruptive fissures are indicated by green lines. Line of sight of satellites acquisition is indicated by arrows.

Fig. S7: Model for the 1999 July intrusion

September 1999 eruption, model from Fukushima et al. [1]

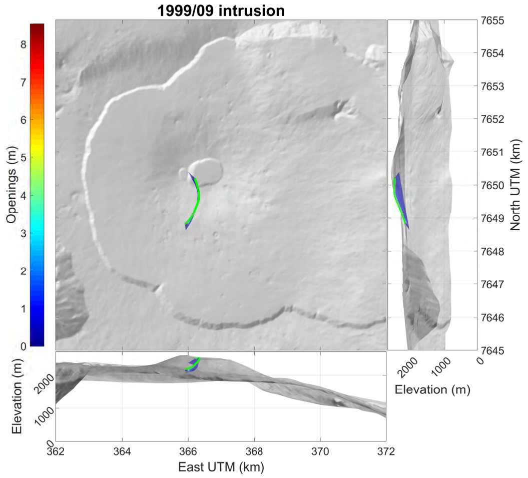

(a) 3D best geometry. Eruptive fissures are indicated by green lines.

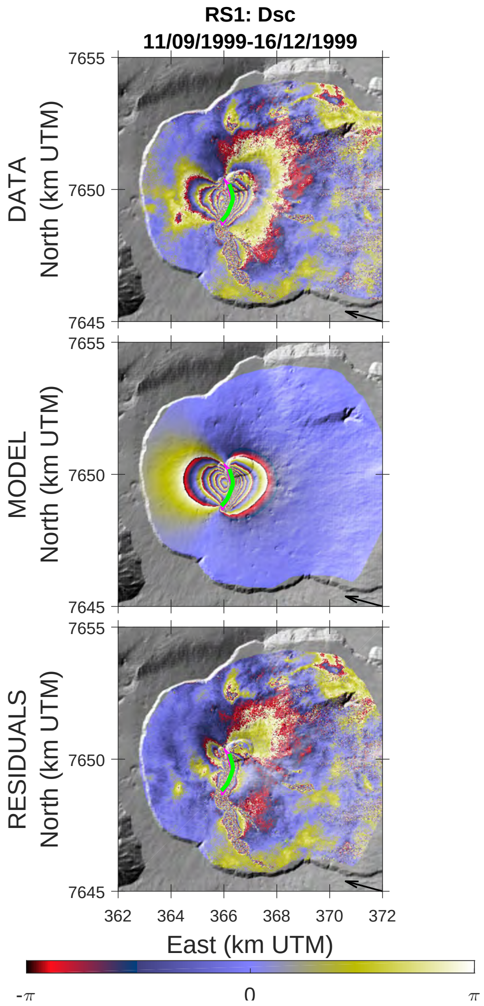

(b) Observed, modeled and residual displacements on wrapped data. Best model contour is in magenta and eruptive fissures are indicated by green lines. Line of sight of satellites acquisition is indicated by arrows.

Fig. S8: Model for the 1999 September intrusion

February 2000 eruption, model from Fukushima et al. [2]

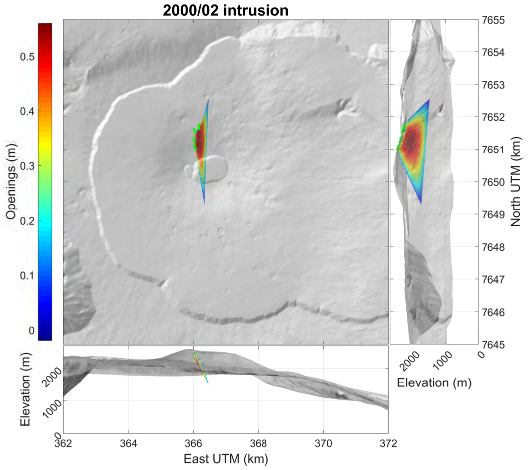

(a) 3D best geometry. Eruptive fissures are indicated by green lines.

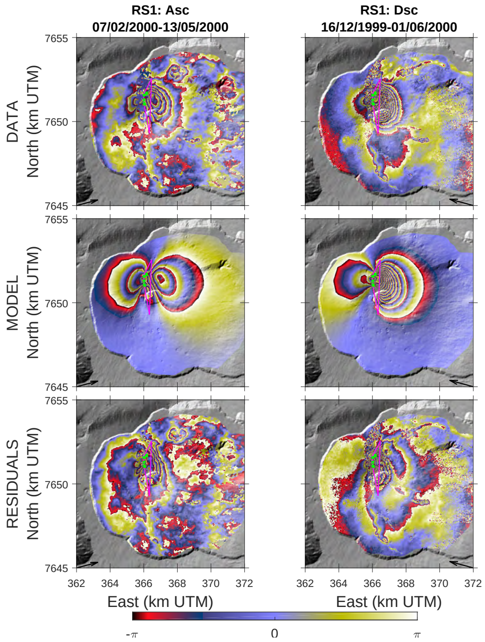

(b) Observed, modeled and residual displacements on wrapped data. Best model contour is in magenta and eruptive fissures are indicated by green lines. Line of sight of satellites acquisition is indicated by arrows.

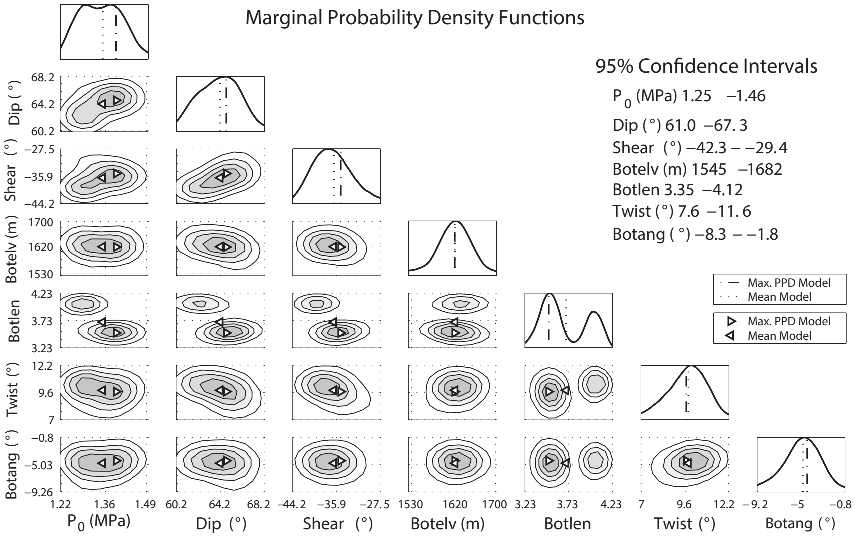

(c) Marginal posterior probability density functions. One-dimensional and two-dimensional functions are given in the diagonal and off-diagonal, respectively. Maximum and mean values are indicated by blue and red triangles, respectively. Black thick lines on one-dimensional functions represent the 95% confidence interval. Figure from [2].

Fig. S9: Model for the 2000 February intrusion

June 2000 eruption, model from Fukushima et al. [1]

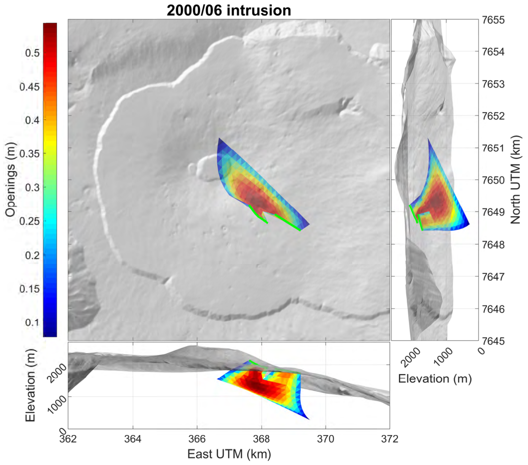

(a) 3D best geometry. Eruptive fissures are indicated by green lines.

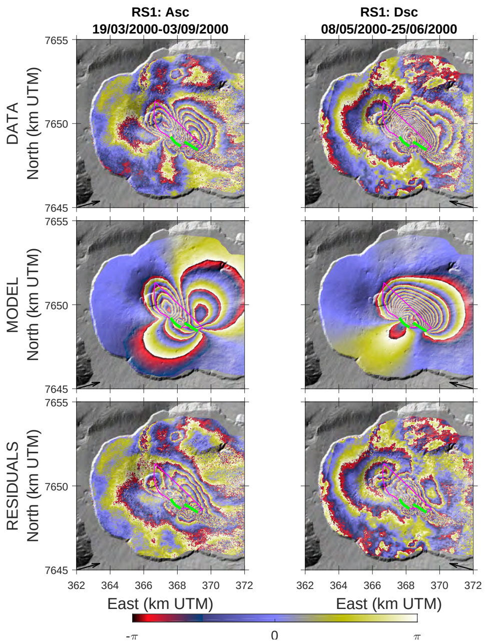

(b) Observed, modeled and residual displacements on wrapped data. Best model contour is in magenta and eruptive fissures are indicated by green lines. Line of sight of satellites acquisition is indicated by arrows.

Fig. S10: Model for the 2000 June intrusion

October 2000 eruption

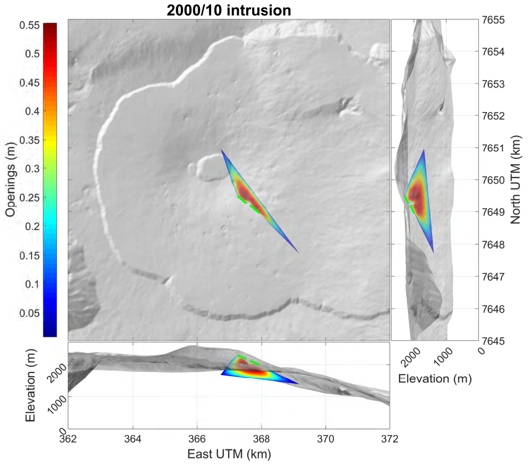

(a) 3D best geometry. Eruptive fissures are indicated by green lines.

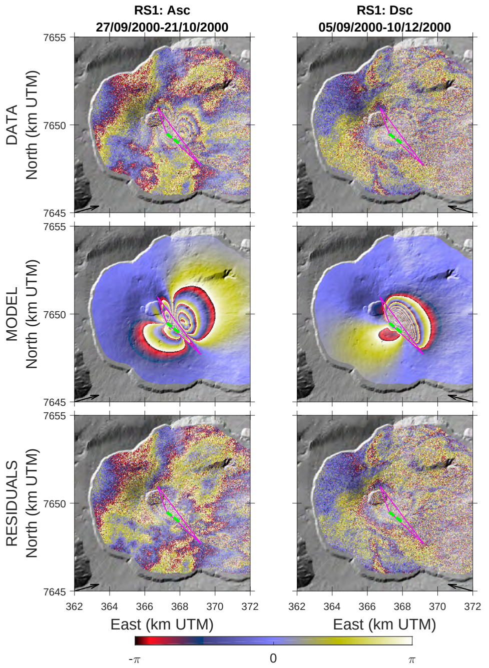

(b) Observed, modeled and residual displacements on wrapped data. Best model contour is in magenta and eruptive fissures are indicated by green lines. Line of sight of satellites acquisition is indicated by arrows.

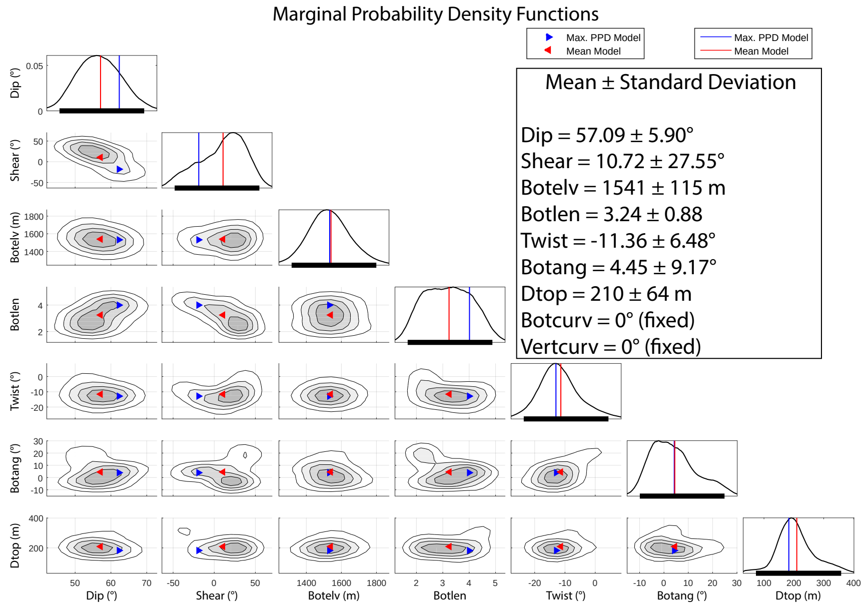

(c) Marginal posterior probability density functions. One-dimensional and two-dimensional functions are given in the diagonal and off-diagonal, respectively. Maximum and mean values are indicated by blue and red triangles, respectively. Black thick lines on one-dimensional functions represent the 95% confidence interval.

Fig. S11: Model for the 2000 October intrusion

March 2001 eruption

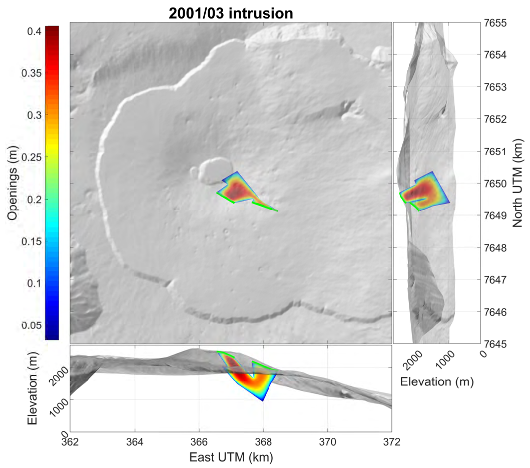

(a) 3D best geometry. Eruptive fissures are indicated by green lines.

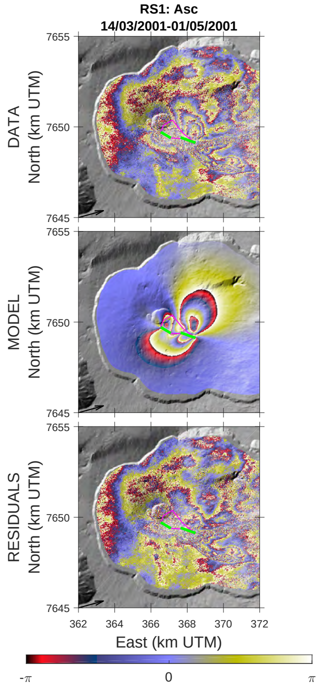

(b) Observed, modeled and residual displacements on wrapped data. Best model contour is in magenta and eruptive fissures are indicated by green lines. Line of sight of satellites acquisition is indicated by arrows.

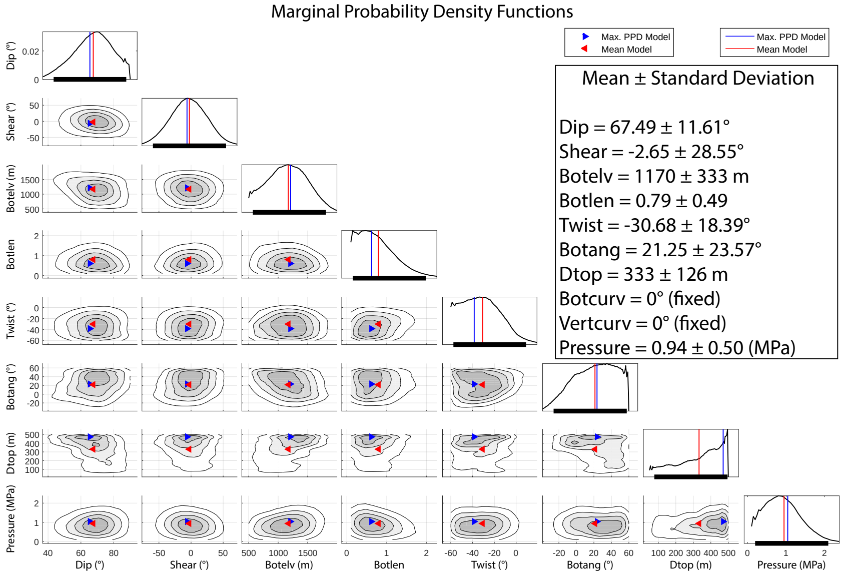

(c) Marginal posterior probability density functions. One-dimensional and two-dimensional functions are given in the diagonal and off-diagonal, respectively. Maximum and mean values are indicated by blue and red triangles, respectively. Black thick lines on one-dimensional functions represent the 95% confidence interval.

Fig. S12: Model for the 2001 March intrusion

June 2001 eruption

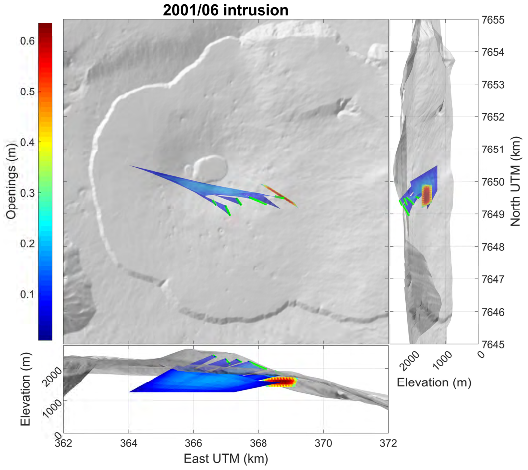

(**a**) 3D best geometry. Eruptive fissures are indicated by green lines.

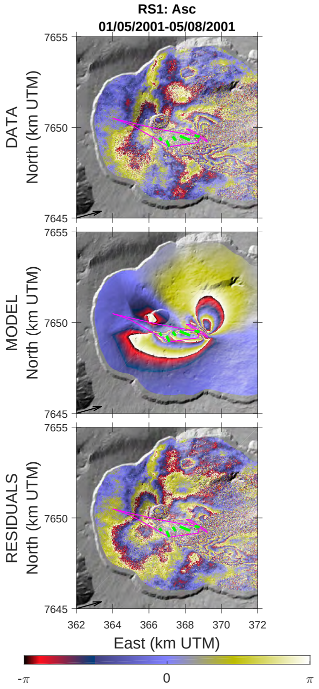

(**b**) Observed, modeled and residual displacements on wrapped data. Best model contour is in magenta and eruptive fissures are indicated by green lines. Line of sight of satellites acquisition is indicated by arrows.

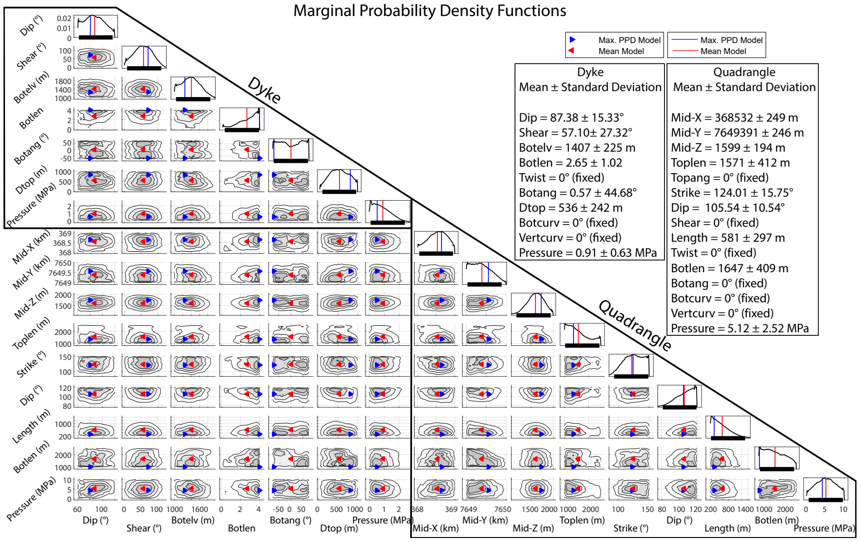

(**c**) Marginal posterior probability density functions. One-dimensional and two-dimensional functions are given in the diagonal and off-diagonal, respectively. Maximum and mean values are indicated by blue and red triangles, respectively. Black thick lines on one-dimensional functions represent the 95% confidence interval.

Fig. S13: Model for the 2001 June intrusion

January 2002 eruption

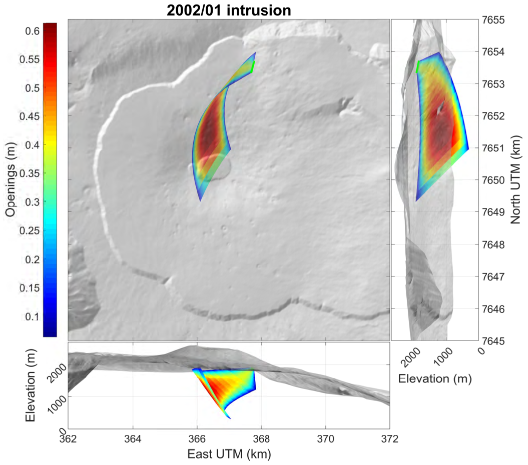

(**a**) 3D best geometry. Eruptive fissures are indicated by green lines.

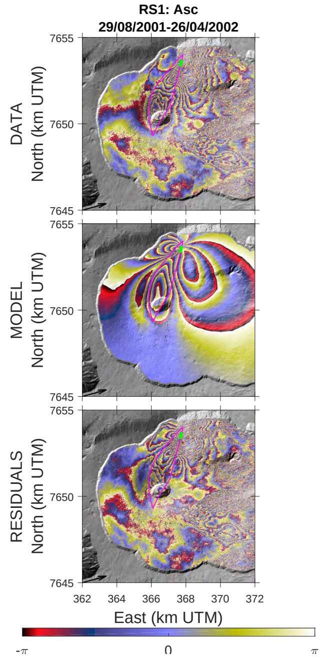

(**b**) Observed, modeled and residual displacements on wrapped data. Best model contour is in magenta and eruptive fissures are indicated by green lines. Line of sight of satellites acquisition is indicated by arrows.

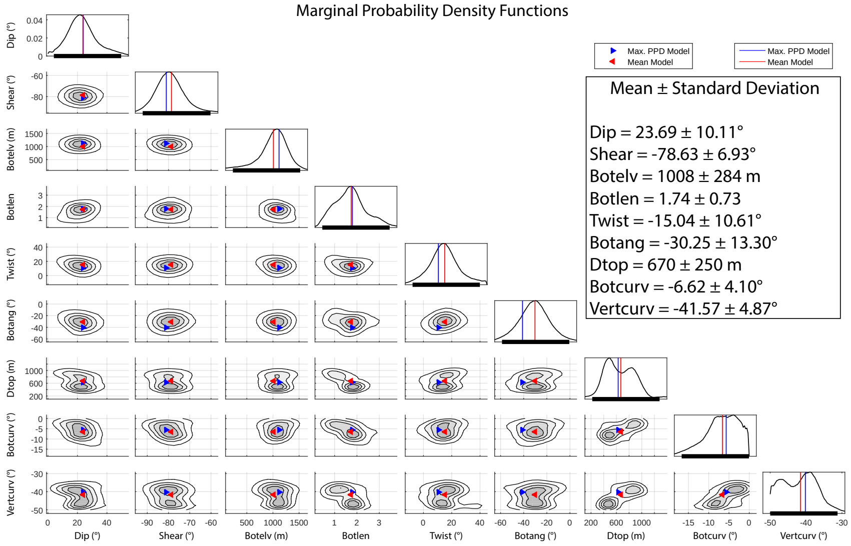

(**c**) Marginal posterior probability density functions. One-dimensional and two-dimensional functions are given in the diagonal and off-diagonal, respectively. Maximum and mean values are indicated by blue and red triangles, respectively. Black thick lines on one-dimensional functions represent the 95% confidence interval.

Fig. S14: Model for the 2002 January intrusion

November 2002 eruption

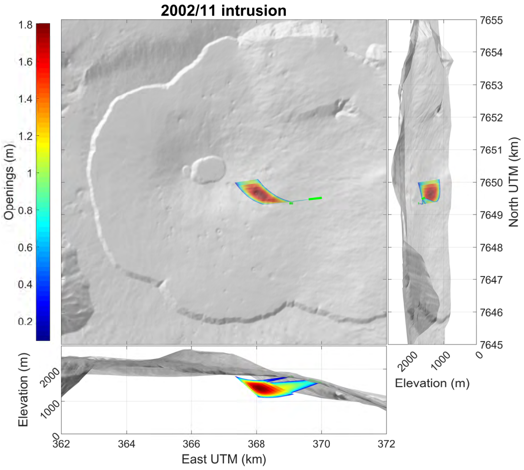

(a) 3D best geometry. Eruptive fissures are indicated by green lines.

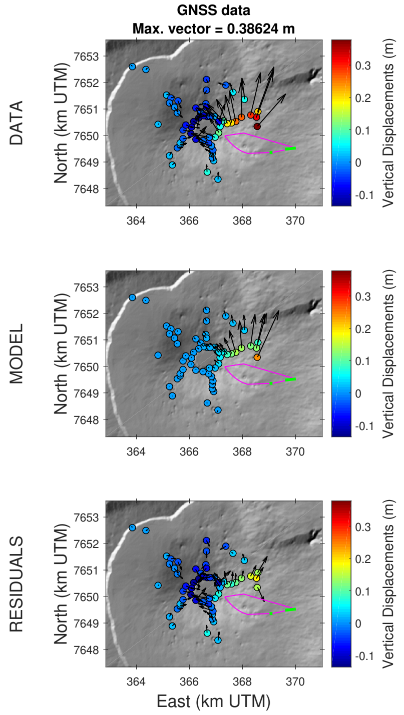

(b) Observed, modeled and residual displacements on GNSS reiteration network. Best model contour is in magenta and eruptive fissures are indicated by green lines.

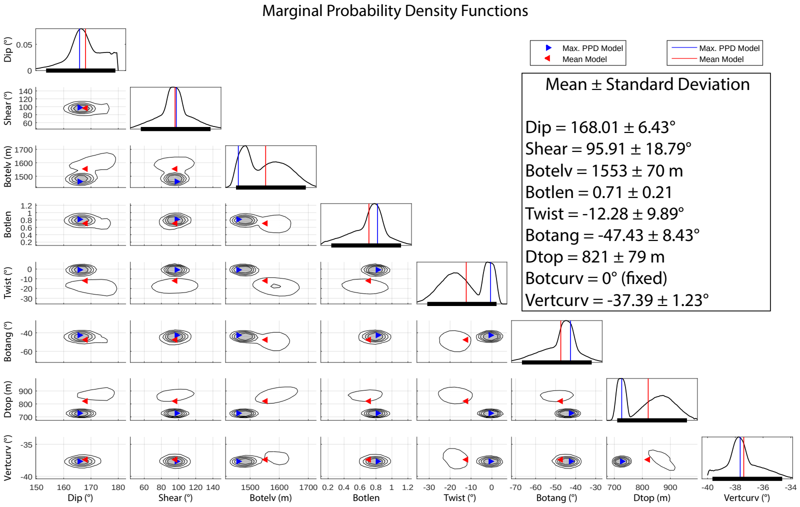

(c) Marginal posterior probability density functions. One-dimensional and two-dimensional functions are given in the diagonal and off-diagonal, respectively. Maximum and mean values are indicated by blue and red triangles, respectively. Black thick lines on one-dimensional functions represent the 95% confidence interval.

Fig. S15: Model for the 2002 November intrusion

May 2003 eruption

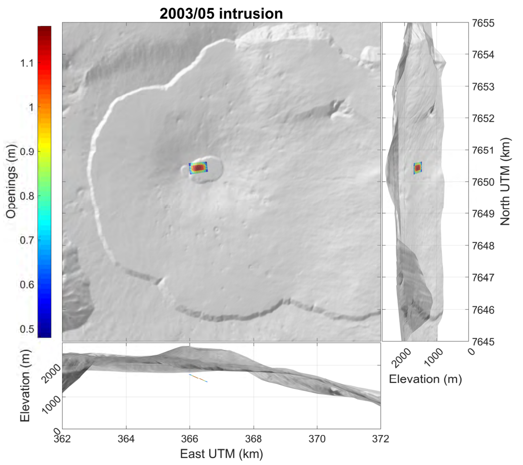

(**a**) 3D best geometry. Eruptive fissures are indicated by green lines.

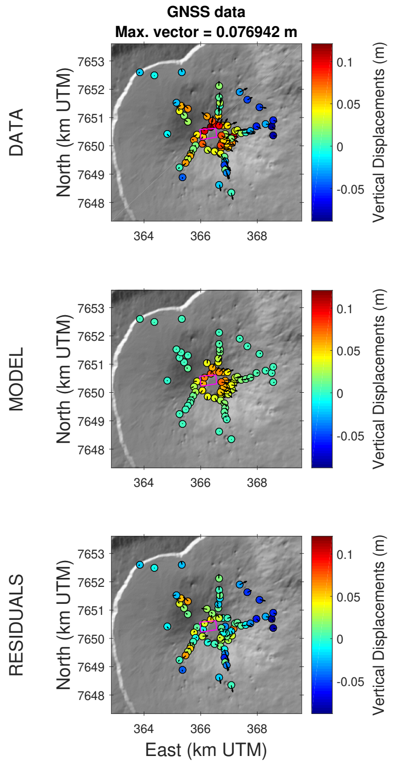

(**b**) Observed, modeled and residual displacements on wrapped data. Best model contour is in magenta and eruptive fissures are indicated by green lines. Line of sight of satellites acquisition is indicated by arrows.

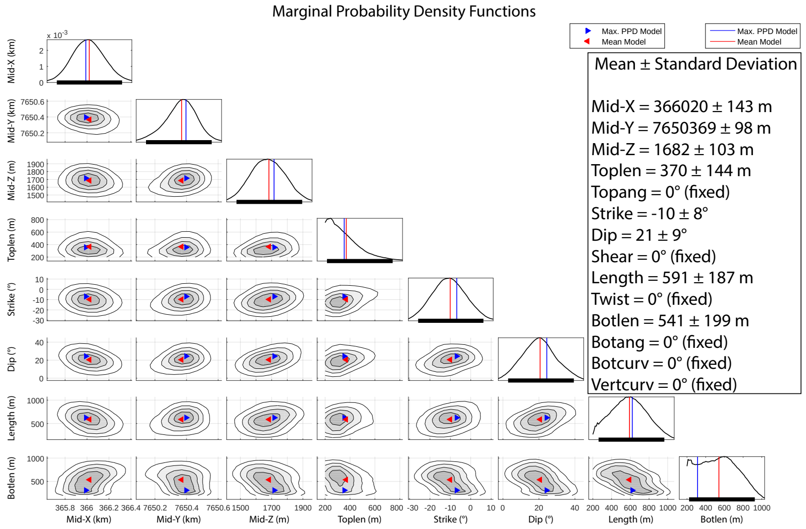

(**c**) Marginal posterior probability density functions. One-dimensional and two-dimensional functions are given in the diagonal and off-diagonal, respectively. Maximum and mean values are indicated by blue and red triangles, respectively. Black thick lines on one-dimensional functions represent the 95% confidence interval.

Fig. S16: Model for the 2003 May intrusion

August 2003 eruption

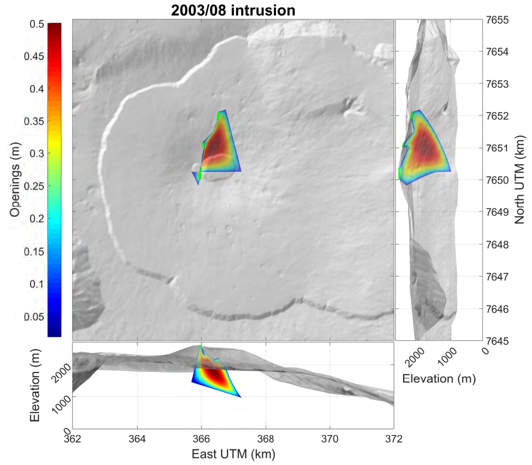

(a) 3D best geometry. Eruptive fissures are indicated by green lines.

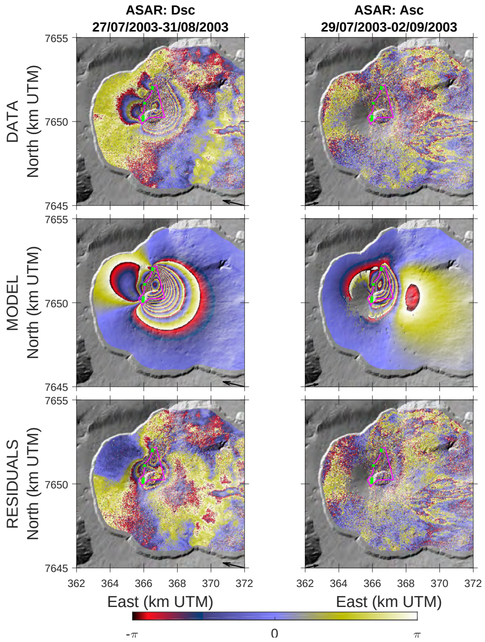

(b) Observed, modeled and residual displacements on wrapped data. Best model contour is in magenta and eruptive fissures are indicated by green lines. Line of sight of satellites acquisition is indicated by arrows.

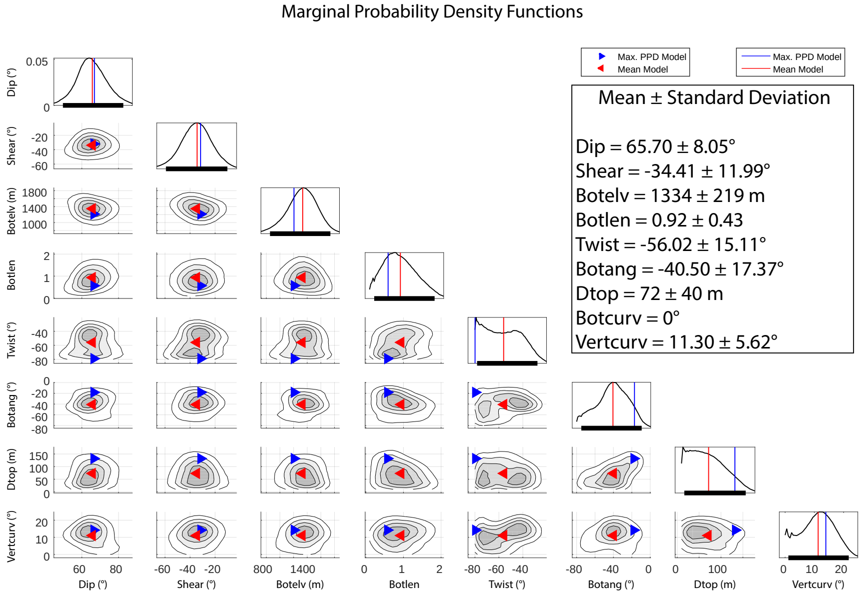

(c) Marginal posterior probability density functions. One-dimensional and two-dimensional functions are given in the diagonal and off-diagonal, respectively. Maximum and mean values are indicated by blue and red triangles, respectively. Black thick lines on one-dimensional functions represent the 95% confidence interval.

Fig. S17: Model for the 2003 August intrusion

September 2003 eruption

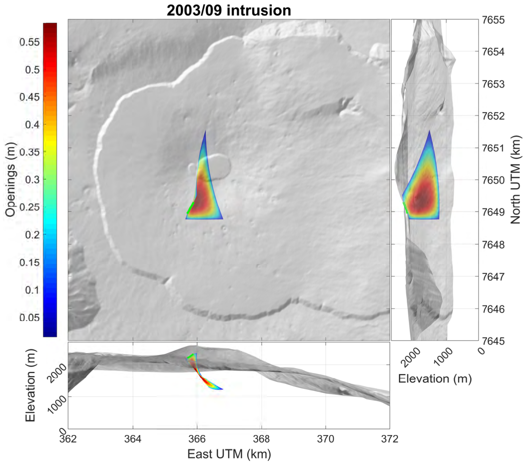

(a) 3D best geometry. Eruptive fissures are indicated by green lines.

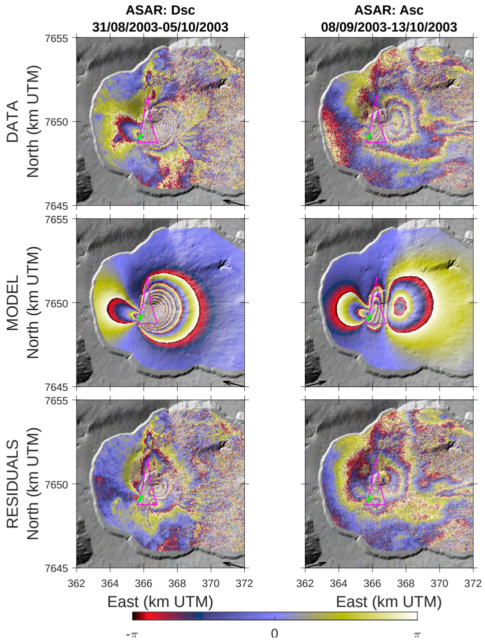

(b) Observed, modeled and residual displacements on wrapped data. Best model contour is in magenta and eruptive fissures are indicated by green lines. Line of sight of satellites acquisition is indicated by arrows.

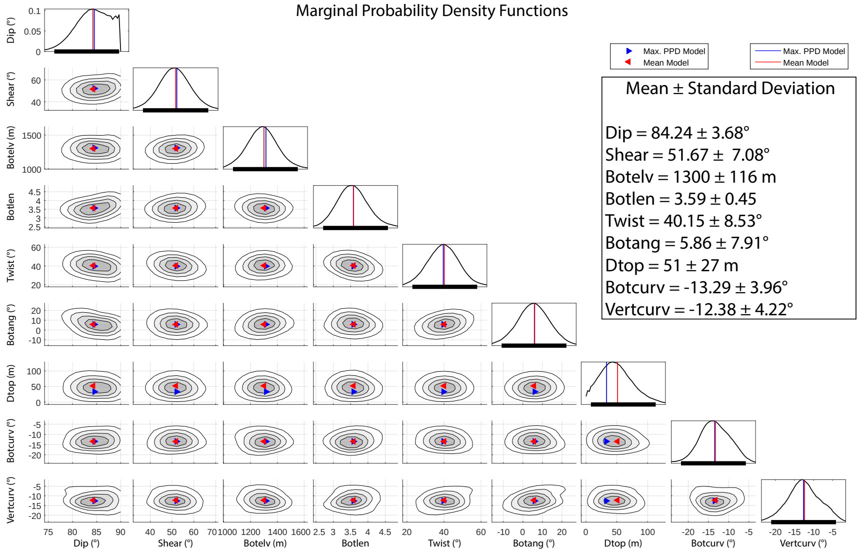

(c) Marginal posterior probability density functions. One-dimensional and two-dimensional functions are given in the diagonal and off-diagonal, respectively. Maximum and mean values are indicated by blue and red triangles, respectively. Black thick lines on one-dimensional functions represent the 95% confidence interval.

Fig. S18: Model for the 2003 September intrusion

November 2003 intrusion

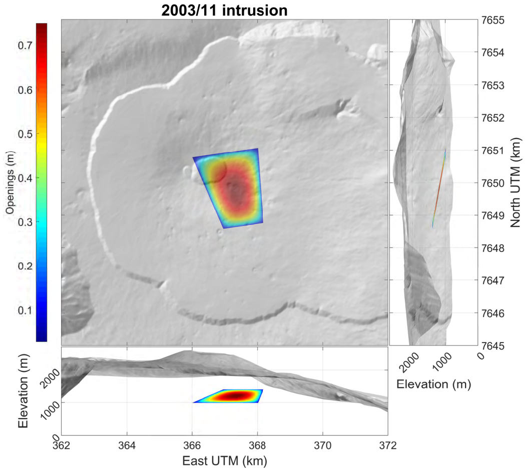

(a) 3D best geometry. Eruptive fissures are indicated by green lines.

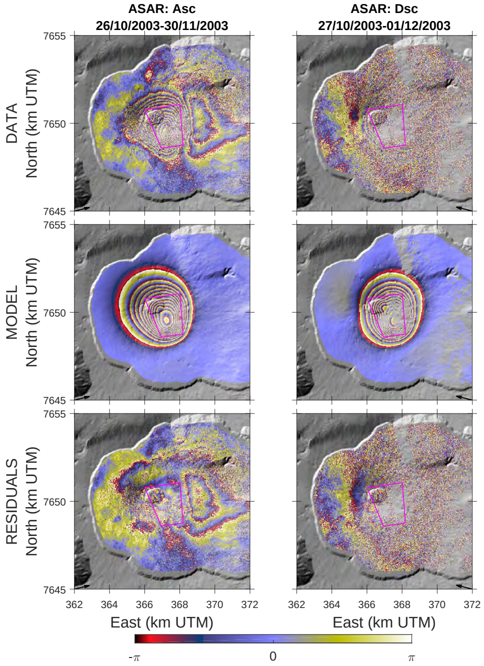

(b) Observed, modeled and residual displacements on wrapped data. Best model contour is in magenta and eruptive fissures are indicated by green lines. Line of sight of satellites acquisition is indicated by arrows.

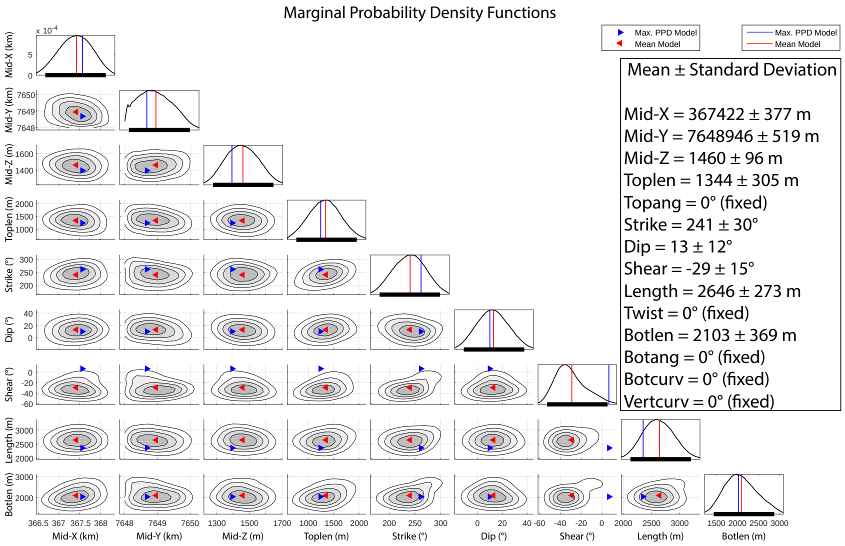

(c) Marginal posterior probability density functions. One-dimensional and two-dimensional functions are given in the diagonal and off-diagonal, respectively. Maximum and mean values are indicated by blue and red triangles, respectively. Black thick lines on one-dimensional functions represent the 95% confidence interval.

Fig. S19: Model for the 2003 November intrusion

December 2003 eruption

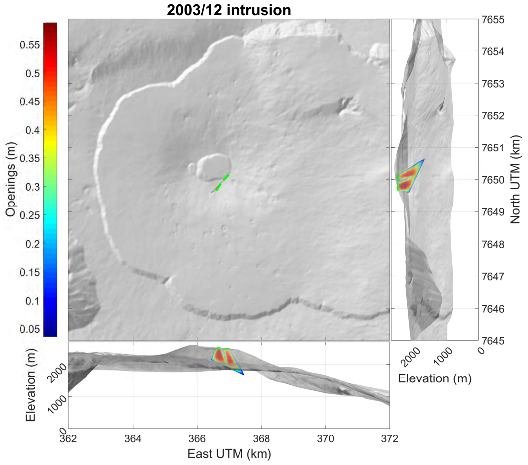

(a) 3D best geometry. Eruptive fissures are indicated by green lines.

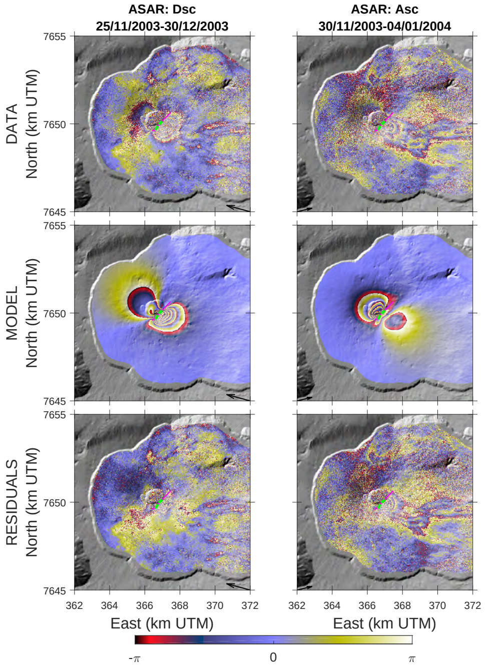

(b) Observed, modeled and residual displacements on wrapped data. Best model contour is in magenta and eruptive fissures are indicated by green lines. Line of sight of satellites acquisition is indicated by arrows.

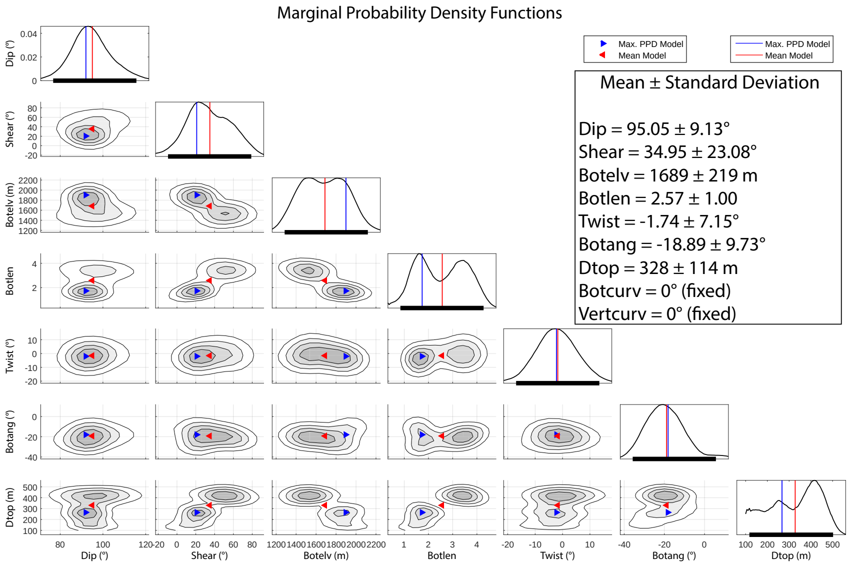

(c) Marginal posterior probability density functions. One-dimensional and two-dimensional functions are given in the diagonal and off-diagonal, respectively. Maximum and mean values are indicated by blue and red triangles, respectively. Black thick lines on one-dimensional functions represent the 95% confidence interval.

Fig. S20: Model for the 2003 December intrusion

January 2004 eruption

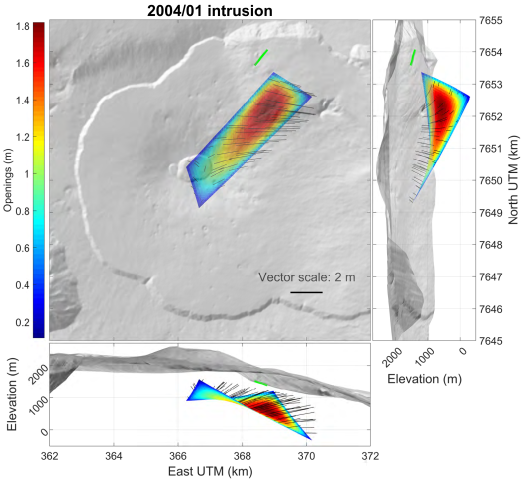

(**a**) 3D best geometry. Eruptive fissures are indicated by green lines.

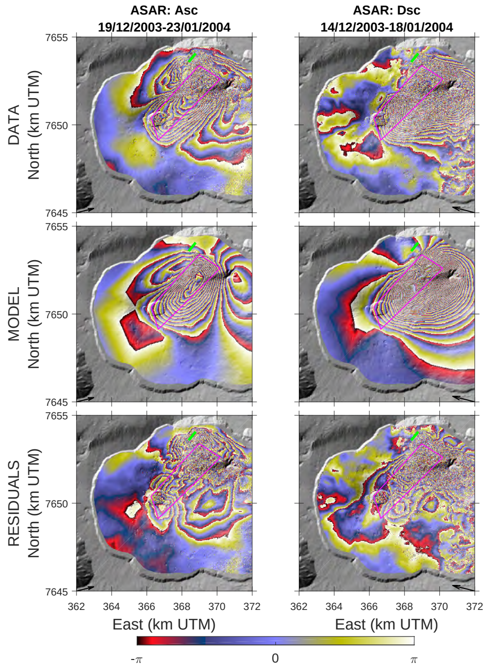

(**b**) Observed, modeled and residual displacements on wrapped data. Best model contour is in magenta and eruptive fissures are indicated by green lines. Line of sight of satellites acquisition is indicated by arrows.

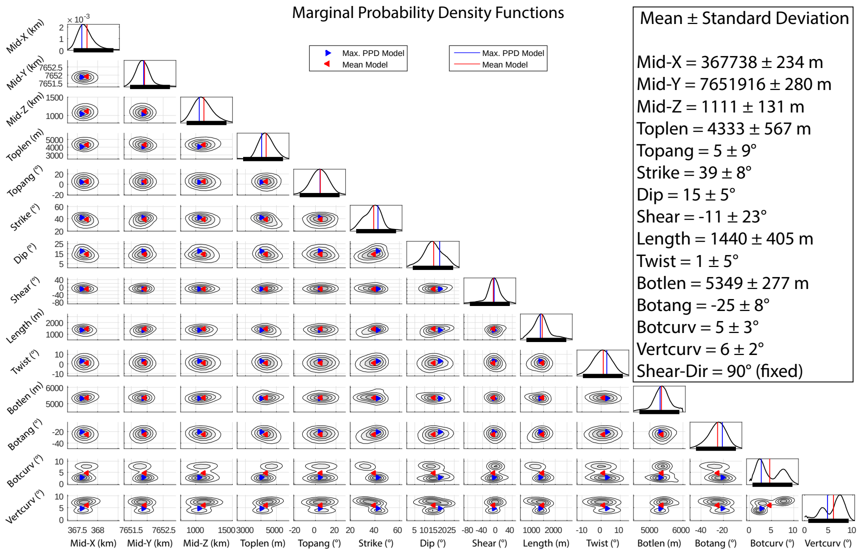

(**c**) Marginal posterior probability density functions. One-dimensional and two-dimensional functions are given in the diagonal and off-diagonal, respectively. Maximum and mean values are indicated by blue and red triangles, respectively. Black thick lines on one-dimensional functions represent the 95% confidence interval.

Fig. S21: Model for the 2004 January intrusion

May 2004 eruption

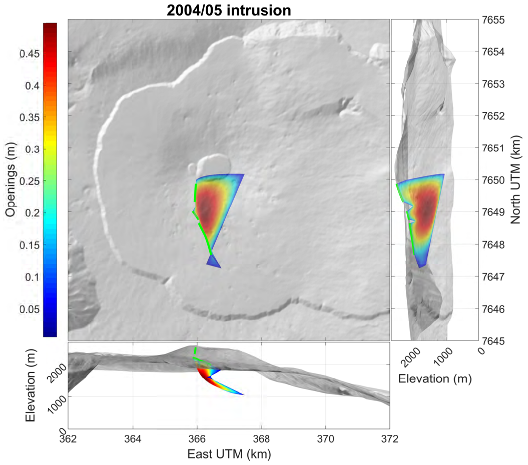

(a) 3D best geometry. Eruptive fissures are indicated by green lines.

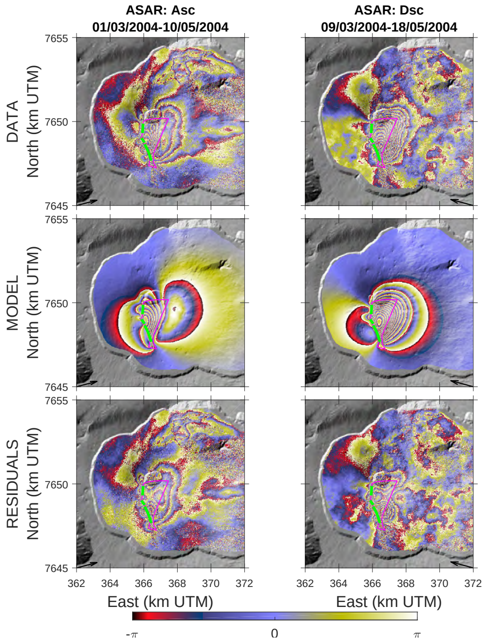

(b) Observed, modeled and residual displacements on wrapped data. Best model contour is in magenta and eruptive fissures are indicated by green lines. Line of sight of satellites acquisition is indicated by arrows.

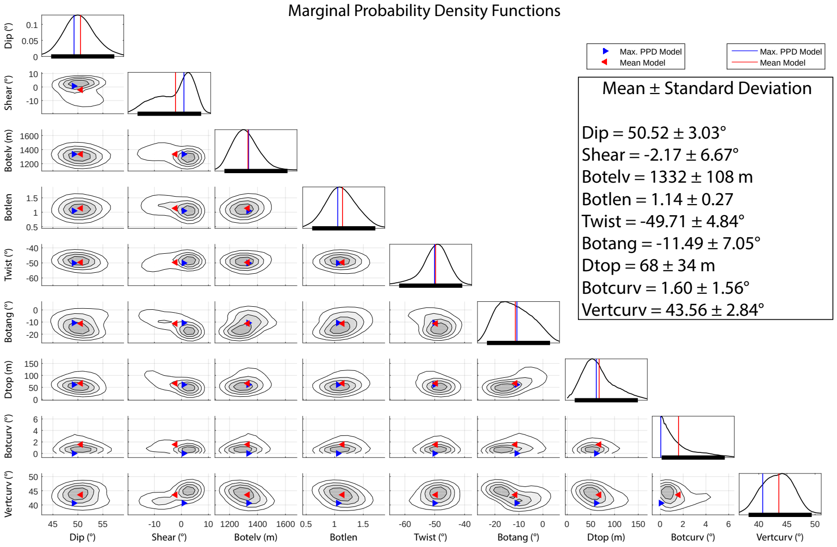

(c) Marginal posterior probability density functions. One-dimensional and two-dimensional functions are given in the diagonal and off-diagonal, respectively. Maximum and mean values are indicated by blue and red triangles, respectively. Black thick lines on one-dimensional functions represent the 95% confidence interval.

Fig. S22: Model for the 2004 May intrusion

August 2004 eruption

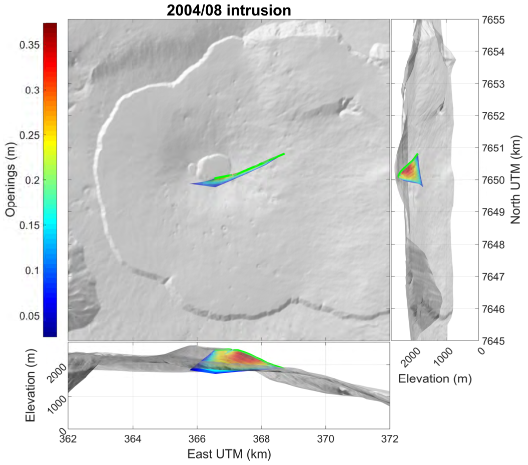

(a) 3D best geometry. Eruptive fissures are indicated by green lines.

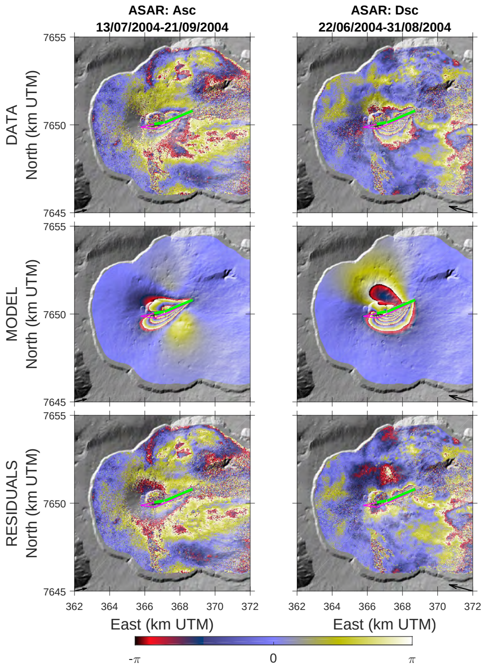

(b) Observed, modeled and residual displacements on wrapped data. Best model contour is in magenta and eruptive fissures are indicated by green lines. Line of sight of satellites acquisition is indicated by arrows.

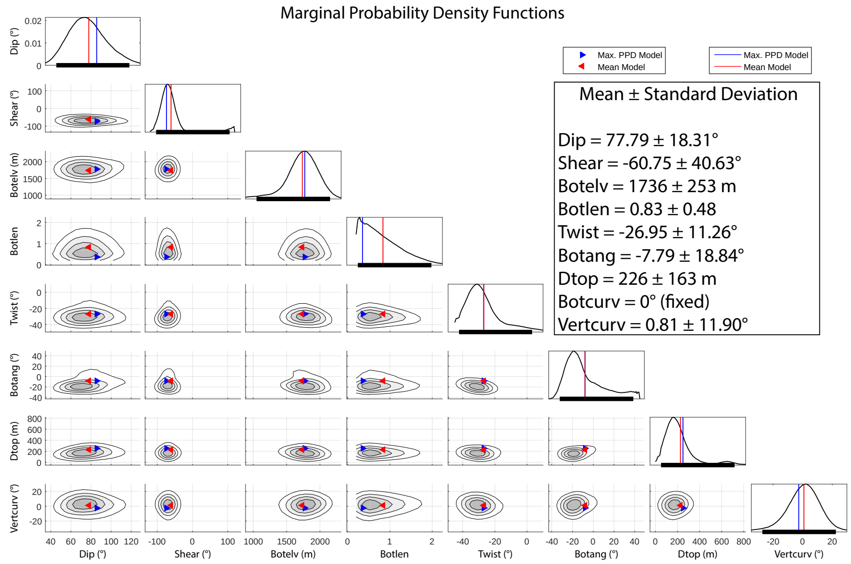

(c) Marginal posterior probability density functions. One-dimensional and two-dimensional functions are given in the diagonal and off-diagonal, respectively. Maximum and mean values are indicated by blue and red triangles, respectively. Black thick lines on one-dimensional functions represent the 95% confidence interval.

Fig. S23: Model for the 2004 August intrusion

February 2005 eruption

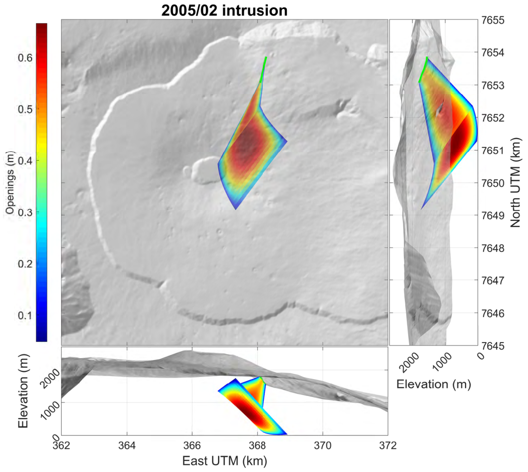

(a) 3D best geometry. Eruptive fissures are indicated by green lines.

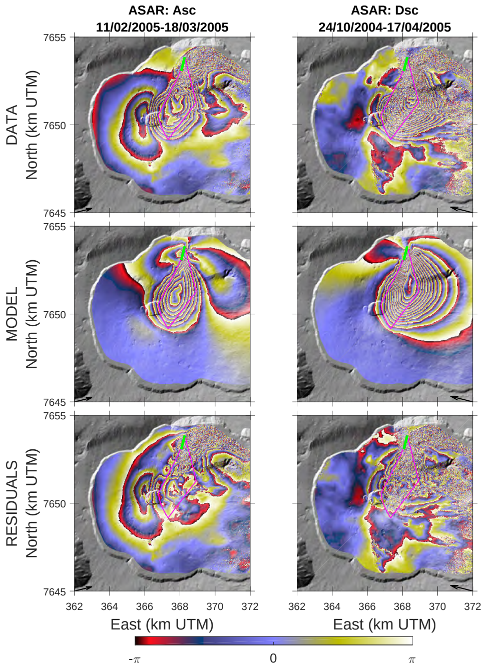

(b) Observed, modeled and residual displacements on wrapped data. Best model contour is in magenta and eruptive fissures are indicated by green lines. Line of sight of satellites acquisition is indicated by arrows.

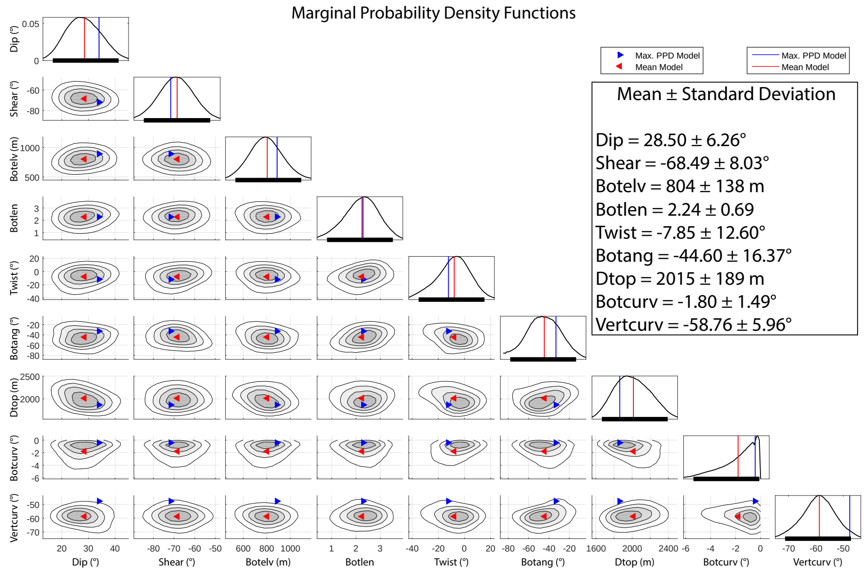

(c) Marginal posterior probability density functions. One-dimensional and two-dimensional functions are given in the diagonal and off-diagonal, respectively. Maximum and mean values are indicated by blue and red triangles, respectively. Black thick lines on one-dimensional functions represent the 95% confidence interval.

Fig. S24: Model for the 2005 February intrusion

October 2005 eruption

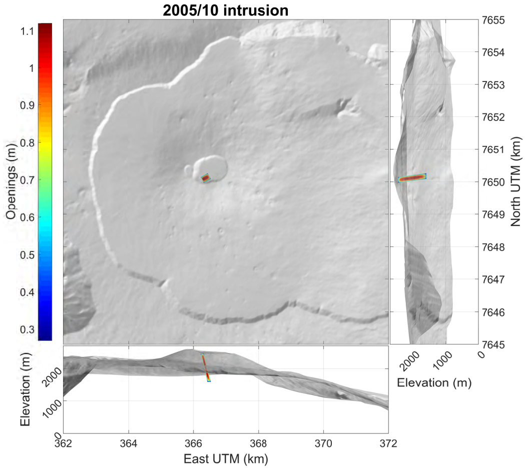

(a) 3D best geometry. Eruptive fissures are indicated by green lines.

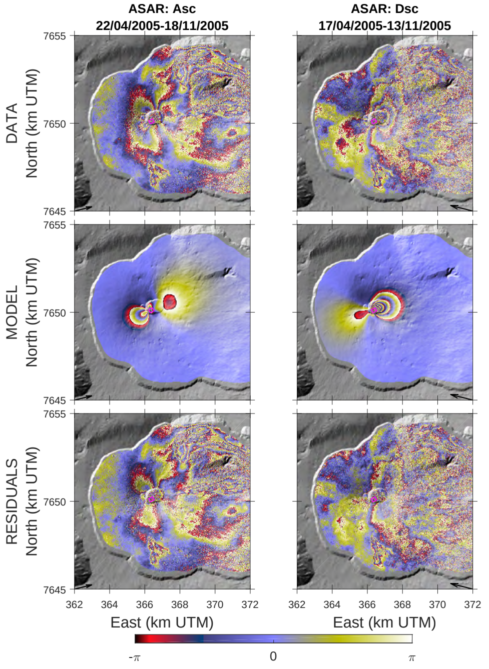

(b) Observed, modeled and residual displacements on wrapped data. Best model contour is in magenta and eruptive fissures are indicated by green lines. Line of sight of satellites acquisition is indicated by arrows.

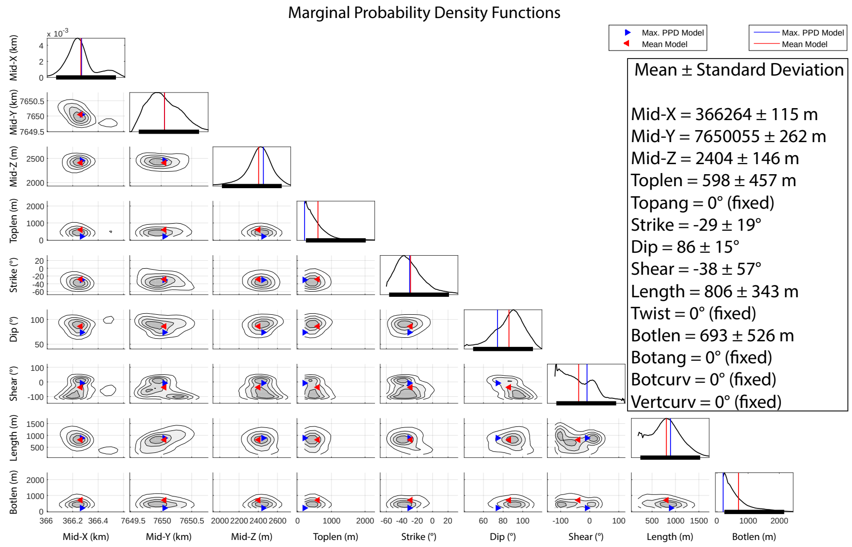

(c) Marginal posterior probability density functions. One-dimensional and two-dimensional functions are given in the diagonal and off-diagonal, respectively. Maximum and mean values are indicated by blue and red triangles, respectively. Black thick lines on one-dimensional functions represent the 95% confidence interval.

Fig. S25: Model for the 2005 October intrusion

November 2005 eruption

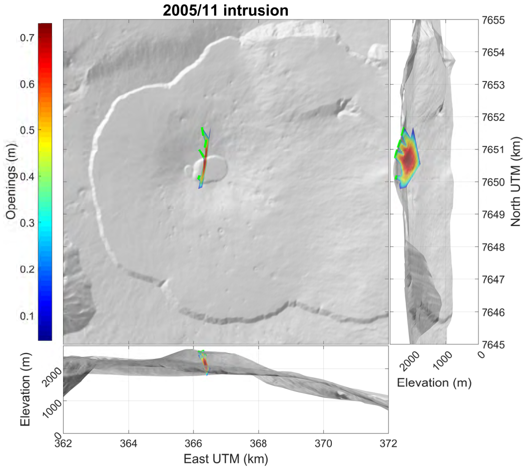

(a) 3D best geometry. Eruptive fissures are indicated by green lines.

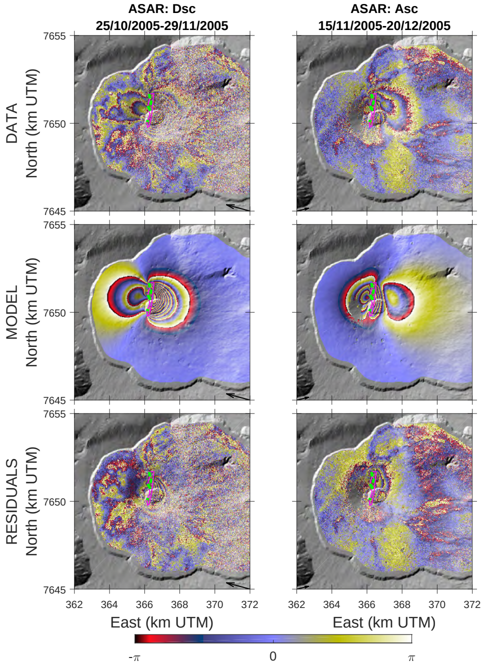

(b) Observed, modeled and residual displacements on wrapped data. Best model contour is in magenta and eruptive fissures are indicated by green lines. Line of sight of satellites acquisition is indicated by arrows.

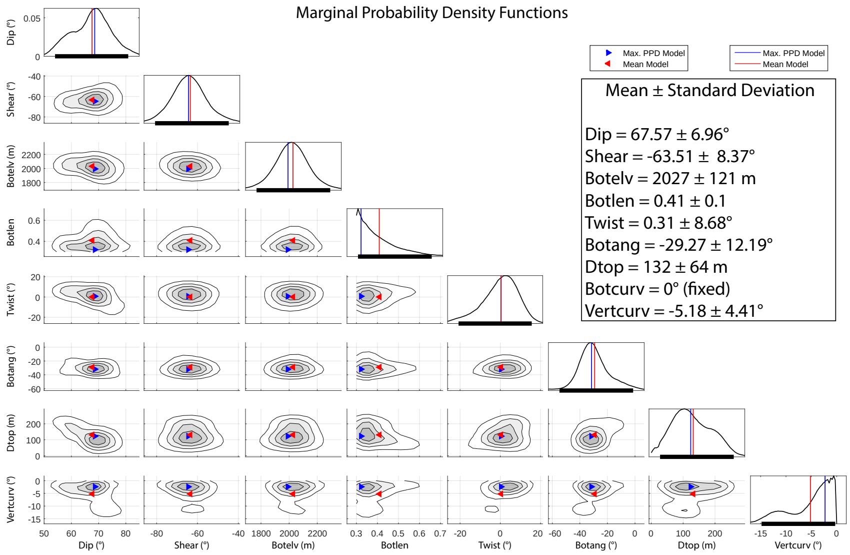

(c) Marginal posterior probability density functions. One-dimensional and two-dimensional functions are given in the diagonal and off-diagonal, respectively. Maximum and mean values are indicated by blue and red triangles, respectively. Black thick lines on one-dimensional functions represent the 95% confidence interval.

Fig. S26: Model for the 2005 November intrusion

December 2005 eruption

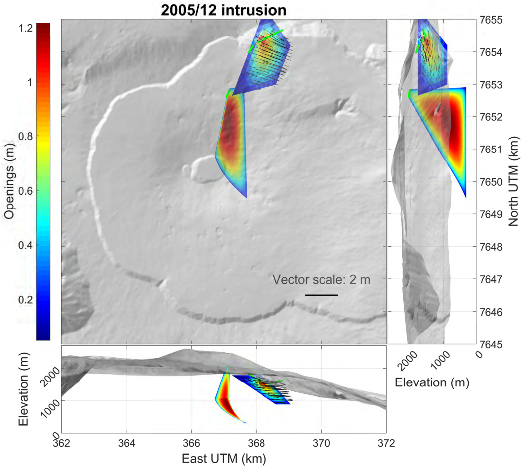

(**a**) 3D best geometry. Eruptive fissures are indicated by green lines.

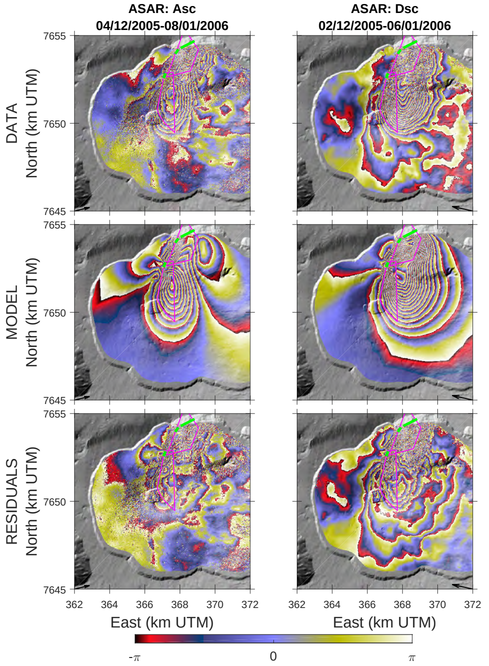

(**b**) Observed, modeled and residual displacements on wrapped data. Best model contour is in magenta and eruptive fissures are indicated by green lines. Line of sight of satellites acquisition is indicated by arrows.

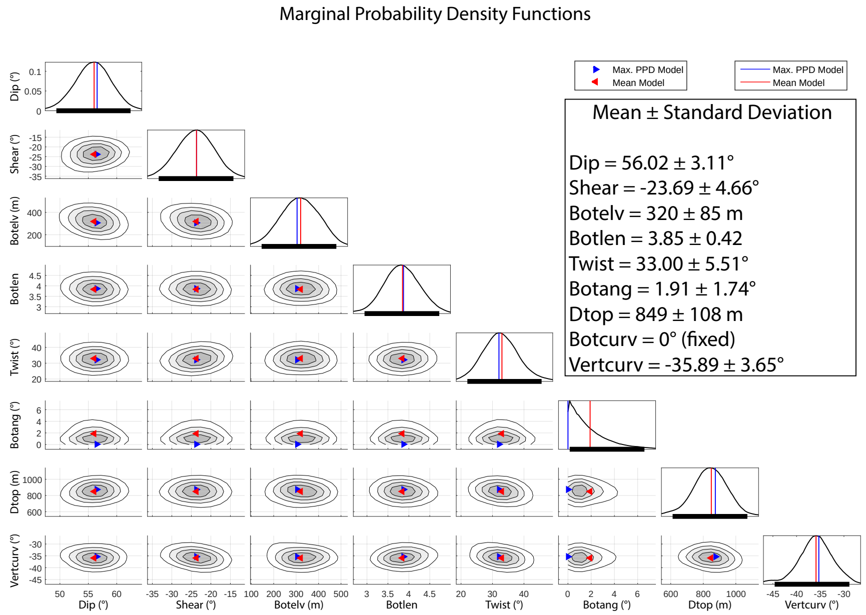

(**c**) Marginal posterior probability density functions for the dyke intrusion. One-dimensional and two-dimensional functions are given in the diagonal and off-diagonal, respectively. Maximum and mean values are indicated by blue and red triangles, respectively. Black thick lines on one-dimensional functions represent the 95% confidence interval.

Fig. S27: Model for the 2005 December intrusion

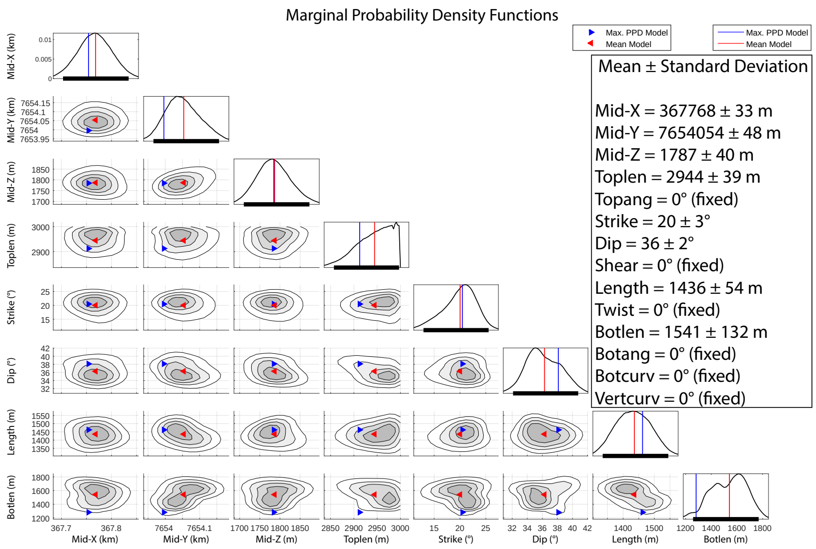

(d) Marginal posterior probability density functions for the sheared dyke of Plaine des Osmondes. One-dimensional and two-dimensional functions are given in the diagonal and off-diagonal, respectively. Maximum and mean values are indicated by blue and red triangles, respectively. Black thick lines on one-dimensional functions represent the 95% confidence interval.

Fig. S27 (Continued): Model for the 2005 December intrusion

July 2006 eruption

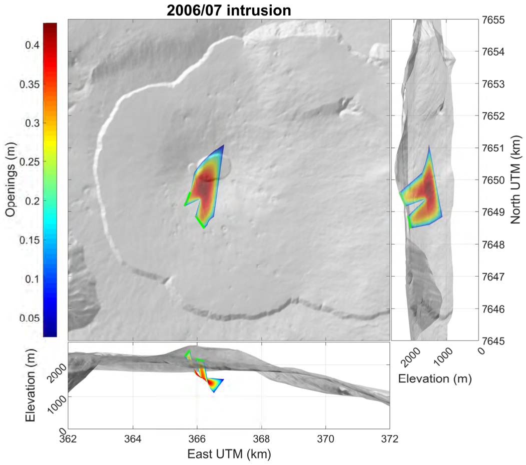

(**a**) 3D best geometry. Eruptive fissures are indicated by green lines.

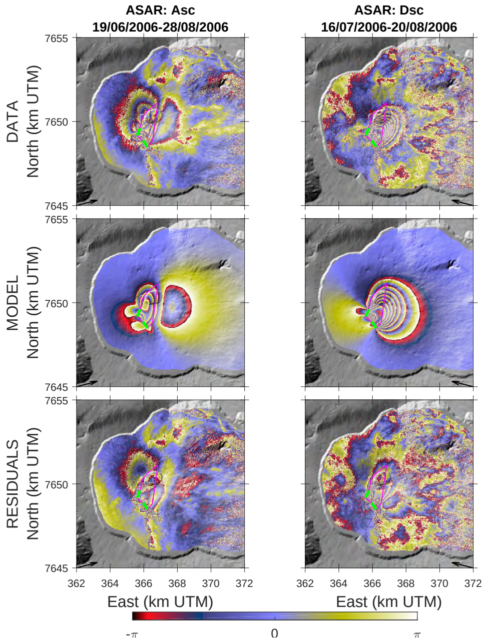

(**b**) Observed, modeled and residual displacements on wrapped data. Best model contour is in magenta and eruptive fissures are indicated by green lines. Line of sight of satellites acquisition is indicated by arrows.

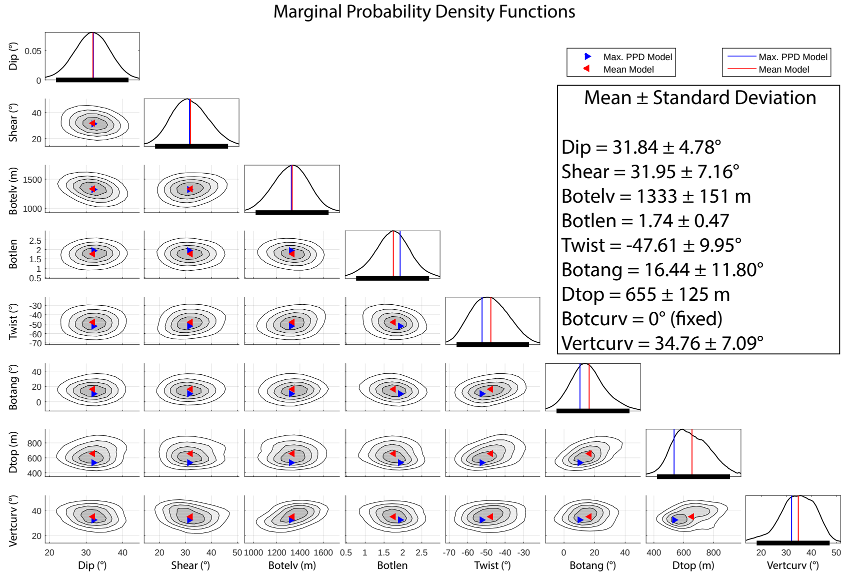

(**c**) Marginal posterior probability density functions. One-dimensional and two-dimensional functions are given in the diagonal and off-diagonal, respectively. Maximum and mean values are indicated by blue and red triangles, respectively. Black thick lines on one-dimensional functions represent the 95% confidence interval.

Fig. S28: Model for the 2006 July intrusion

August 2006 eruption

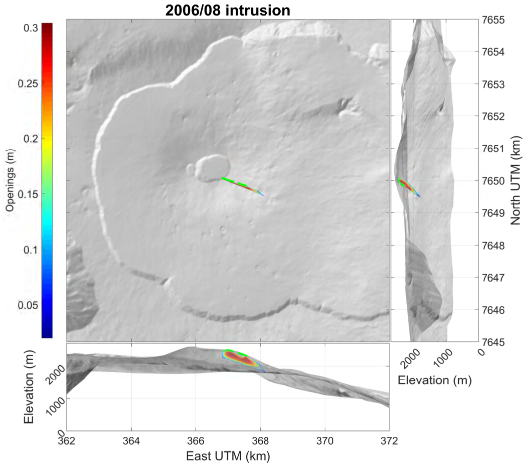

(a) 3D best geometry. Eruptive fissures are indicated by green lines.

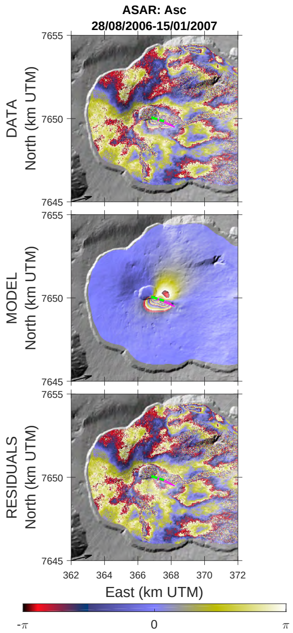

(b) Observed, modeled and residual displacements on wrapped data. Best model contour is in magenta and eruptive fissures are indicated by green lines. Line of sight of satellites acquisition is indicated by arrows.

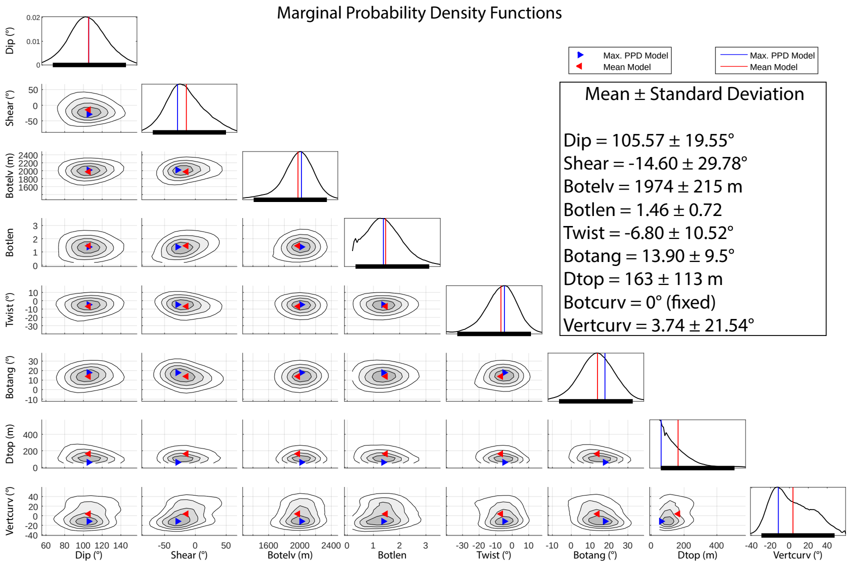

(c) Marginal posterior probability density functions. One-dimensional and two-dimensional functions are given in the diagonal and off-diagonal, respectively. Maximum and mean values are indicated by blue and red triangles, respectively. Black thick lines on one-dimensional functions represent the 95% confidence interval.

Fig. S29: Model for the 2006 August intrusion

February 2007 eruption

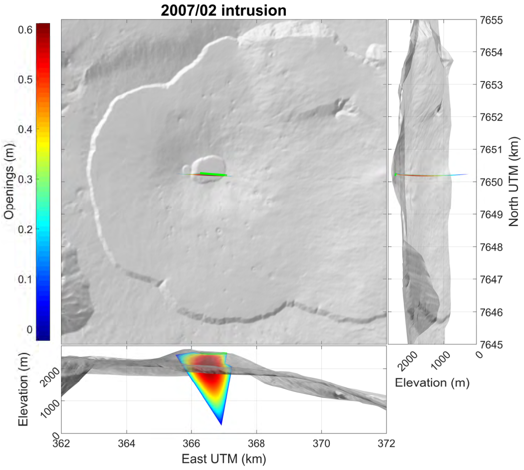

(**a**) 3D best geometry. Eruptive fissures are indicated by green lines.

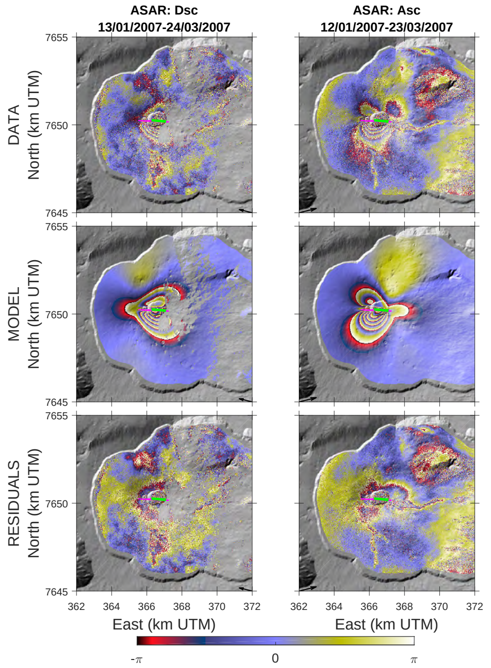

(**b**) Observed, modeled and residual displacements on wrapped data. Best model contour is in magenta and eruptive fissures are indicated by green lines. Line of sight of satellites acquisition is indicated by arrows.

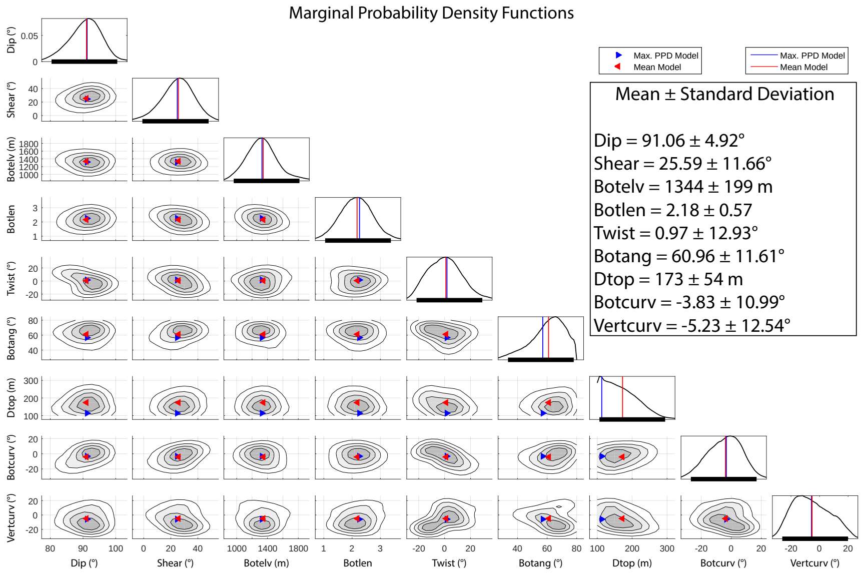

(**c**) Marginal posterior probability density functions. One-dimensional and two-dimensional functions are given in the diagonal and off-diagonal, respectively. Maximum and mean values are indicated by blue and red triangles, respectively. Black thick lines on one-dimensional functions represent the 95% confidence interval.

Fig. S30: Model for the 2007 February intrusion

March/April 2007 eruption, model from Tridon et al. [3] and Cayol et al. [4]

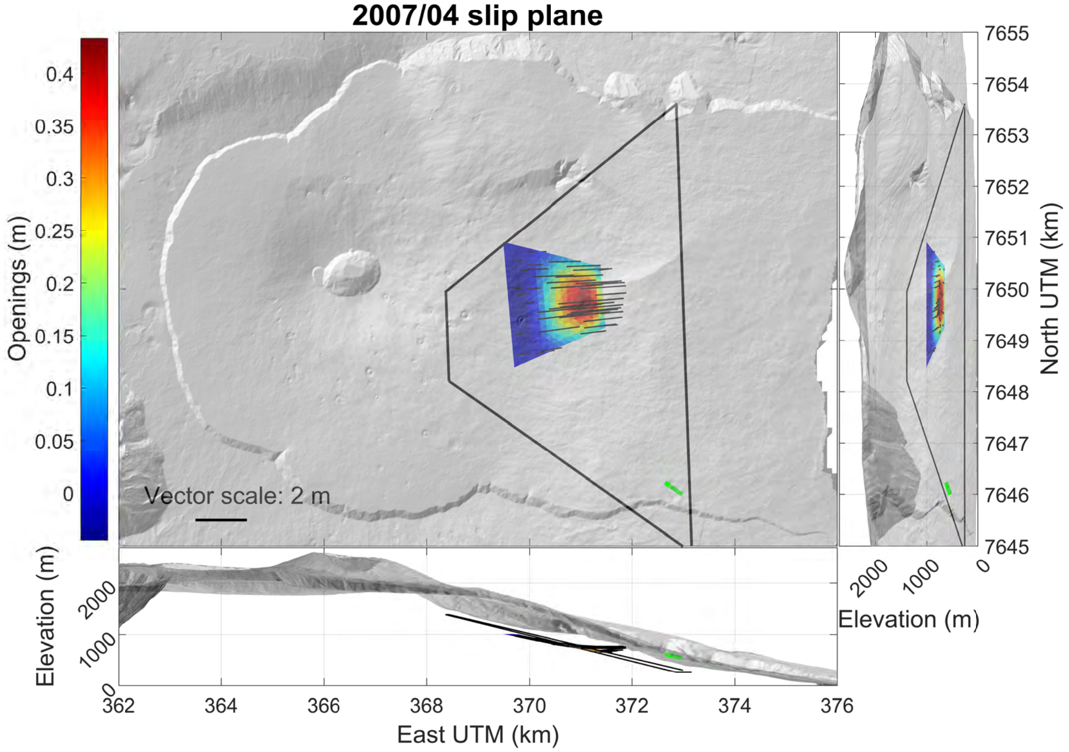

Fig. S31: Model for the 2007 April slip. Eruptive fissures is indicated by the green line. The black contour indicates the best-fitting geometry of the post-eruptive displacements.

September 2008 eruption

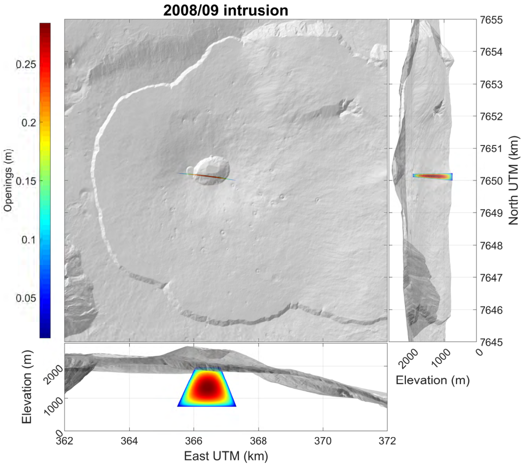

(a) 3D best geometry. Eruptive fissures are indicated by green lines.

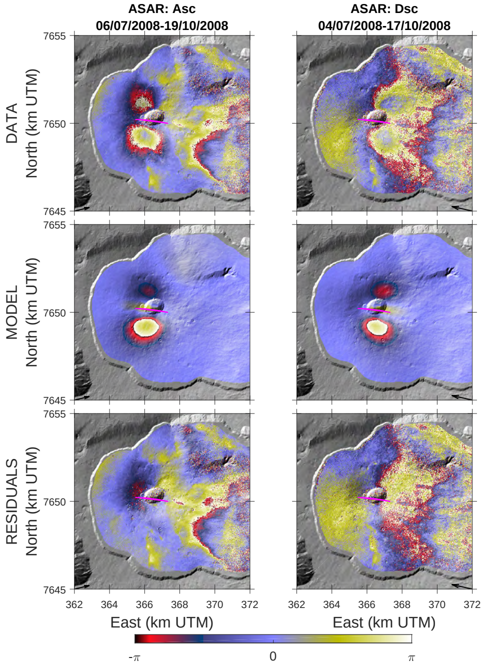

(b) Observed, modeled and residual displacements on wrapped data. Best model contour is in magenta and eruptive fissures are indicated by green lines. Line of sight of satellites acquisition is indicated by arrows.

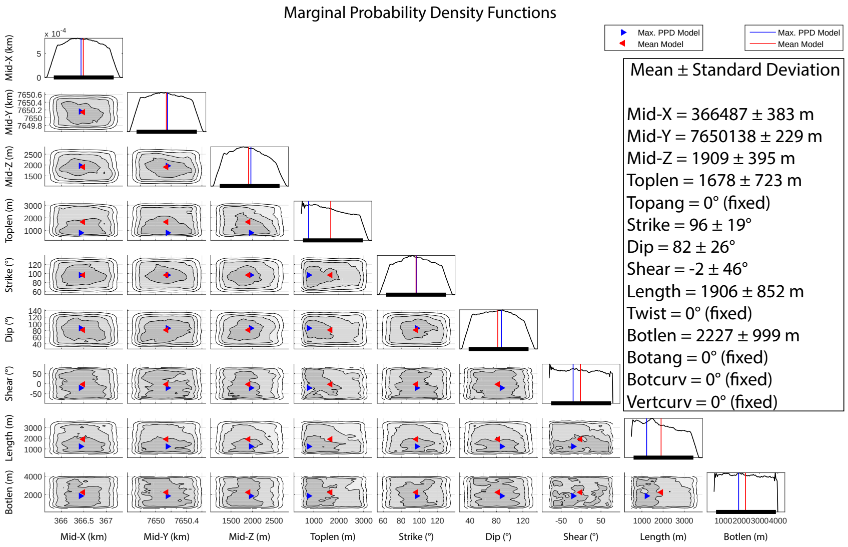

(c) Marginal posterior probability density functions. One-dimensional and two-dimensional functions are given in the diagonal and off-diagonal, respectively. Maximum and mean values are indicated by blue and red triangles, respectively. Black thick lines on one-dimensional functions represent the 95% confidence interval.

Fig. S32: Model for the 2008 September intrusion

November 2008 eruption

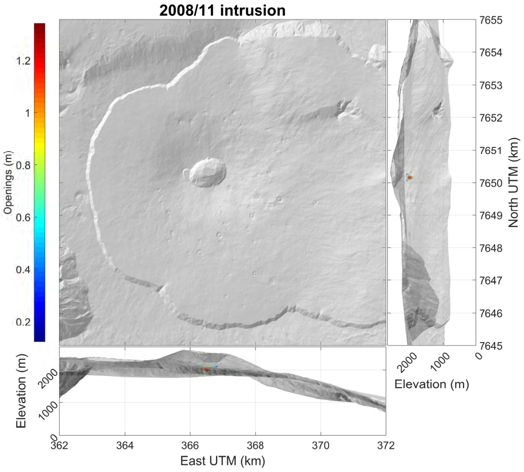

(a) 3D best geometry. Eruptive fissures are indicated by green lines.

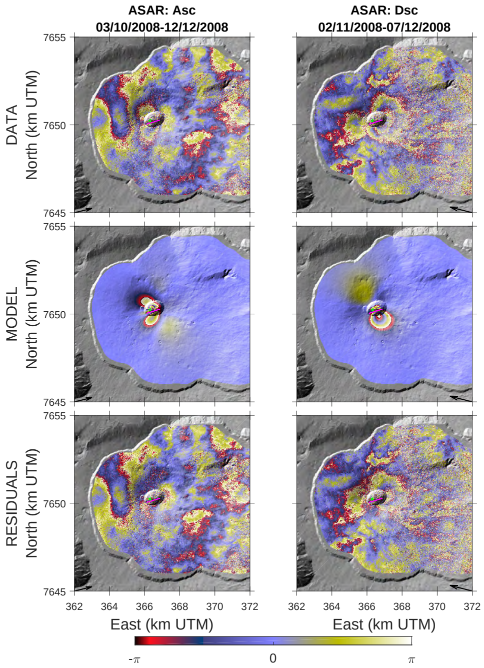

(b) Observed, modeled and residual displacements on wrapped data. Best model contour is in magenta and eruptive fissures are indicated by green lines. Line of sight of satellites acquisition is indicated by arrows.

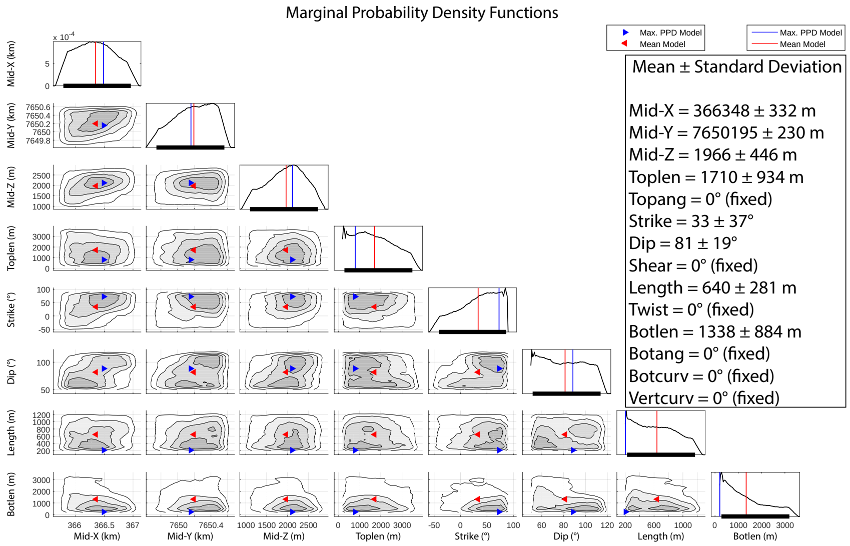

(c) Marginal posterior probability density functions. One-dimensional and two-dimensional functions are given in the diagonal and off-diagonal, respectively. Maximum and mean values are indicated by blue and red triangles, respectively. Black thick lines on one-dimensional functions represent the 95% confidence interval.

Fig. S33: Model for the 2008 November intrusion

December 2008 eruption

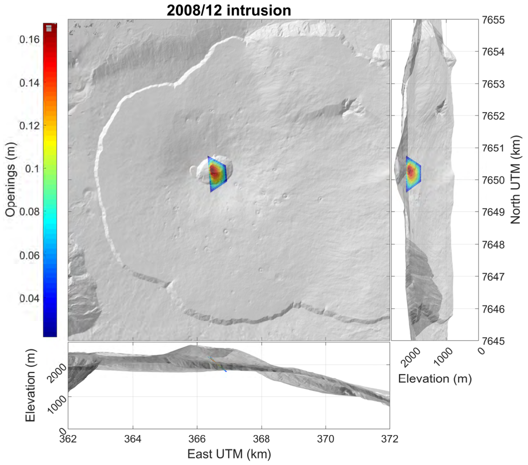

(a) 3D best geometry. Eruptive fissures are indicated by green lines.

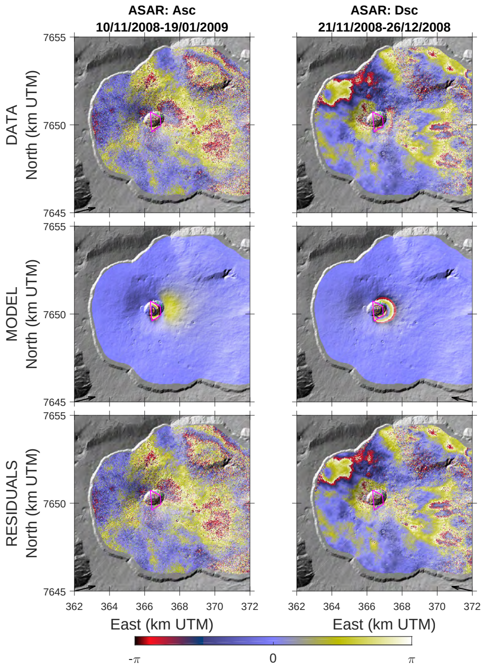

(b) Observed, modeled and residual displacements on wrapped data. Best model contour is in magenta and eruptive fissures are indicated by green lines. Line of sight of satellites acquisition is indicated by arrows.

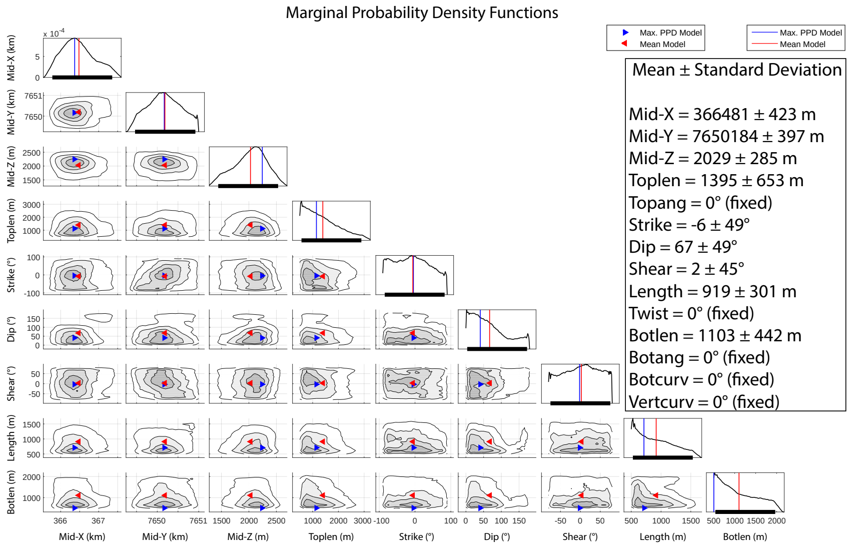

(c) Marginal posterior probability density functions. One-dimensional and two-dimensional functions are given in the diagonal and off-diagonal, respectively. Maximum and mean values are indicated by blue and red triangles, respectively. Black thick lines on one-dimensional functions represent the 95% confidence interval.

Fig. S34: Model for the 2008 December intrusion

November 2009 eruption

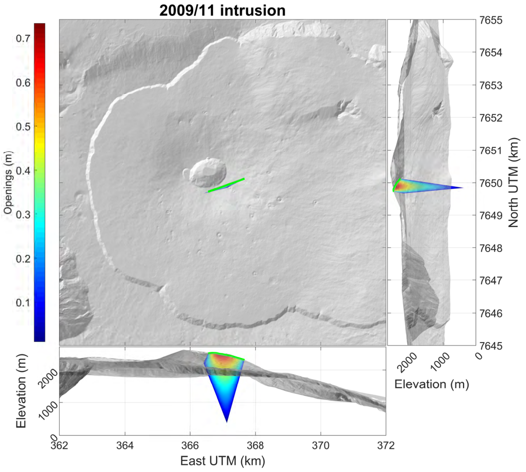

(a) 3D best geometry. Eruptive fissures are indicated by green lines.

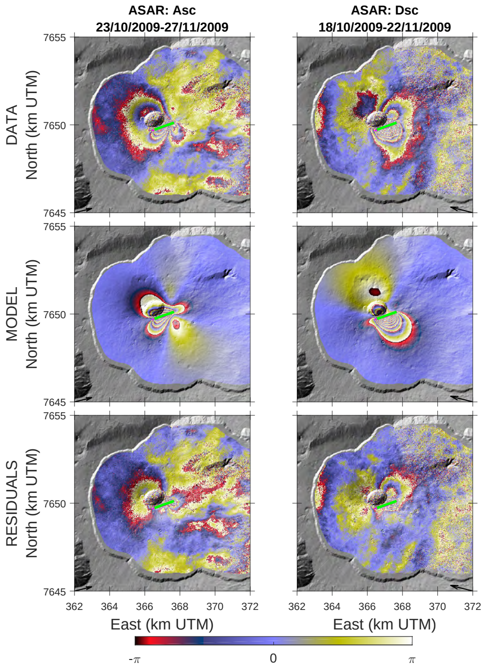

(b) Observed, modeled and residual displacements on wrapped data. Best model contour is in magenta and eruptive fissures are indicated by green lines. Line of sight of satellites acquisition is indicated by arrows.

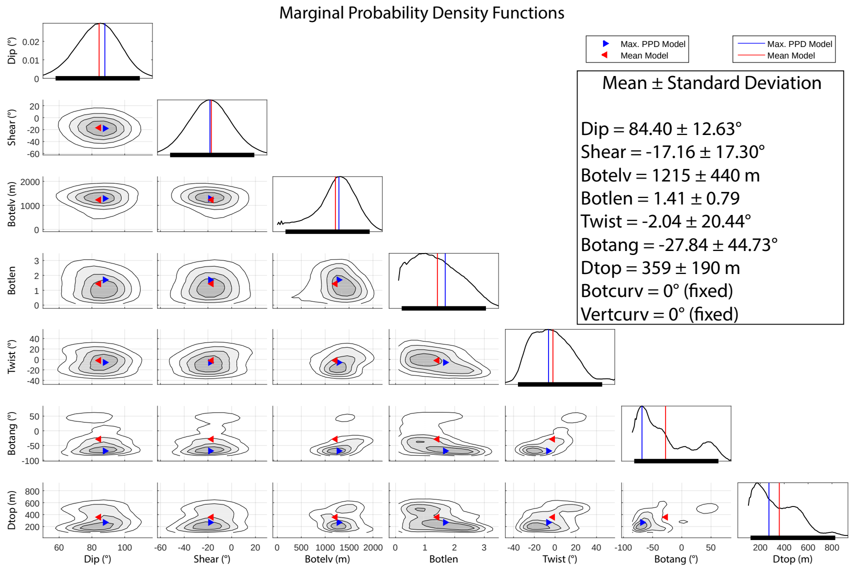

(c) Marginal posterior probability density functions. One-dimensional and two-dimensional functions are given in the diagonal and off-diagonal, respectively. Maximum and mean values are indicated by blue and red triangles, respectively. Black thick lines on one-dimensional functions represent the 95% confidence interval.

Fig. S35: Model for the 2009 November intrusion

December 2009 eruption

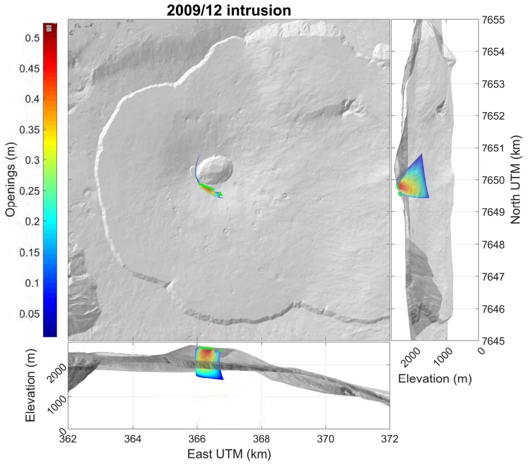

(a) 3D best geometry. Eruptive fissures are indicated by green lines.

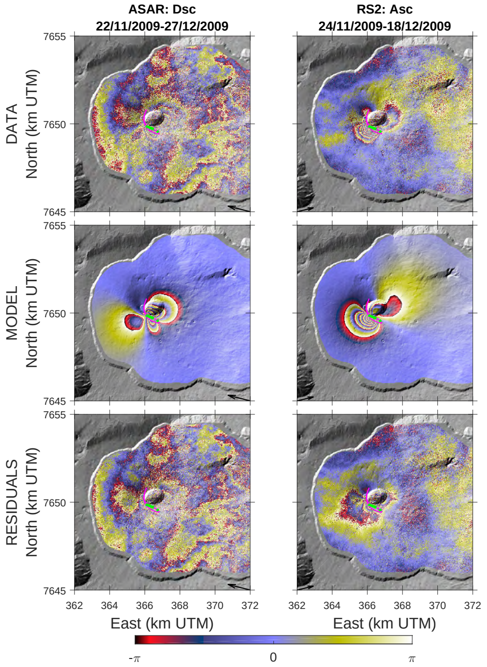

(b) Observed, modeled and residual displacements on wrapped data. Best model contour is in magenta and eruptive fissures are indicated by green lines. Line of sight of satellites acquisition is indicated by arrows.

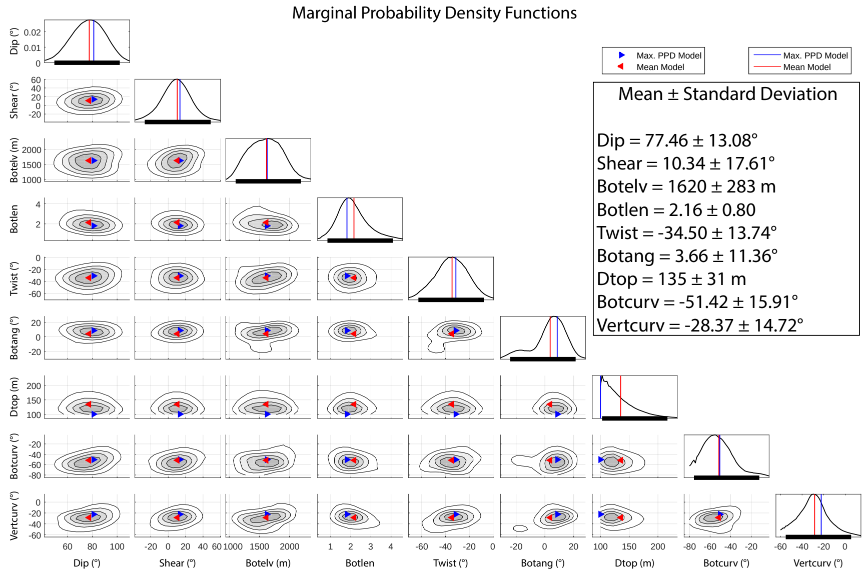

(c) Marginal posterior probability density functions. One-dimensional and two-dimensional functions are given in the diagonal and off-diagonal, respectively. Maximum and mean values are indicated by blue and red triangles, respectively. Black thick lines on one-dimensional functions represent the 95% confidence interval.

Fig. S36: Model for the 2009 December intrusion

January 2010 eruption

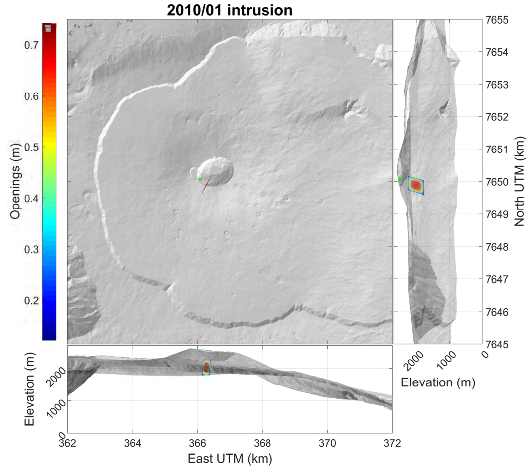

(**a**) 3D best geometry. Eruptive fissures are indicated by green lines.

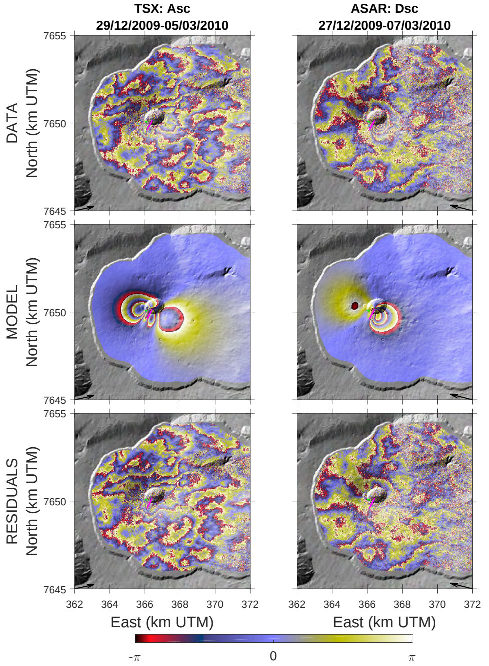

(**b**) Observed, modeled and residual displacements on wrapped data. Best model contour is in magenta and eruptive fissures are indicated by green lines. Line of sight of satellites acquisition is indicated by arrows.

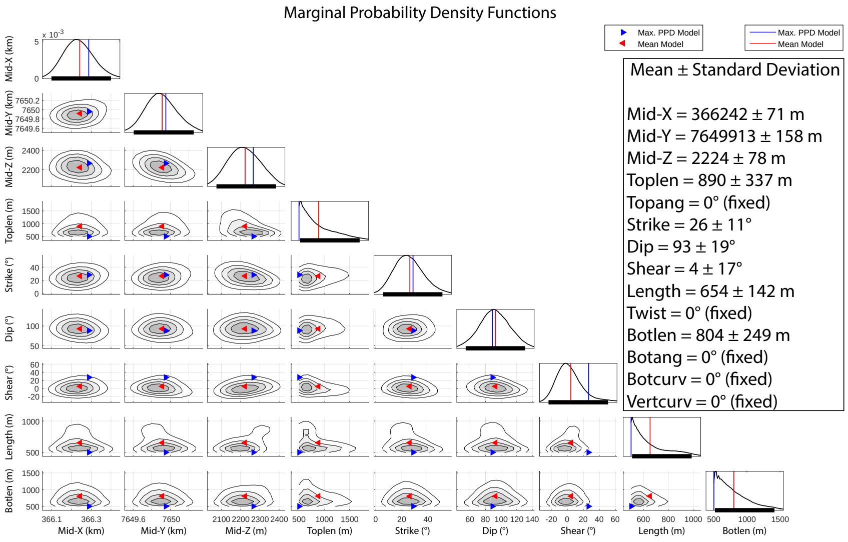

(**c**) Marginal posterior probability density functions. One-dimensional and two-dimensional functions are given in the diagonal and off-diagonal, respectively. Maximum and mean values are indicated by blue and red triangles, respectively. Black thick lines on one-dimensional functions represent the 95% confidence interval.

Fig. S37: Model for the 2010 January intrusion

October 2010 eruption

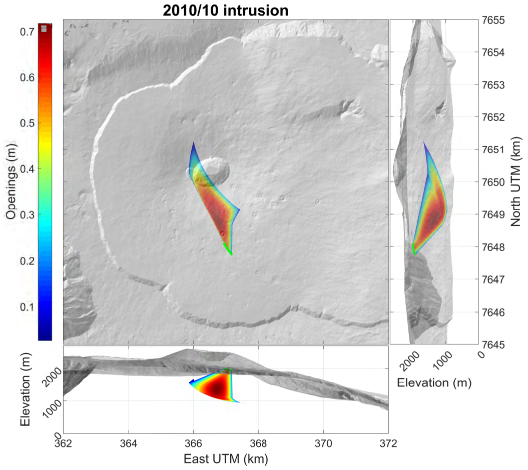

(a) 3D best geometry. Eruptive fissures are indicated by green lines.

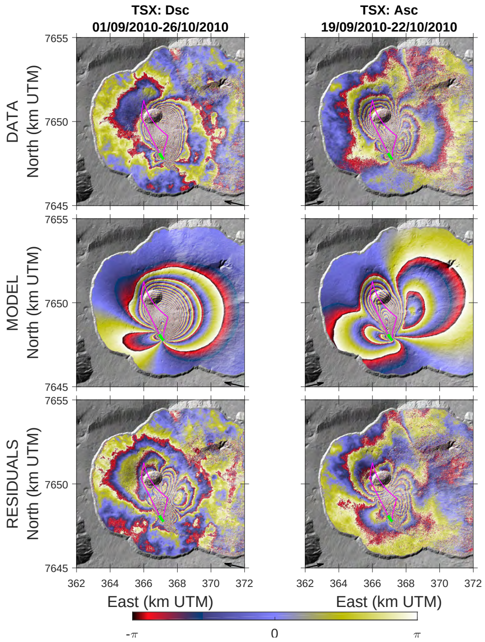

(b) Observed, modeled and residual displacements on wrapped data. Best model contour is in magenta and eruptive fissures are indicated by green lines. Line of sight of satellites acquisition is indicated by arrows.

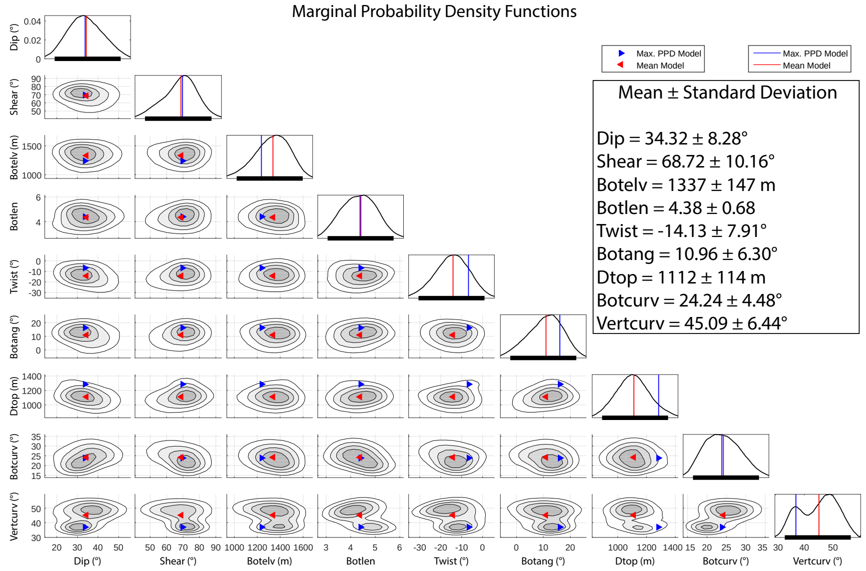

(c) Marginal posterior probability density functions. One-dimensional and two-dimensional functions are given in the diagonal and off-diagonal, respectively. Maximum and mean values are indicated by blue and red triangles, respectively. Black thick lines on one-dimensional functions represent the 95% confidence interval.

Fig. S38: Model for the 2010 October intrusion

December 2010 eruption

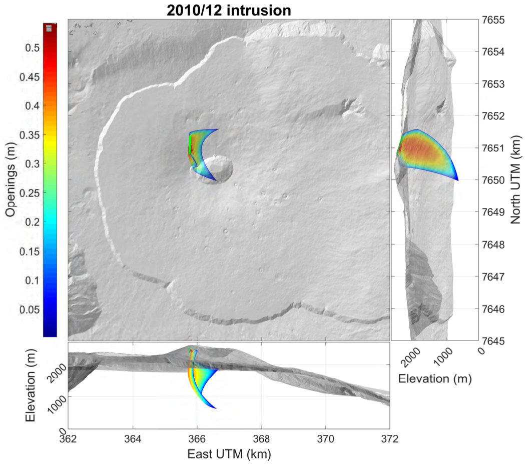

(a) 3D best geometry. Eruptive fissures are indicated by green lines.

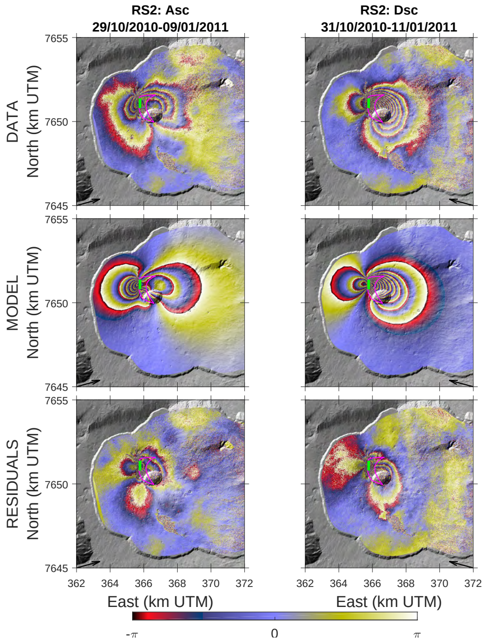

(b) Observed, modeled and residual displacements on wrapped data. Best model contour is in magenta and eruptive fissures are indicated by green lines. Line of sight of satellites acquisition is indicated by arrows.

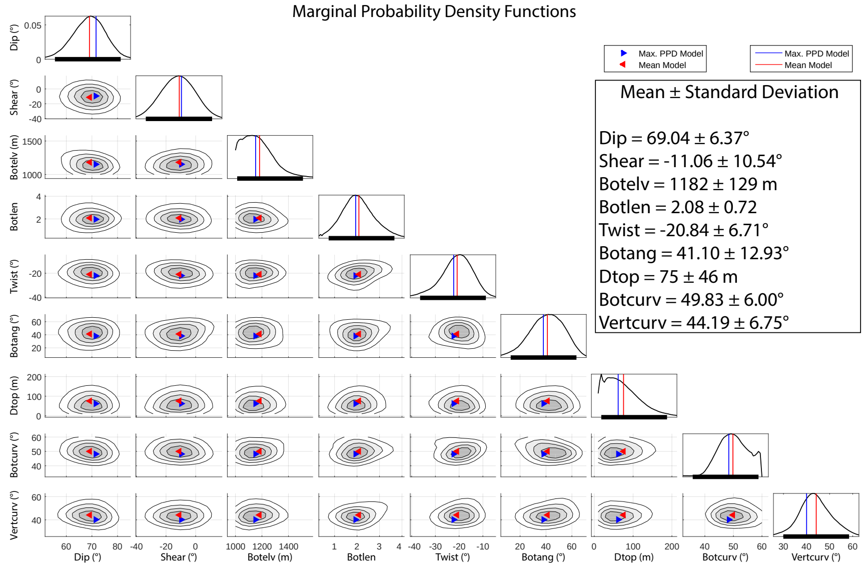

(c) Marginal posterior probability density functions. One-dimensional and two-dimensional functions are given in the diagonal and off-diagonal, respectively. Maximum and mean values are indicated by blue and red triangles, respectively. Black thick lines on one-dimensional functions represent the 95% confidence interval.

Fig. S39: Model for the 2010 December intrusion

June 2014 eruption

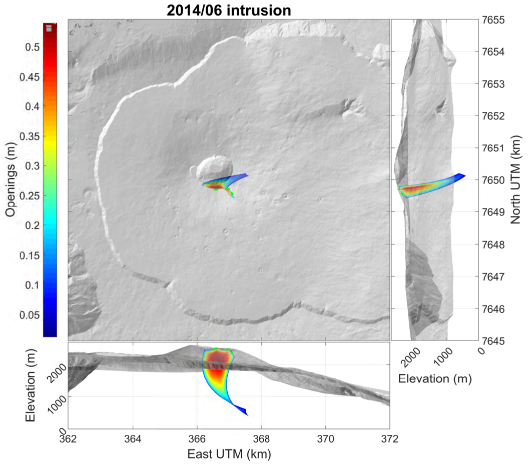

(a) 3D best geometry. Eruptive fissures are indicated by green lines.

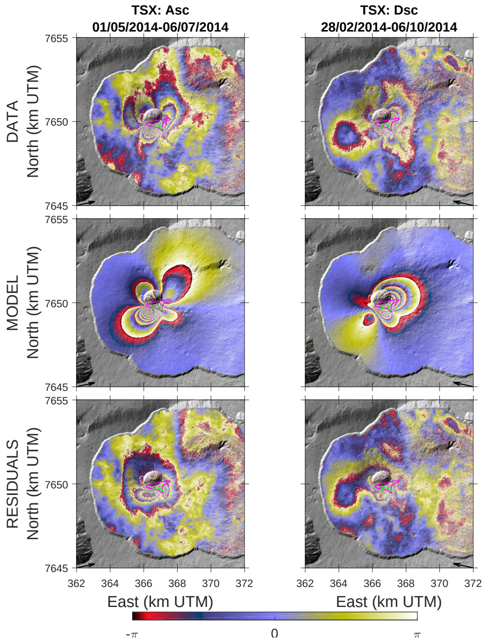

(b) Observed, modeled and residual displacements on wrapped data. Best model contour is in magenta and eruptive fissures are indicated by green lines. Line of sight of satellites acquisition is indicated by arrows.

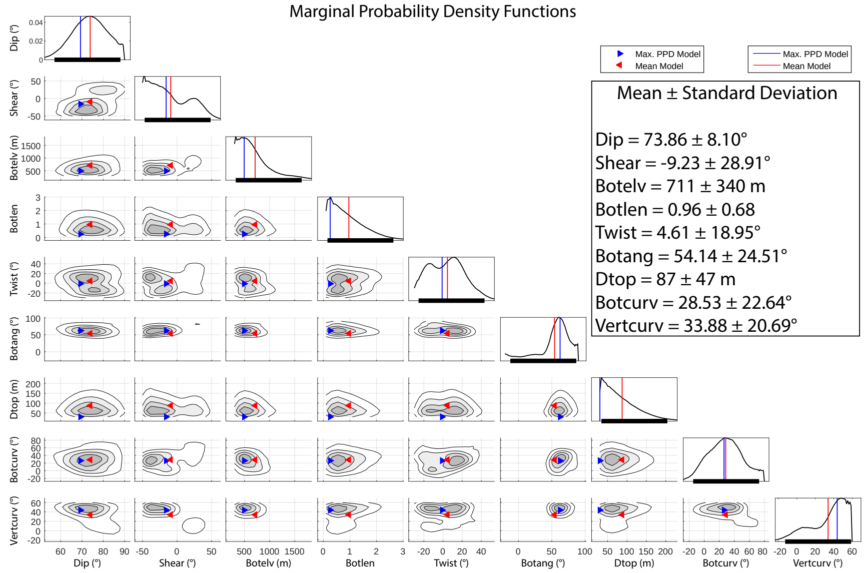

(c) Marginal posterior probability density functions. One-dimensional and two-dimensional functions are given in the diagonal and off-diagonal, respectively. Maximum and mean values are indicated by blue and red triangles, respectively. Black thick lines on one-dimensional functions represent the 95% confidence interval.

Fig. S40: Model for the 2014 June intrusion

February 2015 eruption

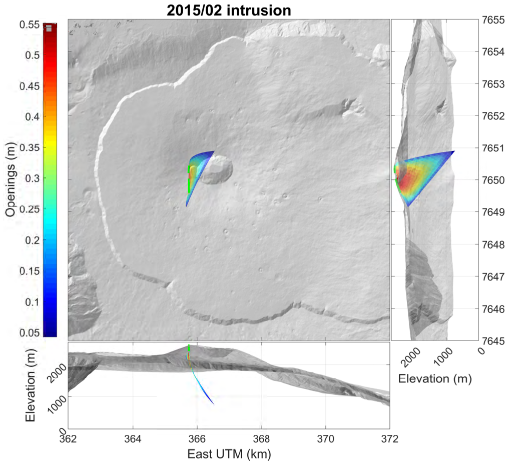

(a) 3D best geometry. Eruptive fissures are indicated by green lines.

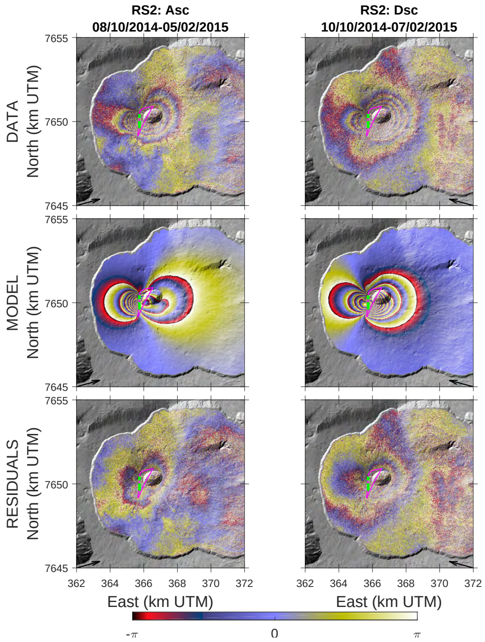

(b) Observed, modeled and residual displacements on wrapped data. Best model contour is in magenta and eruptive fissures are indicated by green lines. Line of sight of satellites acquisition is indicated by arrows.

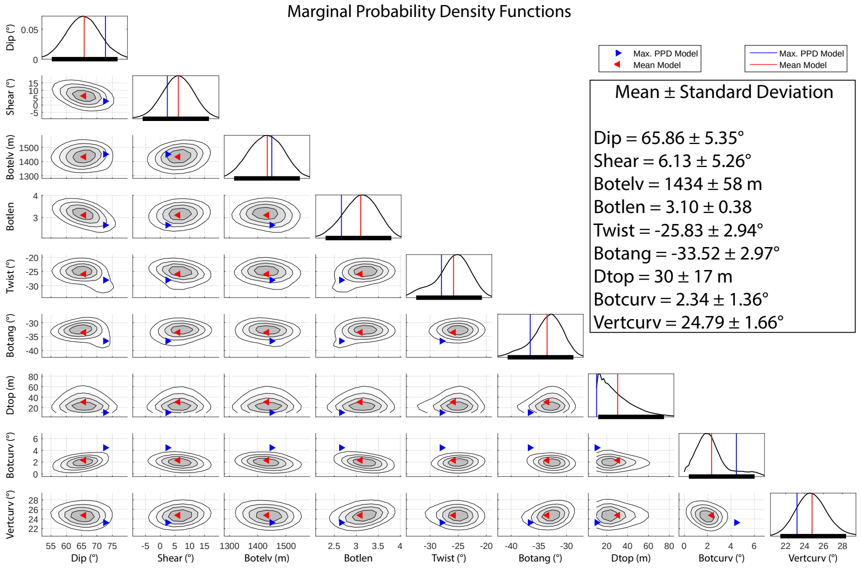

(c) Marginal posterior probability density functions. One-dimensional and two-dimensional functions are given in the diagonal and off-diagonal, respectively. Maximum and mean values are indicated by blue and red triangles, respectively. Black thick lines on one-dimensional functions represent the 95% confidence interval.

Fig. S41: Model for the 2015 February intrusion

May 2015 eruption

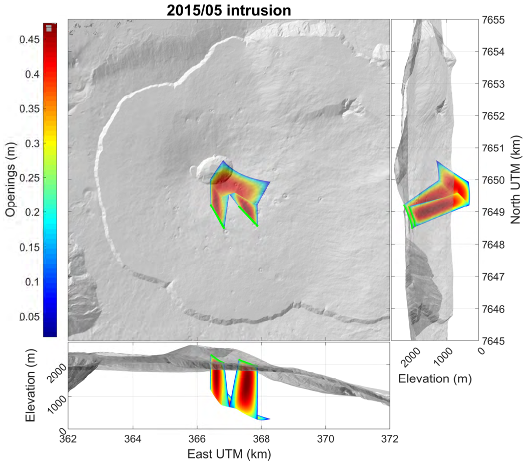

(a) 3D best geometry. Eruptive fissures are indicated by green lines.

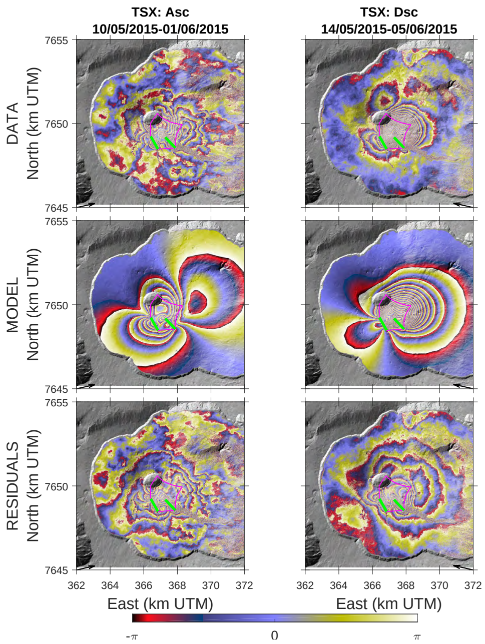

(b) Observed, modeled and residual displacements on wrapped data. Best model contour is in magenta and eruptive fissures are indicated by green lines. Line of sight of satellites acquisition is indicated by arrows.

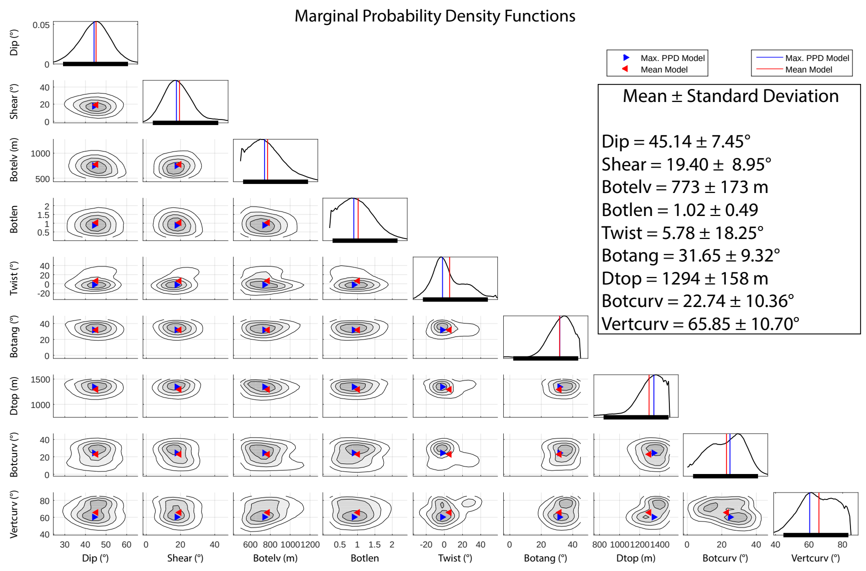

(c) Marginal posterior probability density functions. One-dimensional and two-dimensional functions are given in the diagonal and off-diagonal, respectively. Maximum and mean values are indicated by blue and red triangles, respectively. Black thick lines on one-dimensional functions represent the 95% confidence interval.

Fig. S42: Model for the 2015 May intrusion

July 2015 eruption

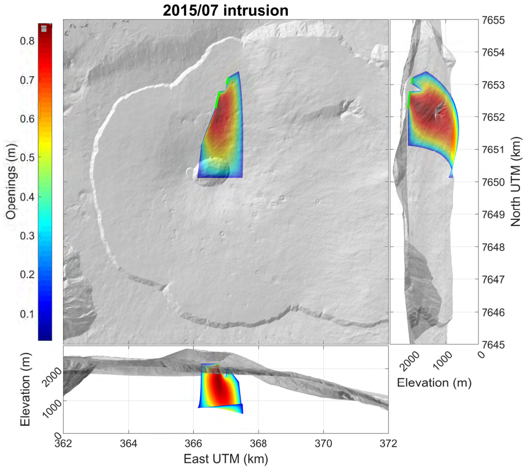

(**a**) 3D best geometry. Eruptive fissures are indicated by green lines.

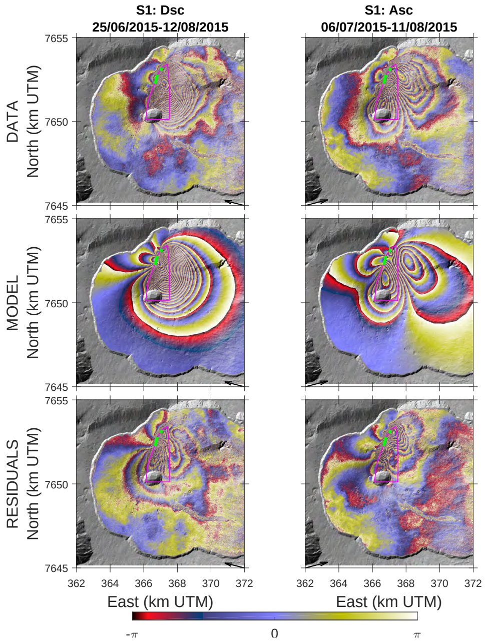

(**b**) Observed, modeled and residual displacements on wrapped data. Best model contour is in magenta and eruptive fissures are indicated by green lines. Line of sight of satellites acquisition is indicated by arrows.

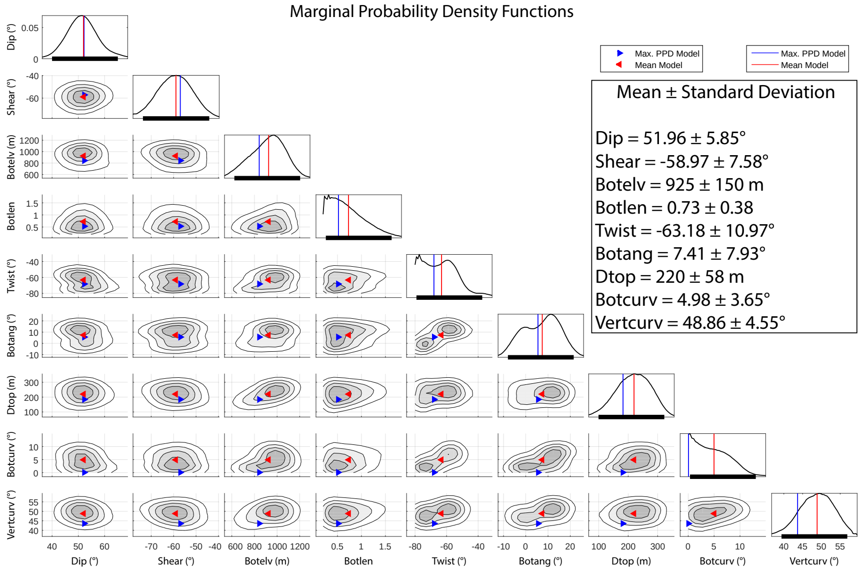

(**c**) Marginal posterior probability density functions. One-dimensional and two-dimensional functions are given in the diagonal and off-diagonal, respectively. Maximum and mean values are indicated by blue and red triangles, respectively. Black thick lines on one-dimensional functions represent the 95% confidence interval.

Fig. S43: Model for the 2015 July intrusion

August 2015 eruption

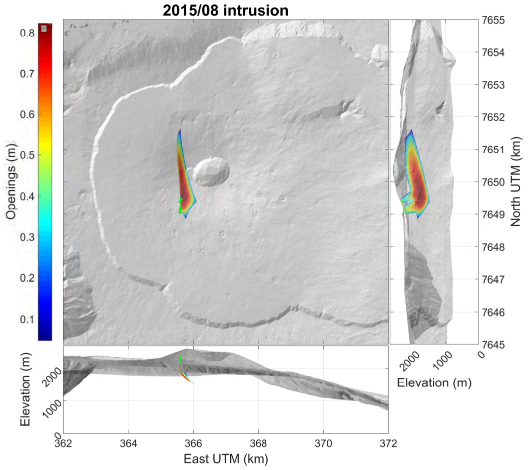

(a) 3D best geometry. Eruptive fissures are indicated by green lines.

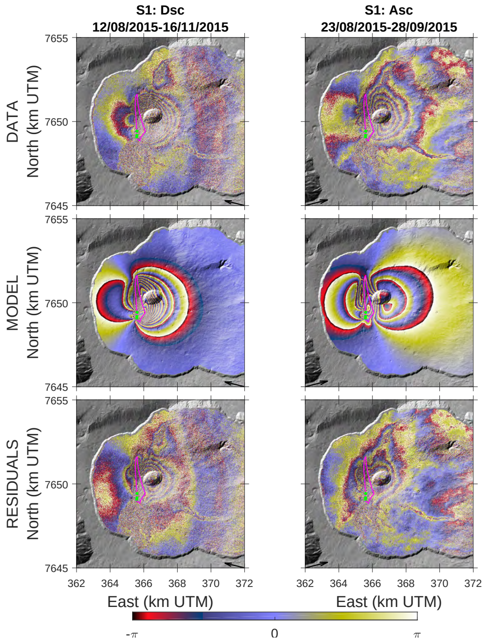

(b) Observed, modeled and residual displacements on wrapped data. Best model contour is in magenta and eruptive fissures are indicated by green lines. Line of sight of satellites acquisition is indicated by arrows.

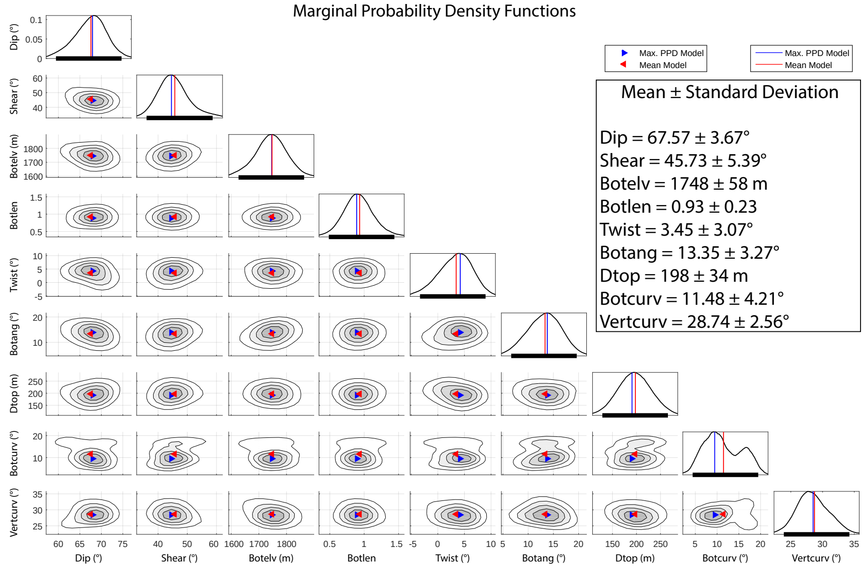

(c) Marginal posterior probability density functions. One-dimensional and two-dimensional functions are given in the diagonal and off-diagonal, respectively. Maximum and mean values are indicated by blue and red triangles, respectively. Black thick lines on one-dimensional functions represent the 95% confidence interval.

Fig. S44: Model for the 2015 August intrusion

May 2016 eruption, model from Smittarello et al. [5]

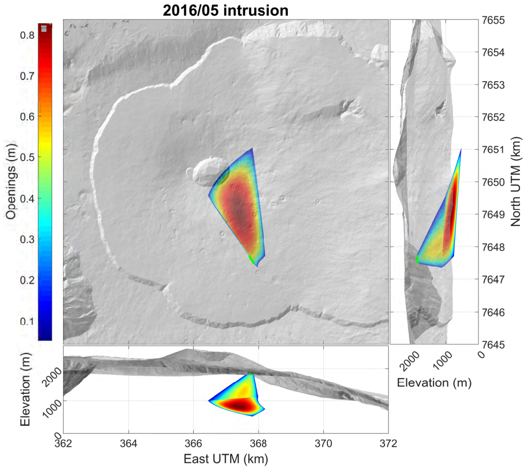

(a) 3D best geometry. Eruptive fissures are indicated by green lines.

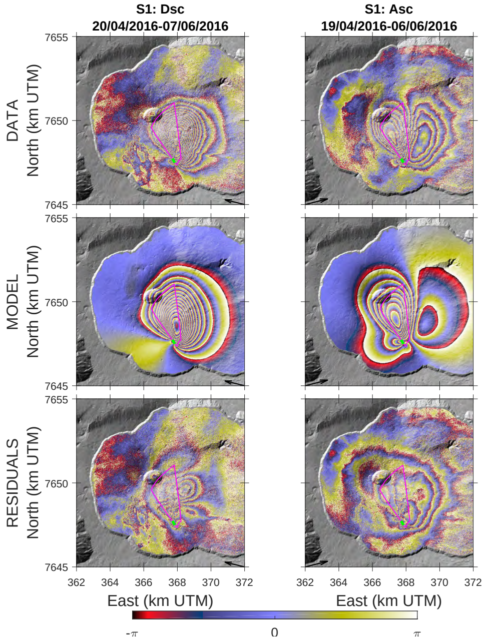

(b) Observed, modeled and residual displacements on wrapped data. Best model contour is in magenta and eruptive fissures are indicated by green lines. Line of sight of satellites acquisition is indicated by arrows.

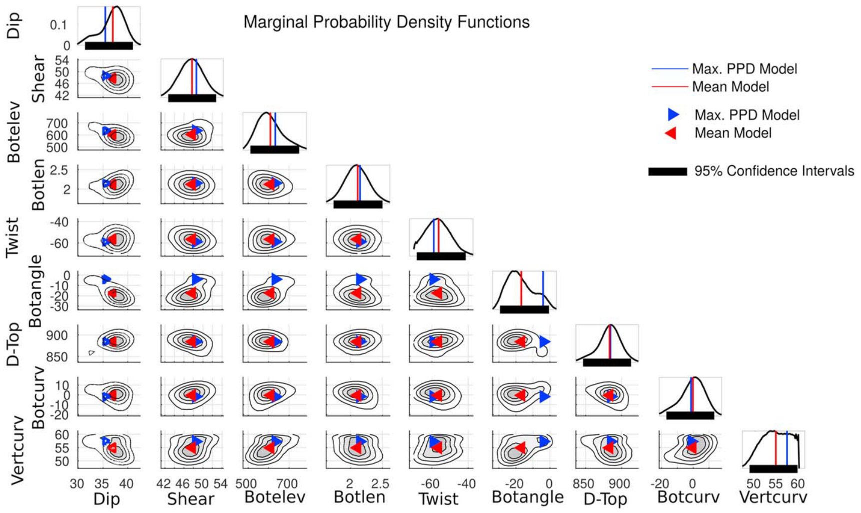

(c) Marginal posterior probability density functions. One-dimensional and two-dimensional functions are given in the diagonal and off-diagonal, respectively. Maximum and mean values are indicated by blue and red triangles, respectively. Black thick lines on one-dimensional functions represent the 95% confidence interval. Figure from [5].

Fig. S45: Model for the 2016 September intrusion

September 2016 eruption

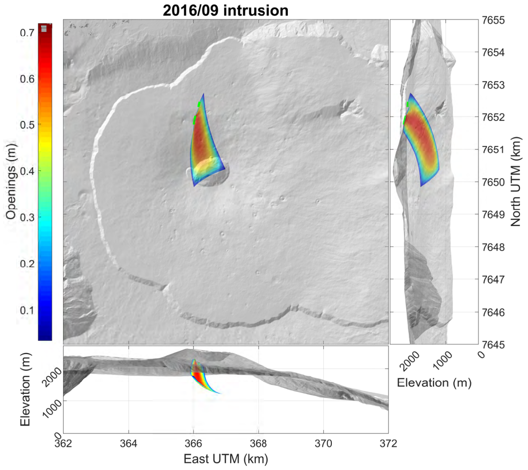

(a) 3D best geometry. Eruptive fissures are indicated by green lines.

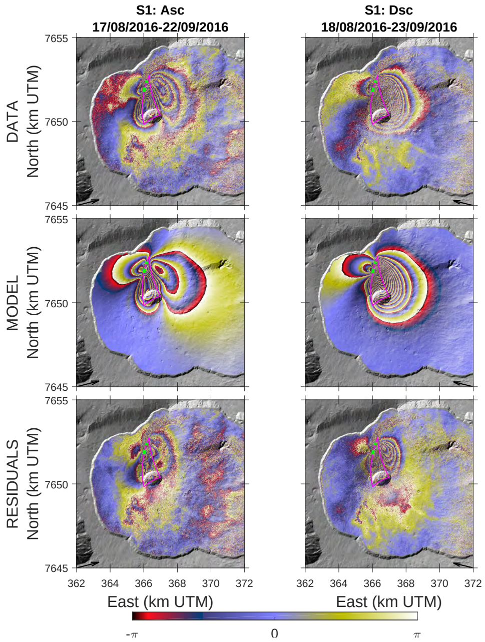

(b) Observed, modeled and residual displacements on wrapped data. Best model contour is in magenta and eruptive fissures are indicated by green lines. Line of sight of satellites acquisition is indicated by arrows.

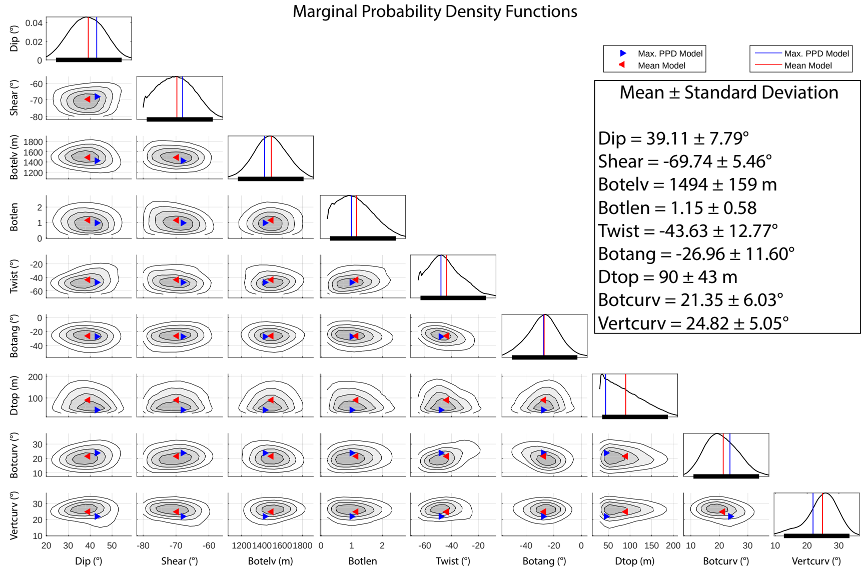

(c) Marginal posterior probability density functions. One-dimensional and two-dimensional functions are given in the diagonal and off-diagonal, respectively. Maximum and mean values are indicated by blue and red triangles, respectively. Black thick lines on one-dimensional functions represent the 95% confidence interval.

Fig. S46: Model for the 2016 September intrusion

January 2017 eruption

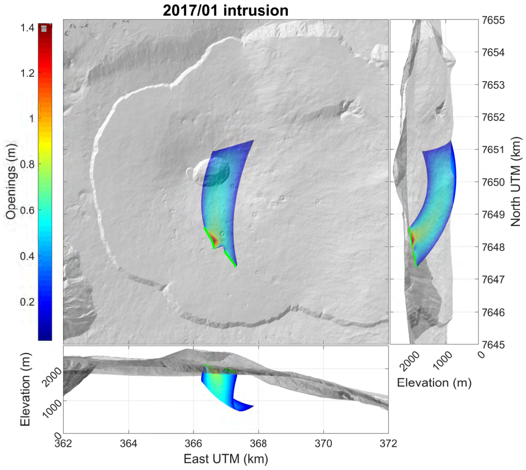

(a) 3D best geometry. Eruptive fissures are indicated by green lines.

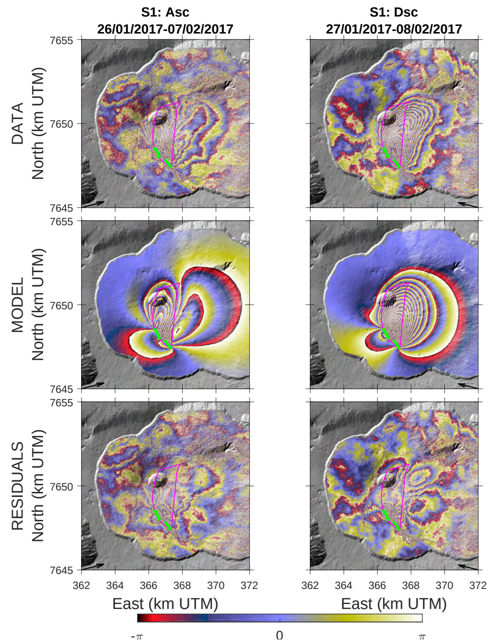

(b) Observed, modeled and residual displacements on wrapped data. Best model contour is in magenta and eruptive fissures are indicated by green lines. Line of sight of satellites acquisition is indicated by arrows.

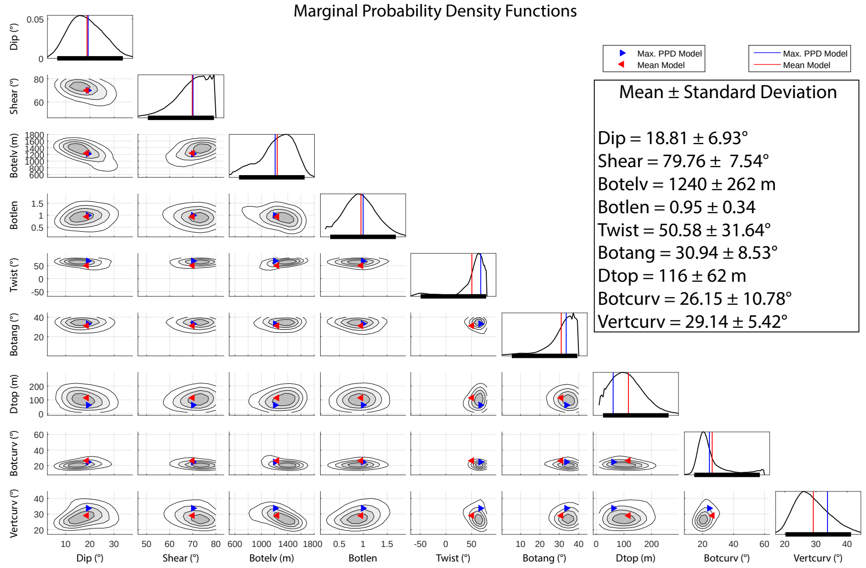

(c) Marginal posterior probability density functions. One-dimensional and two-dimensional functions are given in the diagonal and off-diagonal, respectively. Maximum and mean values are indicated by blue and red triangles, respectively. Black thick lines on one-dimensional functions represent the 95% confidence interval.

Fig. S47: Model for the 2017 January intrusion

May 2017 intrusion

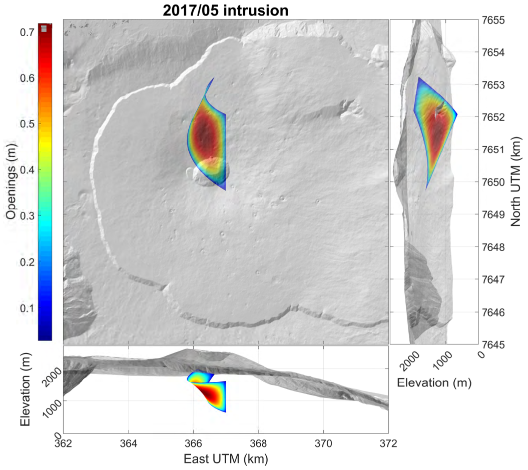

(**a**) 3D best geometry. Eruptive fissures are indicated by green lines.

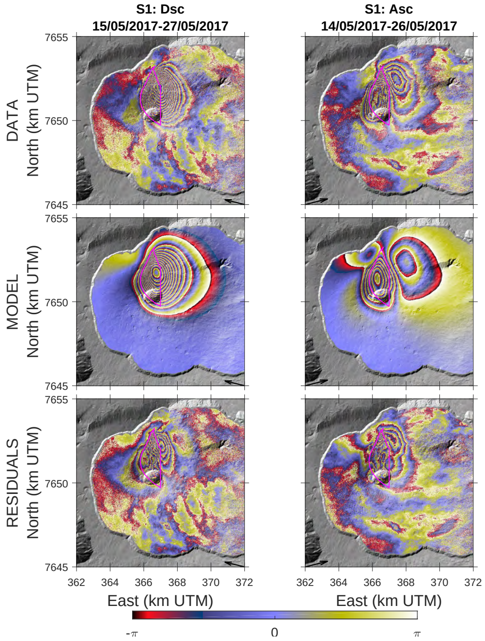

(**b**) Observed, modeled and residual displacements on wrapped data. Best model contour is in magenta and eruptive fissures are indicated by green lines. Line of sight of satellites acquisition is indicated by arrows.

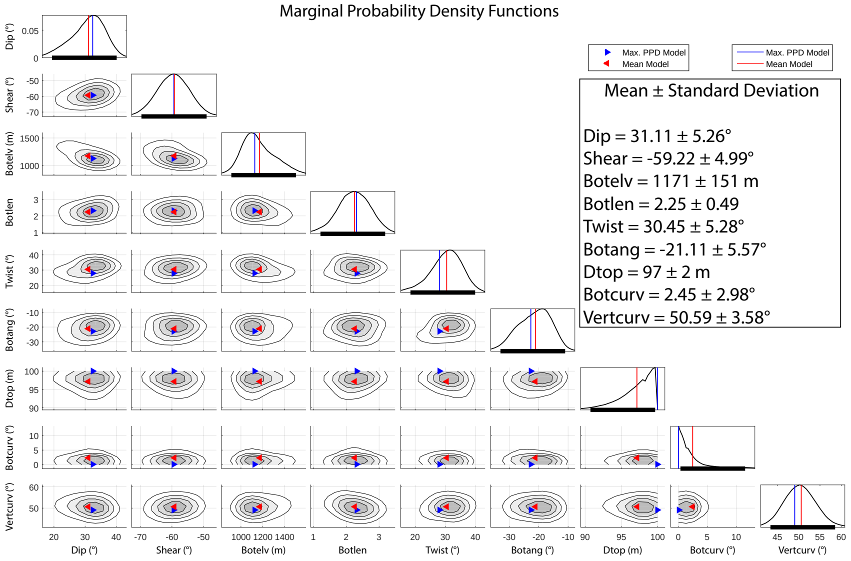

(**c**) Marginal posterior probability density functions. One-dimensional and two-dimensional functions are given in the diagonal and off-diagonal, respectively. Maximum and mean values are indicated by blue and red triangles, respectively. Black thick lines on one-dimensional functions represent the 95% confidence interval.

Fig. S48: Model for the 2017 May intrusion

July 2017 eruption, model from Dumont et al. [6]

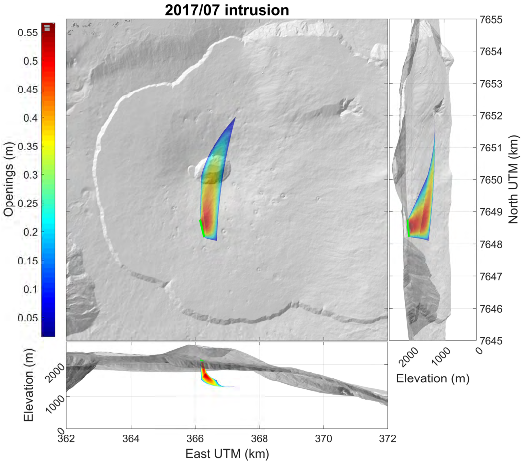

(a) 3D best geometry. Eruptive fissures are indicated by green lines.

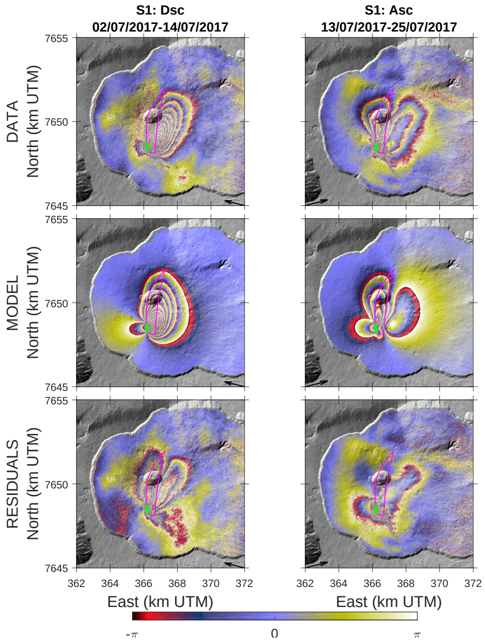

(b) Observed, modeled and residual displacements on wrapped data. Best model contour is in magenta and eruptive fissures are indicated by green lines. Line of sight of satellites acquisition is indicated by arrows.

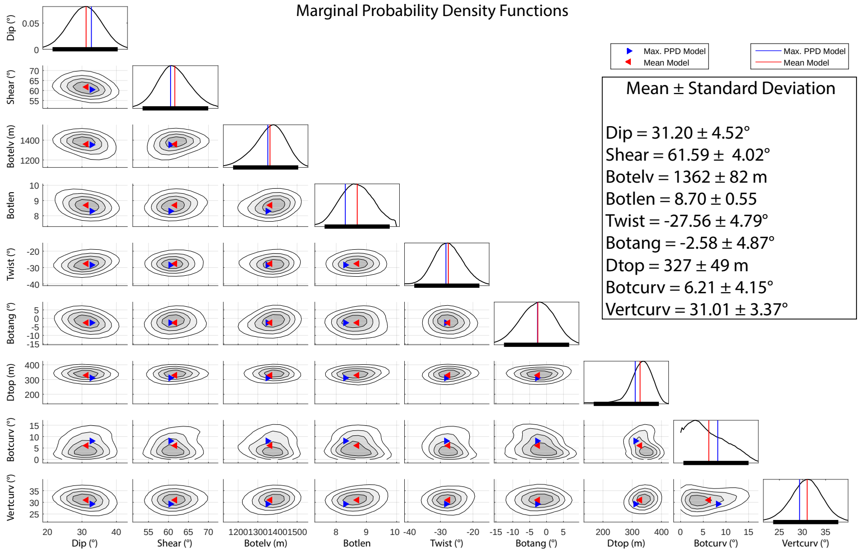

(c) Marginal posterior probability density functions. One-dimensional and two-dimensional functions are given in the diagonal and off-diagonal, respectively. Maximum and mean values are indicated by blue and red triangles, respectively. Black thick lines on one-dimensional functions represent the 95% confidence interval.

Fig. S49: Model for the 2017 July intrusion

3 April 2018 eruption

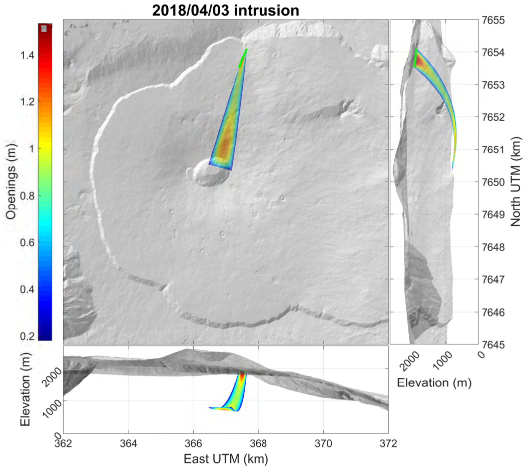

(a) 3D best geometry. Eruptive fissures are indicated by green lines.

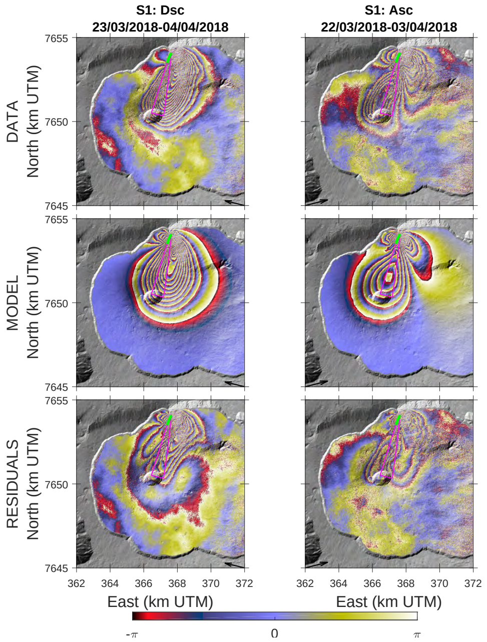

(b) Observed, modeled and residual displacements on wrapped data. Best model contour is in magenta and eruptive fissures are indicated by green lines. Line of sight of satellites acquisition is indicated by arrows.

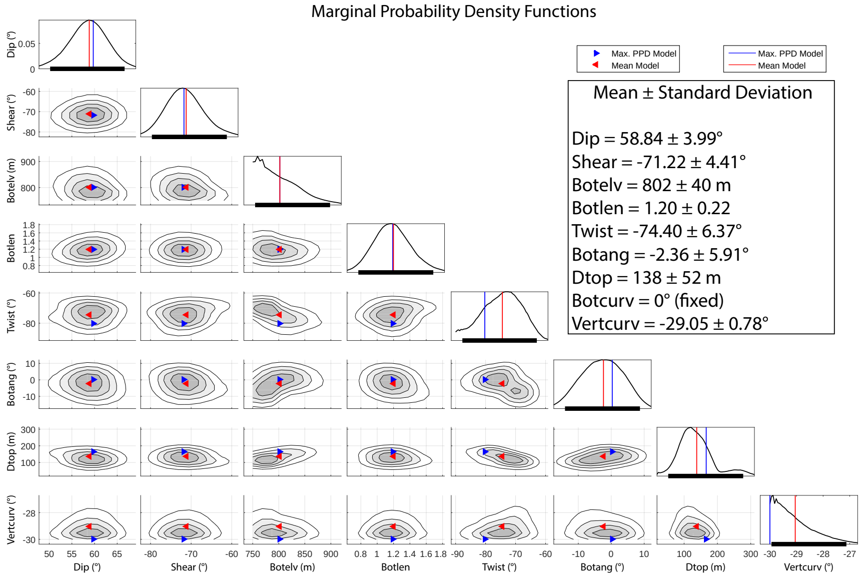

(c) Marginal posterior probability density functions. One-dimensional and two-dimensional functions are given in the diagonal and off-diagonal, respectively. Maximum and mean values are indicated by blue and red triangles, respectively. Black thick lines on one-dimensional functions represent the 95% confidence interval.

Fig. S50: Model for the 2018 April 3rd intrusion

27 April 2018 eruption

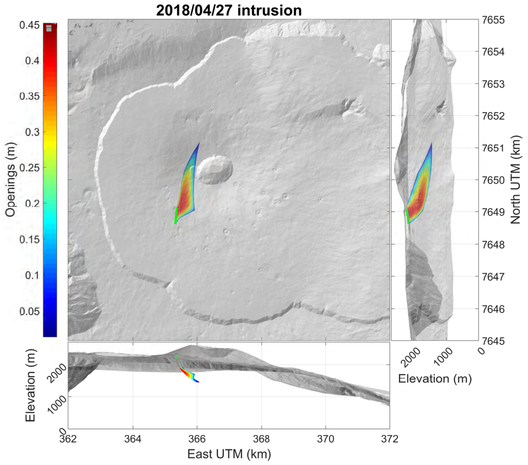

(a) 3D best geometry. Eruptive fissures are indicated by green lines.

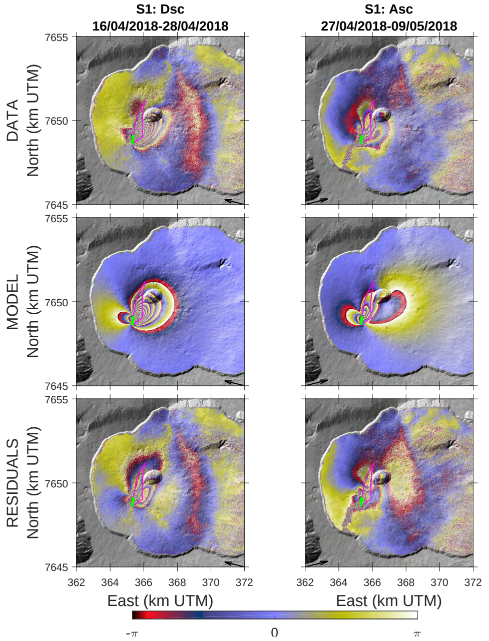

(b) Observed, modeled and residual displacements on wrapped data. Best model contour is in magenta and eruptive fissures are indicated by green lines. Line of sight of satellites acquisition is indicated by arrows.

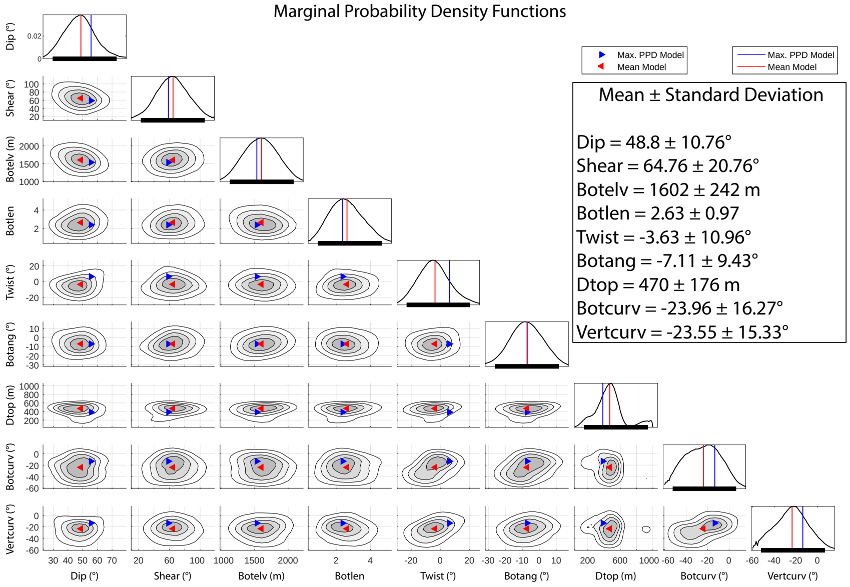

(c) Marginal posterior probability density functions. One-dimensional and two-dimensional functions are given in the diagonal and off-diagonal, respectively. Maximum and mean values are indicated by blue and red triangles, respectively. Black thick lines on one-dimensional functions represent the 95% confidence interval.

Fig. S51: Model for the 2018 April 27th intrusion

July 2018 eruption

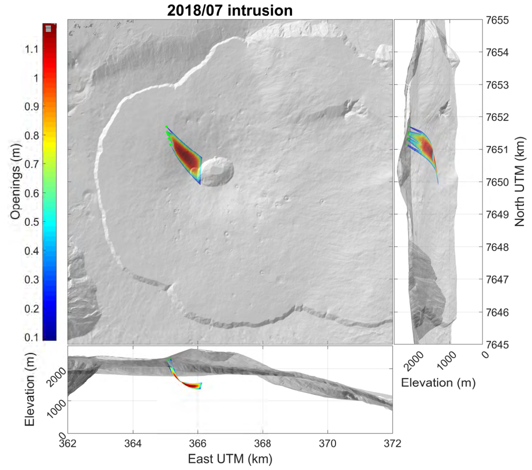

(a) 3D best geometry. Eruptive fissures are indicated by green lines.

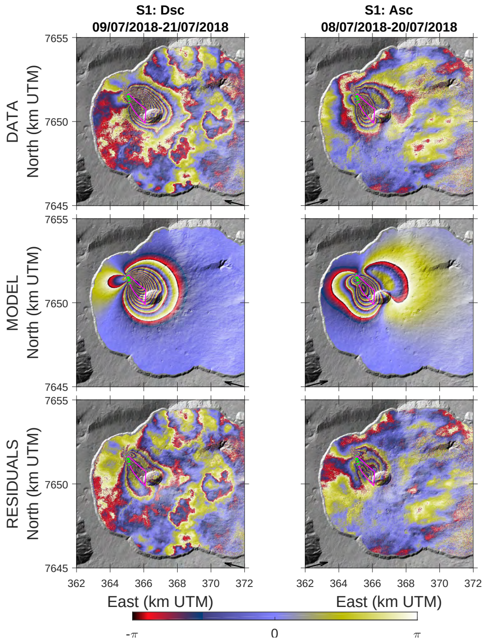

(b) Observed, modeled and residual displacements on wrapped data. Best model contour is in magenta and eruptive fissures are indicated by green lines. Line of sight of satellites acquisition is indicated by arrows.

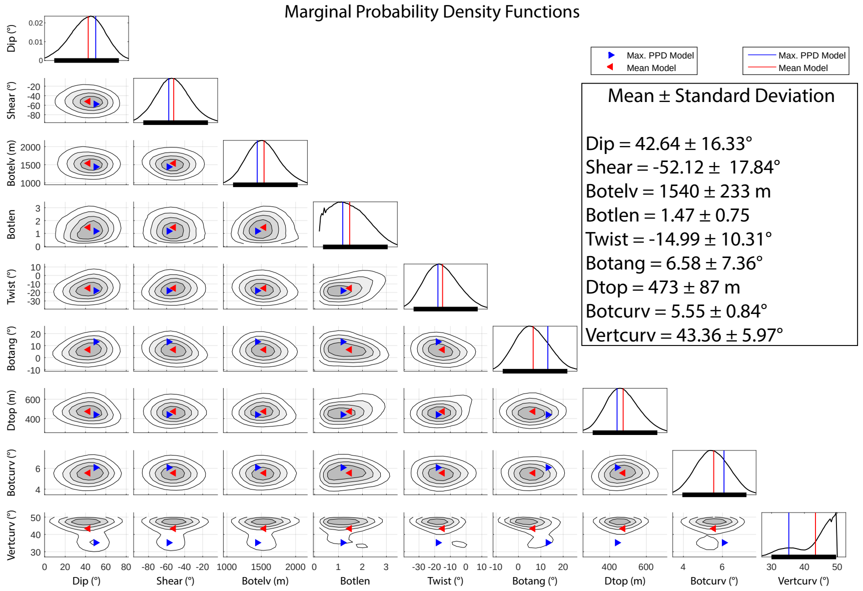

(c) Marginal posterior probability density functions. One-dimensional and two-dimensional functions are given in the diagonal and off-diagonal, respectively. Maximum and mean values are indicated by blue and red triangles, respectively. Black thick lines on one-dimensional functions represent the 95% confidence interval.

Fig. S52: Model for the 2018 July intrusion

September 2018 eruption

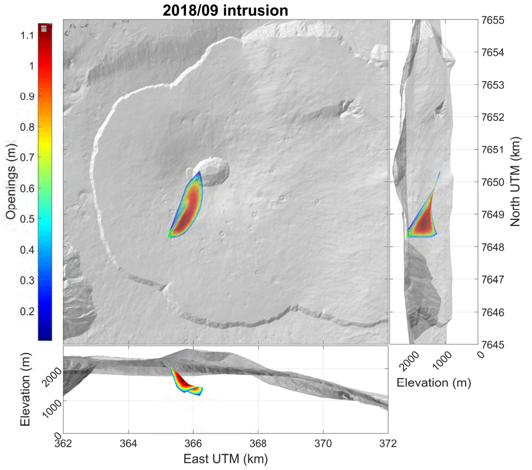

(a) 3D best geometry. Eruptive fissures are indicated by green lines.

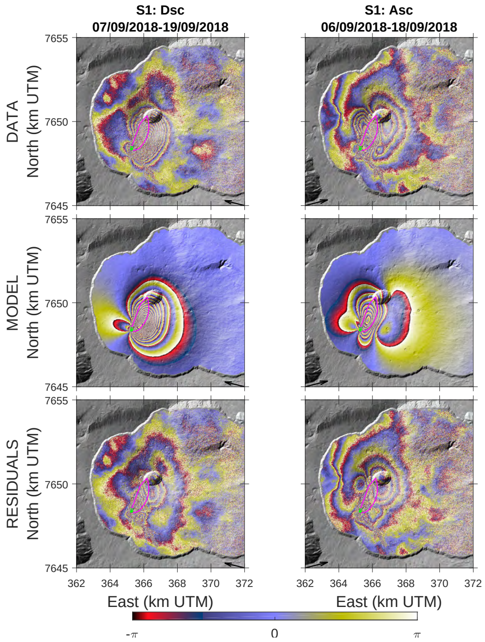

(b) Observed, modeled and residual displacements on wrapped data. Best model contour is in magenta and eruptive fissures are indicated by green lines. Line of sight of satellites acquisition is indicated by arrows.

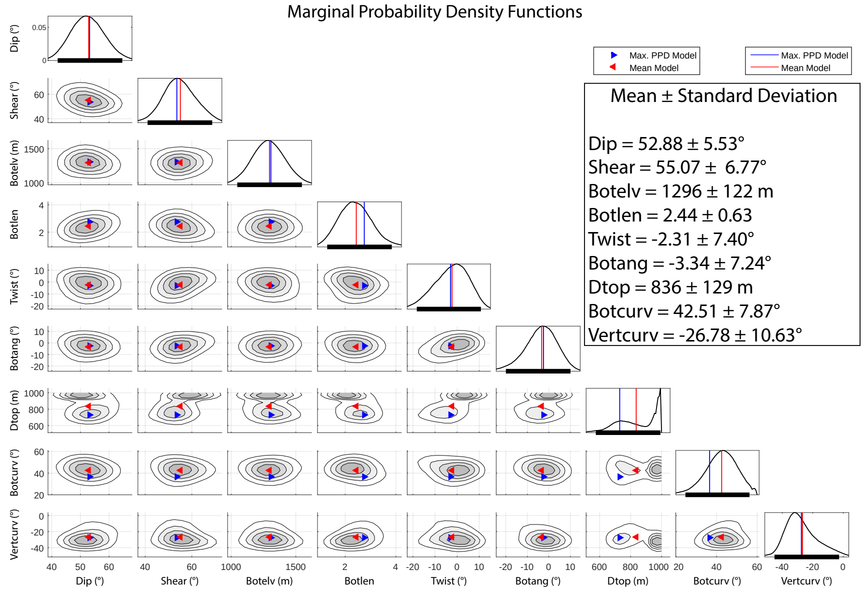

(c) Marginal posterior probability density functions. One-dimensional and two-dimensional functions are given in the diagonal and off-diagonal, respectively. Maximum and mean values are indicated by blue and red triangles, respectively. Black thick lines on one-dimensional functions represent the 95% confidence interval.

Fig. S53: Model for the 2018 September intrusion

February 2019 eruption

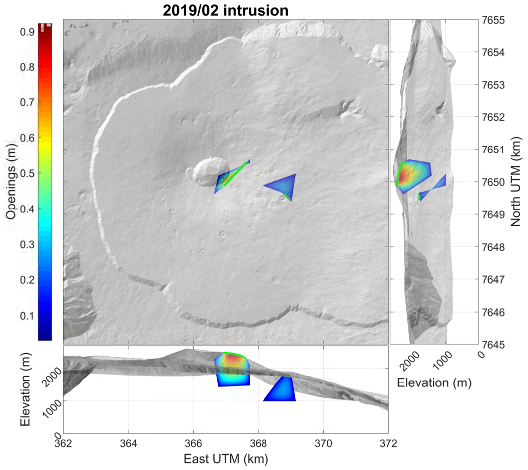

(**a**) 3D best geometry. Eruptive fissures are indicated by green lines.

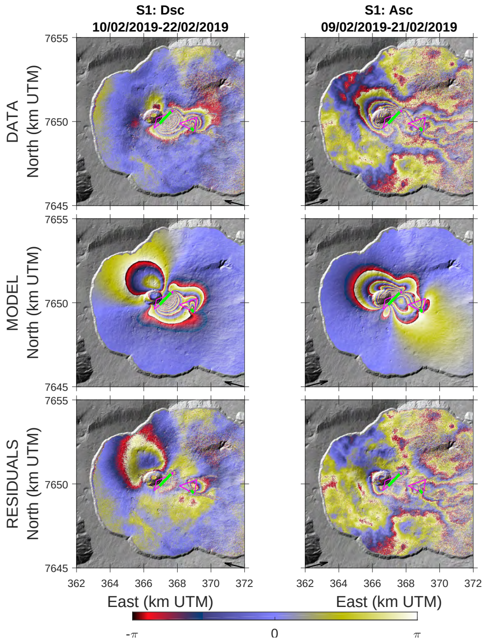

(**b**) Observed, modeled and residual displacements on wrapped data. Best model contour is in magenta and eruptive fissures are indicated by green lines. Line of sight of satellites acquisition is indicated by arrows.

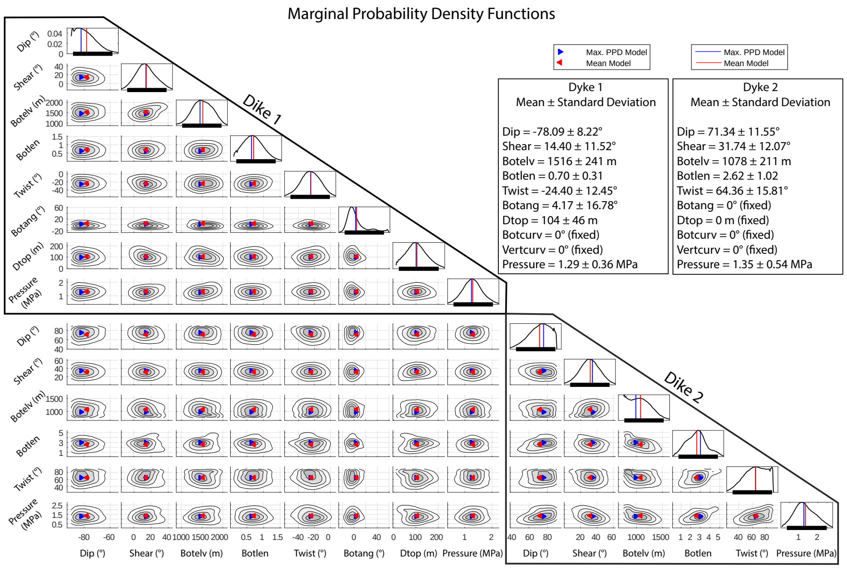

(**c**) Marginal posterior probability density functions. One-dimensional and two-dimensional functions are given in the diagonal and off-diagonal, respectively. Maximum and mean values are indicated by blue and red triangles, respectively. Black thick lines on one-dimensional functions represent the 95% confidence interval.

Fig. S54: Model for the 2019 February intrusion

June 2019 eruption

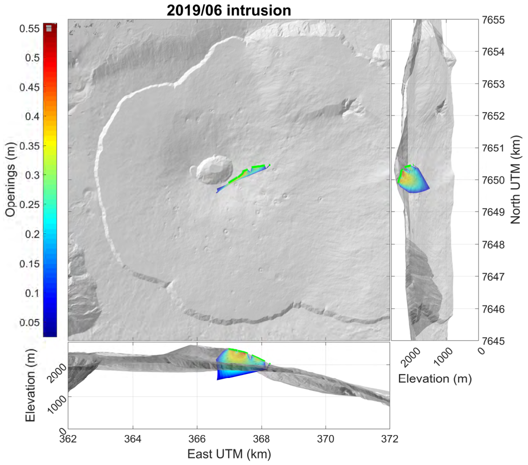

(a) 3D best geometry. Eruptive fissures are indicated by green lines.

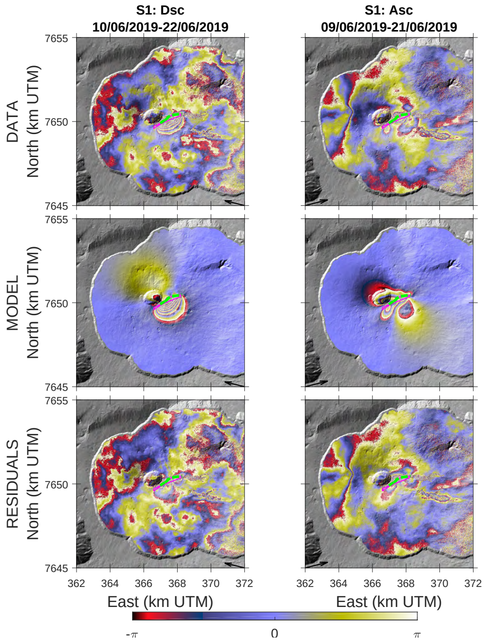

(b) Observed, modeled and residual displacements on wrapped data. Best model contour is in magenta and eruptive fissures are indicated by green lines. Line of sight of satellites acquisition is indicated by arrows.

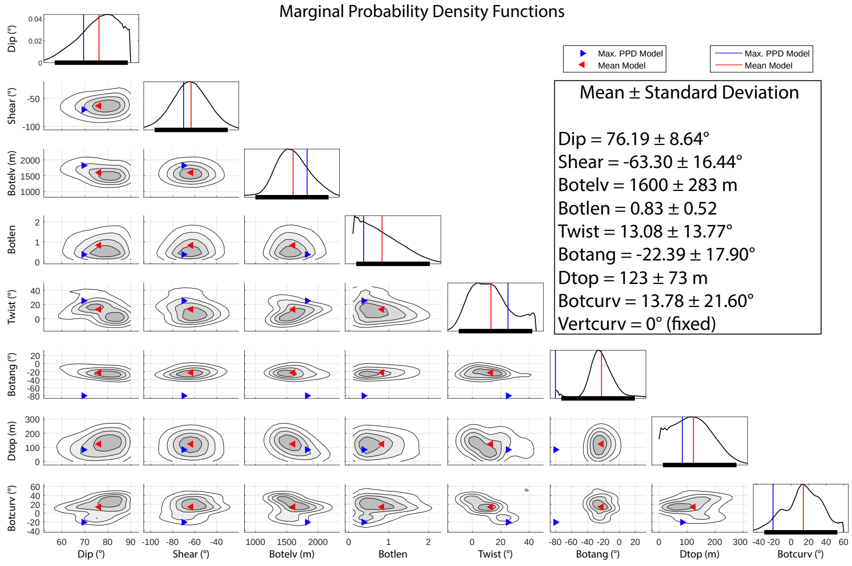

(c) Marginal posterior probability density functions. One-dimensional and two-dimensional functions are given in the diagonal and off-diagonal, respectively. Maximum and mean values are indicated by blue and red triangles, respectively. Black thick lines on one-dimensional functions represent the 95% confidence interval.

Fig. S55: Model for the 2019 June intrusion

July 2019 eruption

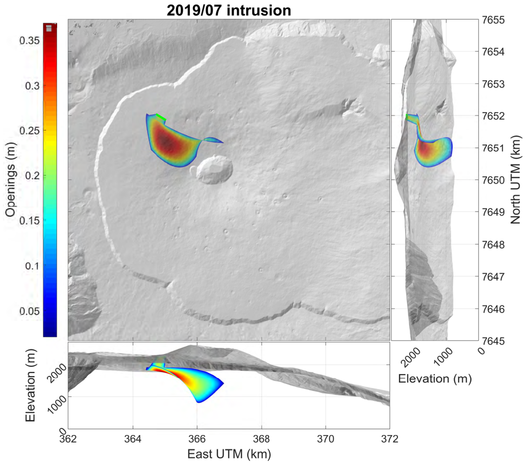

(a) 3D best geometry. Eruptive fissures are indicated by green lines.

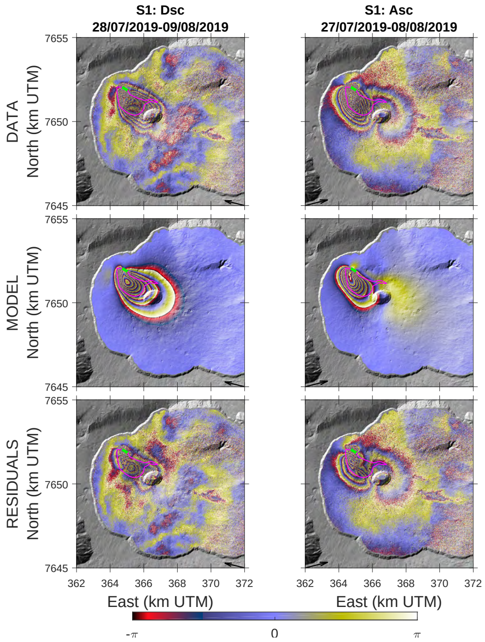

(b) Observed, modeled and residual displacements on wrapped data. Best model contour is in magenta and eruptive fissures are indicated by green lines. Line of sight of satellites acquisition is indicated by arrows.

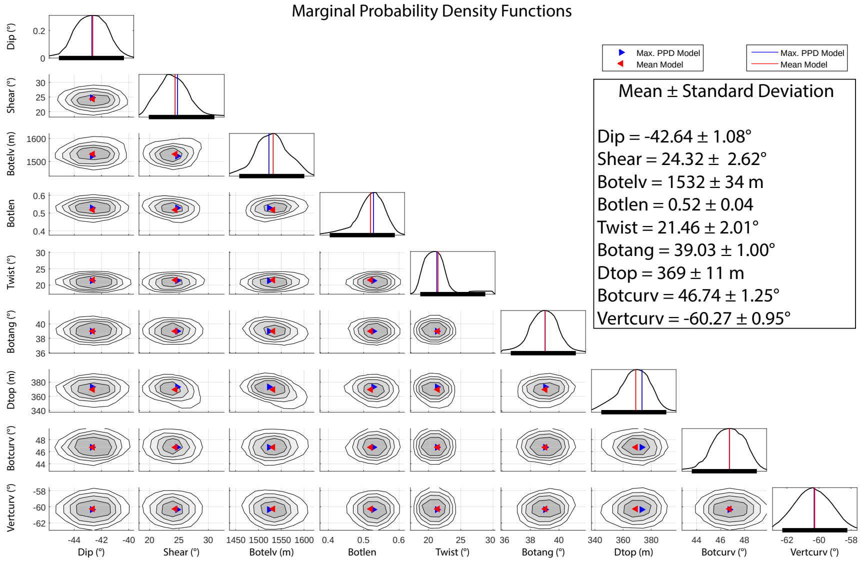

(c) Marginal posterior probability density functions. One-dimensional and two-dimensional functions are given in the diagonal and off-diagonal, respectively. Maximum and mean values are indicated by blue and red triangles, respectively. Black thick lines on one-dimensional functions represent the 95% confidence interval.

Fig. S56: Model for the 2019 July intrusion

August 2019 eruption

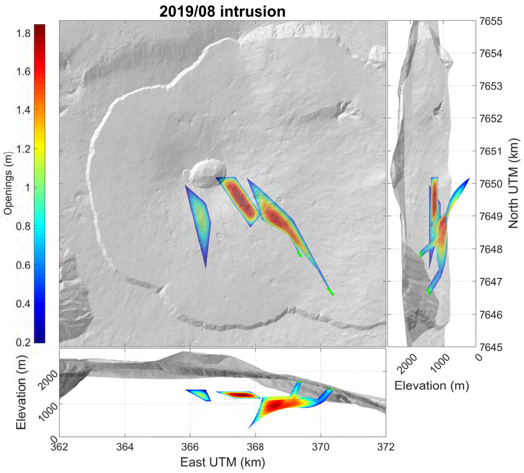

(**a**) 3D best geometry. Eruptive fissures are indicated by green lines.

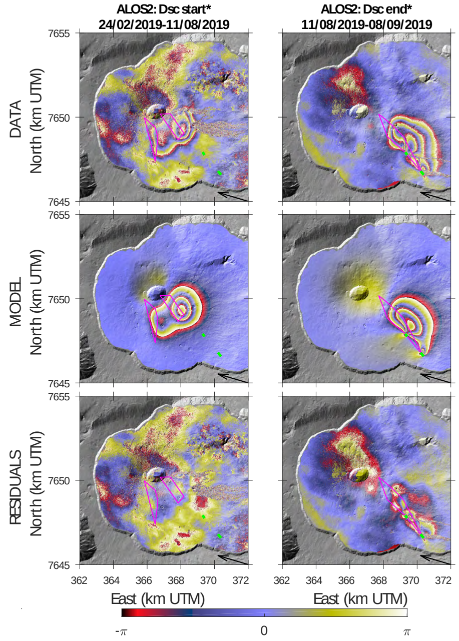

(**b**) Observed, modeled and residual displacements on wrapped data. Best model contour is in magenta and eruptive fissures are indicated by green lines. Line of sight of satellites acquisition is indicated by arrows. One inversion was carried out for the intrusion start by involving two sources and one inversion was done for the intrusion end using one source.

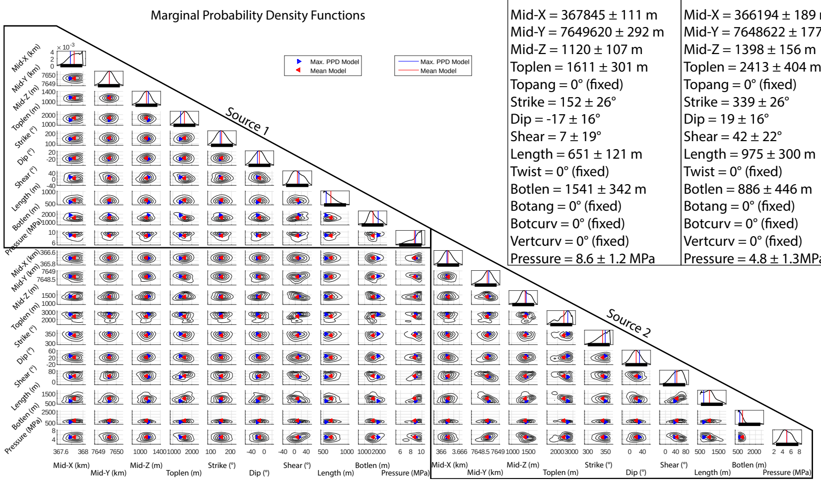

Mean ± Standard Deviation Source 1	Mean ± Standard Deviation Source 2
Mid-X = 367845 ± 111 m	Mid-X = 366194 ± 189 m
Mid-Y = 7649620 ± 292 m	Mid-Y = 7648622 ± 177 m
Mid-Z = 1120 ± 107 m	Mid-Z = 1398 ± 156 m
Toplen = 1611 ± 301 m	Toplen = 2413 ± 404 m
Topang = 0° (fixed)	Topang = 0° (fixed)
Strike = 152 ± 26°	Strike = 339 ± 26°
Dip = -17 ± 16°	Dip = 19 ± 16°
Shear = 7 ± 19°	Shear = 42 ± 22°
Length = 651 ± 121 m	Length = 975 ± 300 m
Twist = 0° (fixed)	Twist = 0° (fixed)
Botlen = 1541 ± 342 m	Botlen = 886 ± 446 m
Botang = 0° (fixed)	Botang = 0° (fixed)
Botcurv = 0° (fixed)	Botcurv = 0° (fixed)
Vertcurv = 0° (fixed)	Vertcurv = 0° (fixed)
Pressure = 8.6 ± 1.2 MPa	Pressure = 4.8 ± 1.3MPa

(**c**) Marginal posterior probability density functions for the sources of the intrusion start. One-dimensional and two-dimensional functions are given in the diagonal and off-diagonal, respectively. Maximum and mean values are indicated by blue and red triangles, respectively. Black thick lines on one-dimensional functions represent the 95% confidence interval.

Fig. S57: Model for the 2019 August intrusion

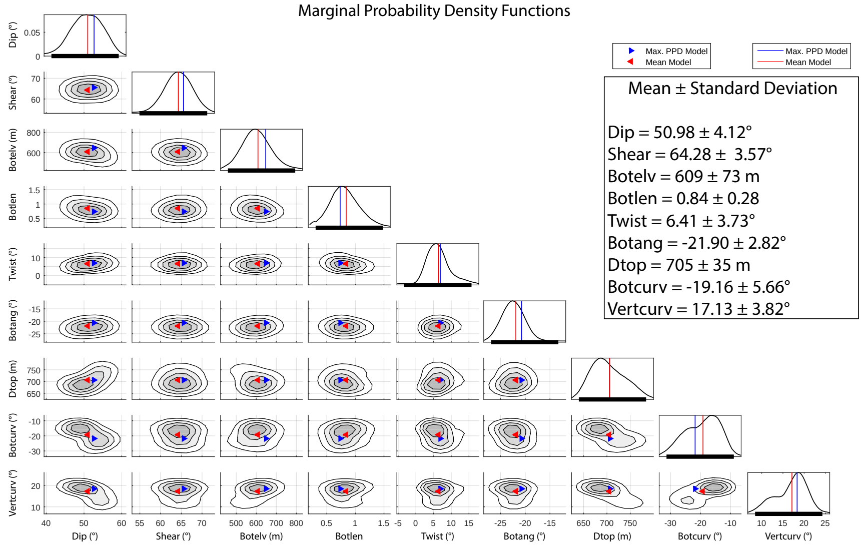

(d) Marginal posterior probability density functions for the source of the intrusion end. One-dimensional and two-dimensional functions are given in the diagonal and off-diagonal, respectively. Maximum and mean values are indicated by blue and red triangles, respectively. Black thick lines on one-dimensional functions represent the 95% confidence interval.

Fig. S57 (Continued): Model for the 2019 August intrusion

October 2019 eruption

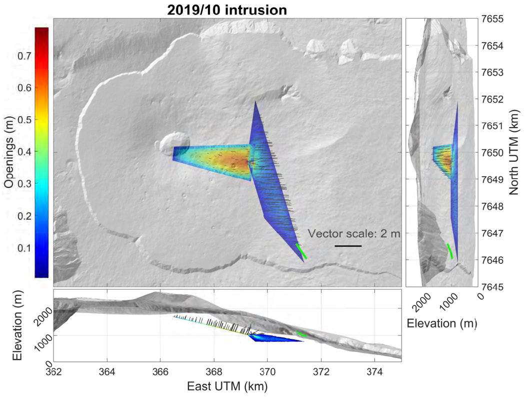

(a) 3D best geometry. Eruptive fissures are indicated by green lines.

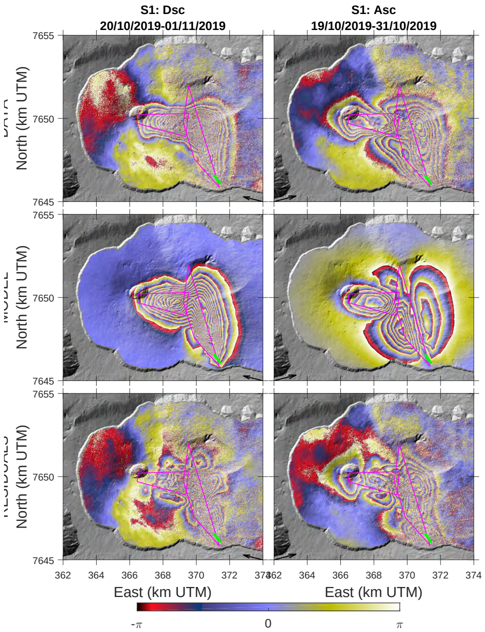

(b) Observed, modeled and residual displacements on wrapped data. Best model contour is in magenta and eruptive fissures are indicated by green lines. Line of sight of satellites acquisition is indicated by arrows.

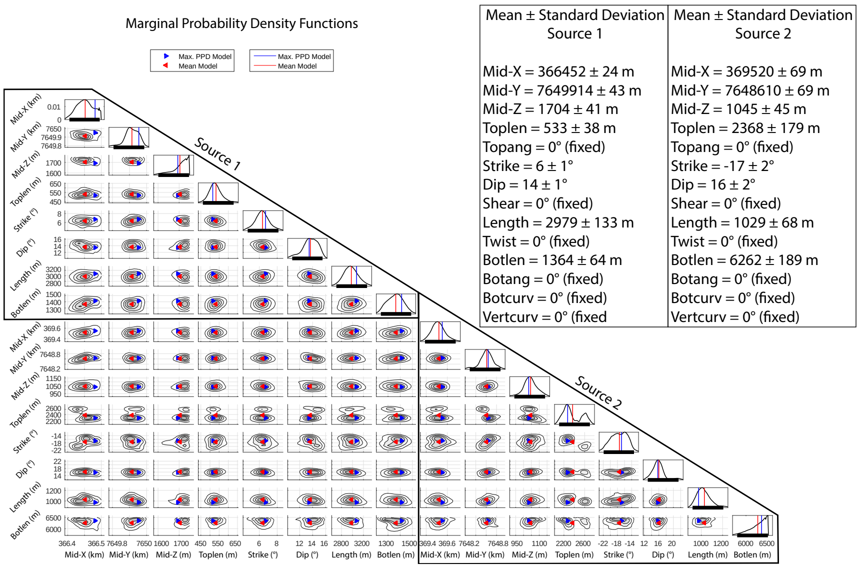

Mean ± Standard Deviation Source 1	Mean ± Standard Deviation Source 2
Mid-X = 366452 ± 24 m	Mid-X = 369520 ± 69 m
Mid-Y = 7649914 ± 43 m	Mid-Y = 7648610 ± 69 m
Mid-Z = 1704 ± 41 m	Mid-Z = 1045 ± 45 m
Toplen = 533 ± 38 m	Toplen = 2368 ± 179 m
Topang = 0° (fixed)	Topang = 0° (fixed)
Strike = 6 ± 1°	Strike = -17 ± 2°
Dip = 14 ± 1°	Dip = 16 ± 2°
Shear = 0° (fixed)	Shear = 0° (fixed)
Length = 2979 ± 133 m	Length = 1029 ± 68 m
Twist = 0° (fixed)	Twist = 0° (fixed)
Botlen = 1364 ± 64 m	Botlen = 6262 ± 189 m
Botang = 0° (fixed)	Botang = 0° (fixed)
Botcurv = 0° (fixed)	Botcurv = 0° (fixed)
Vertcurv = 0° (fixed	Vertcurv = 0° (fixed)

(c) Marginal posterior probability density functions. One-dimensional and two-dimensional functions are given in the diagonal and off-diagonal, respectively. Maximum and mean values are indicated by blue and red triangles, respectively. Black thick lines on one-dimensional functions represent the 95% confidence interval.

Fig. S58: Model for the 2019 October intrusion

February 2020 eruption

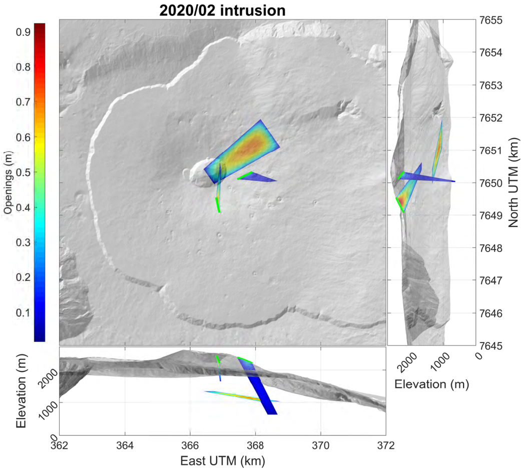

(a) 3D best geometry. Eruptive fissures are indicated by green lines.

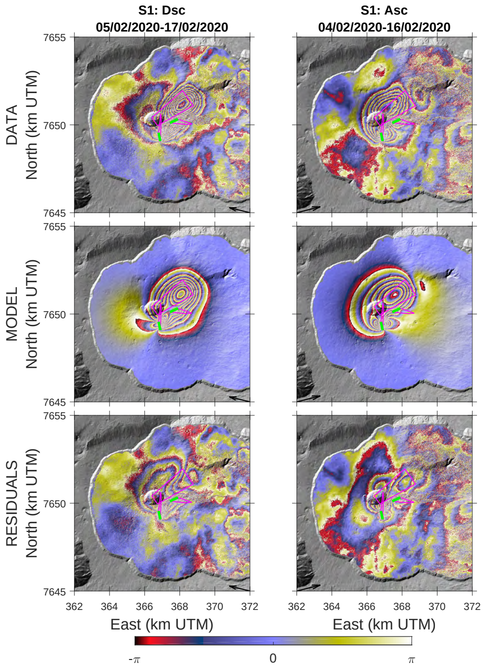

(b) Observed, modeled and residual displacements on wrapped data. Best model contour is in magenta and eruptive fissures are indicated by green lines. Line of sight of satellites acquisition is indicated by arrows.

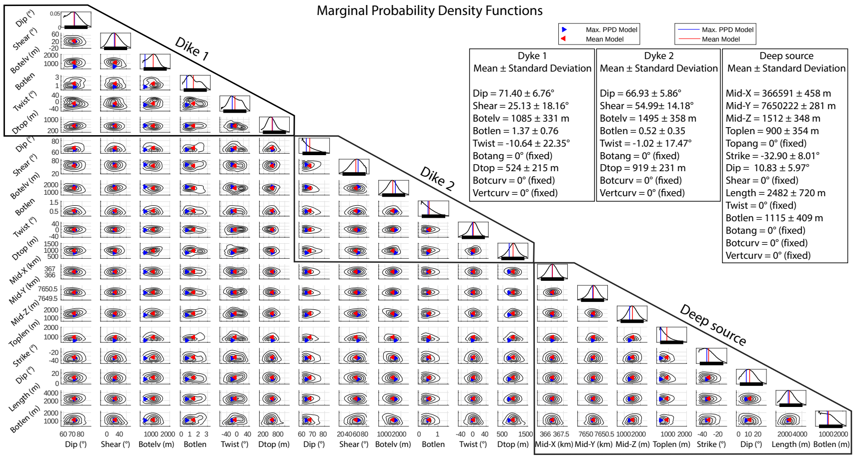

(c) Marginal posterior probability density functions. One-dimensional and two-dimensional functions are given in the diagonal and off-diagonal, respectively. Maximum and mean values are indicated by blue and red triangles, respectively. Black thick lines on one-dimensional functions represent the 95% confidence interval.

Fig. S59: Model for the 2020 February intrusion

April 2020 eruption

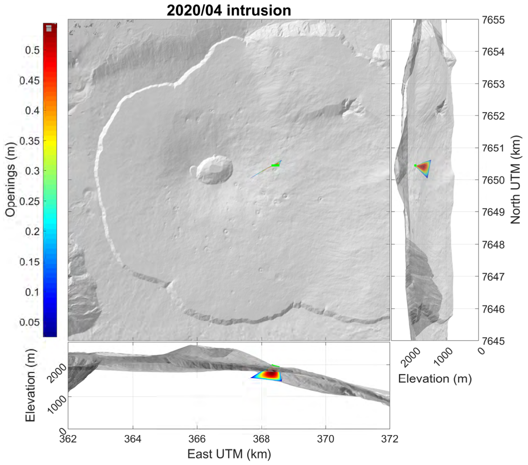

(a) 3D best geometry. Eruptive fissures are indicated by green lines.

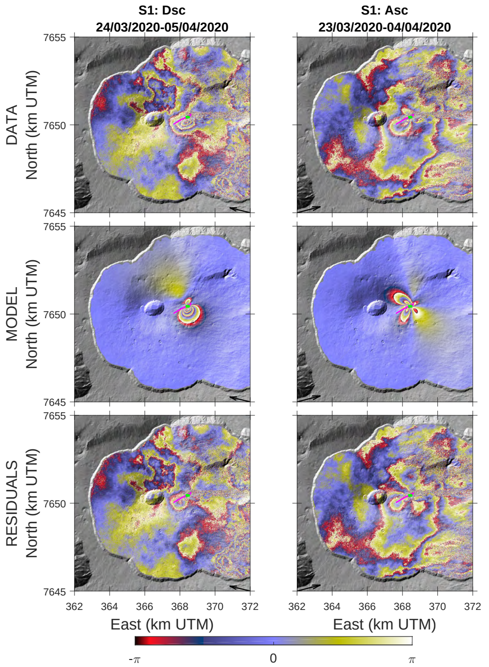

(b) Observed, modeled and residual displacements on wrapped data. Best model contour is in magenta and eruptive fissures are indicated by green lines. Line of sight of satellites acquisition is indicated by arrows.

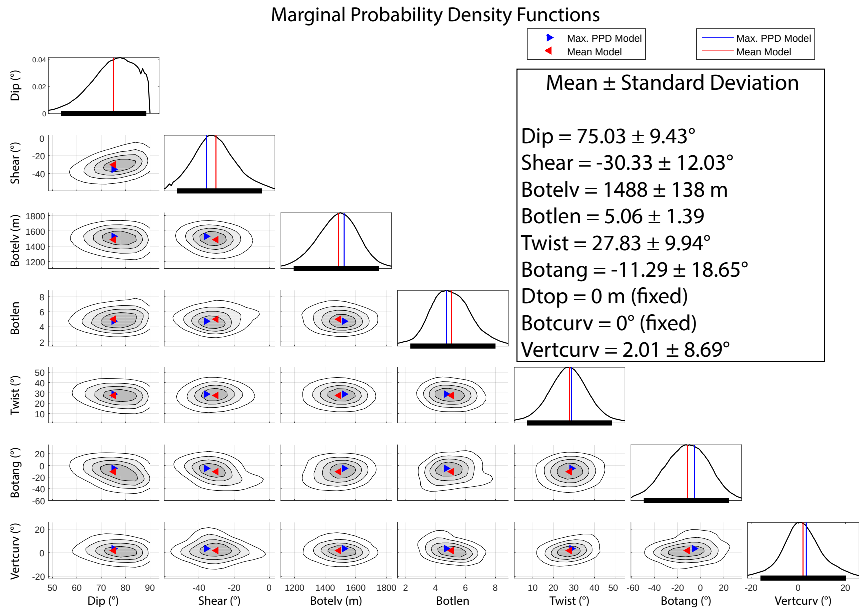

(c) Marginal posterior probability density functions. One-dimensional and two-dimensional functions are given in the diagonal and off-diagonal, respectively. Maximum and mean values are indicated by blue and red triangles, respectively. Black thick lines on one-dimensional functions represent the 95% confidence interval.

Fig. S60: Model for the 2020 April intrusion

September 2020 intrusion

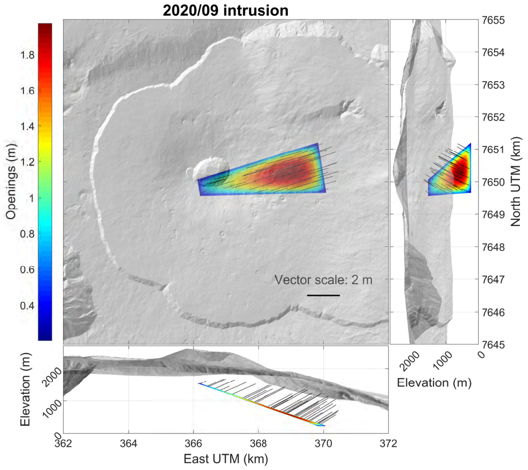

(a) 3D best geometry. Eruptive fissures are indicated by green lines. Opening and shearing of the intrusion are indicated by the black vector magnitudes.

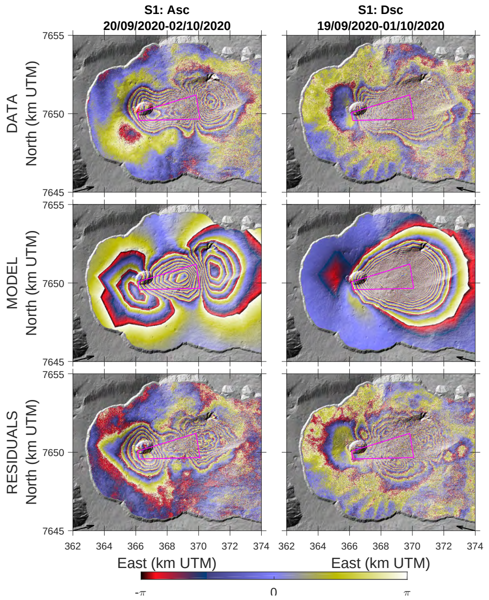

(b) Observed, modeled and residual displacements on wrapped data. Best model contour is in magenta and eruptive fissures are indicated by green lines. Line of sight of satellites acquisition is indicated by arrows.

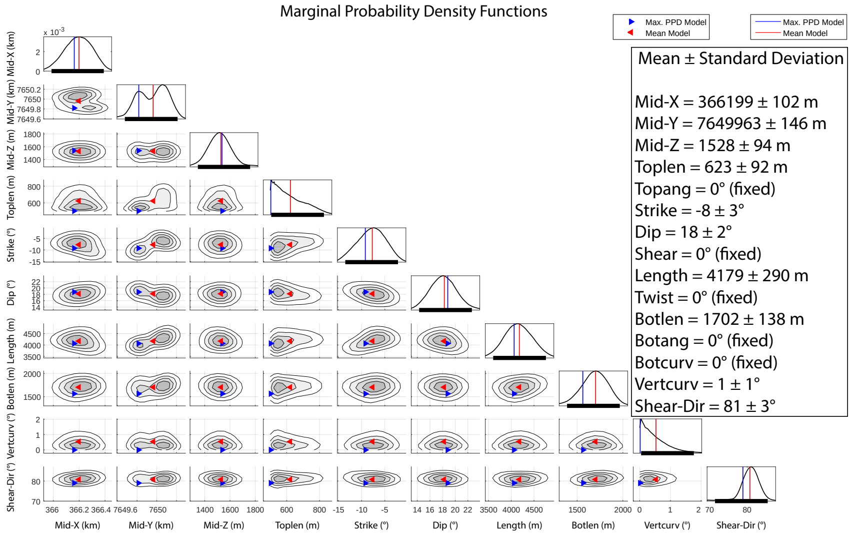

(c) Marginal posterior probability density functions. One-dimensional and two-dimensional functions are given in the diagonal and off-diagonal, respectively. Maximum and mean values are indicated by blue and red triangles, respectively. Black thick lines on one-dimensional functions represent the 95% confidence interval.

Fig. S61: Model for the 2020 September intrusion

December 2020 intrusion

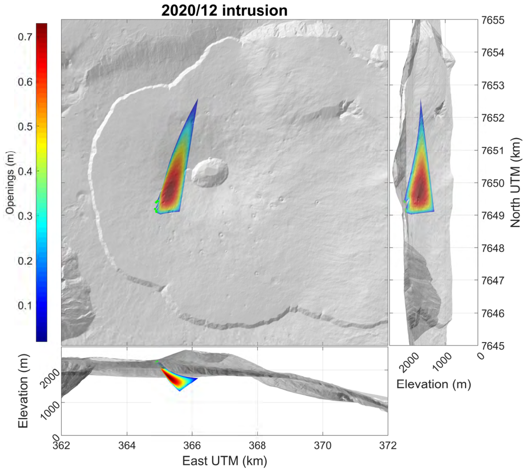

(a) 3D best geometry. Eruptive fissures are indicated by green lines.

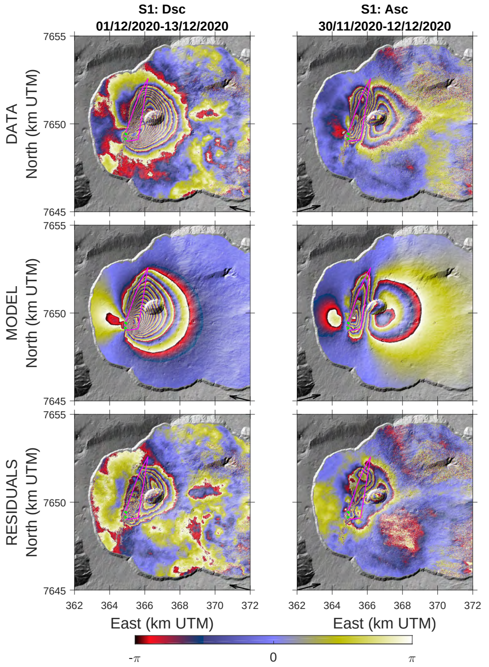

(b) Observed, modeled and residual displacements on wrapped data. Best model contour is in magenta and eruptive fissures are indicated by green lines. Line of sight of satellites acquisition is indicated by arrows.

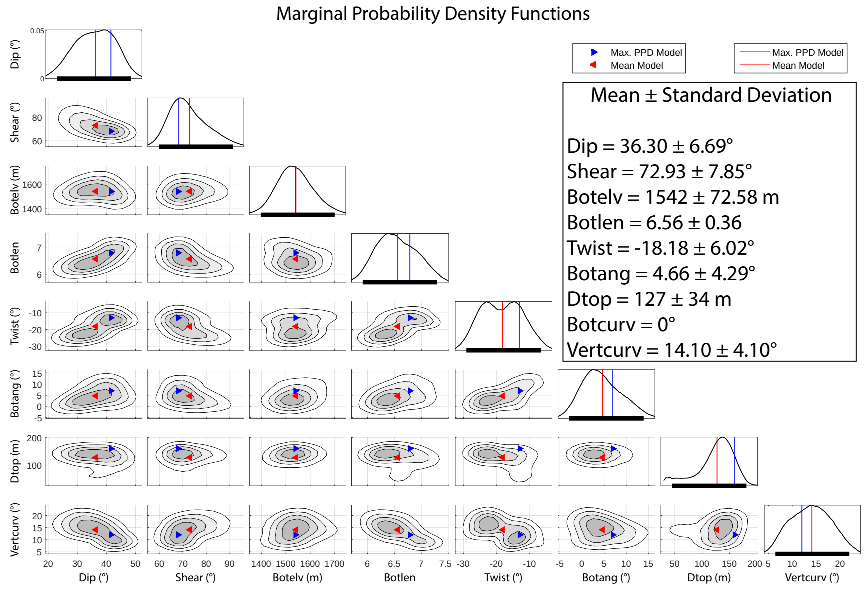

(c) Marginal posterior probability density functions. One-dimensional and two-dimensional functions are given in the diagonal and off-diagonal, respectively. Maximum and mean values are indicated by blue and red triangles, respectively. Black thick lines on one-dimensional functions represent the 95% confidence interval.

Fig. S62: Model for the 2020 December intrusion

3D geometry of the Rift Zones

3D geometry of N60, N120, N210 and N300 Rift Zones

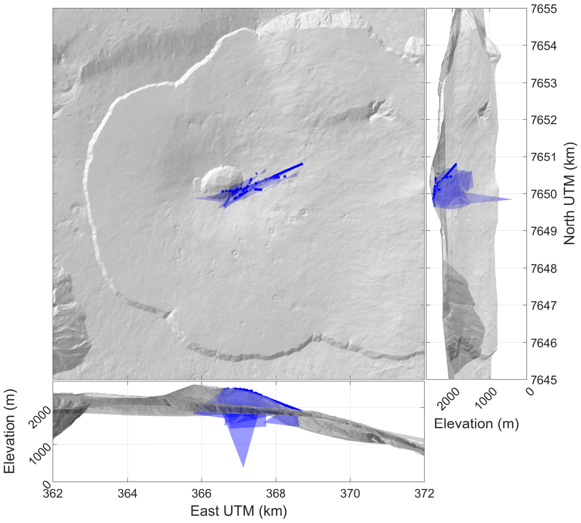

(a) N60° rift zone.

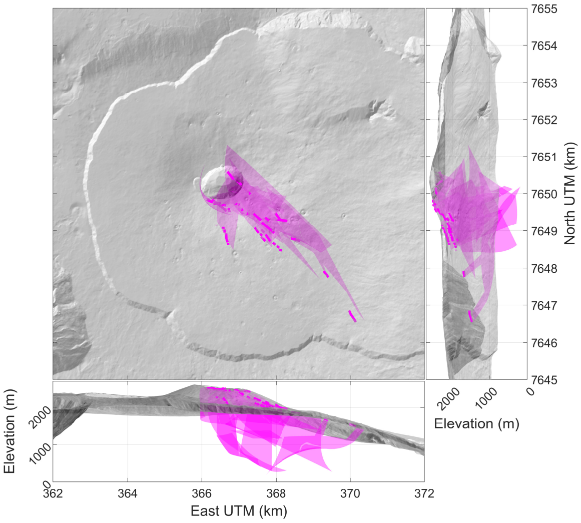

(b) N120° rift zone.

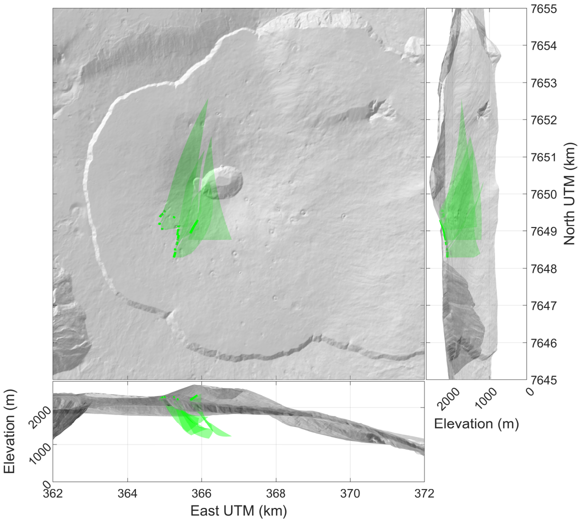

(c) N210° rift zone.

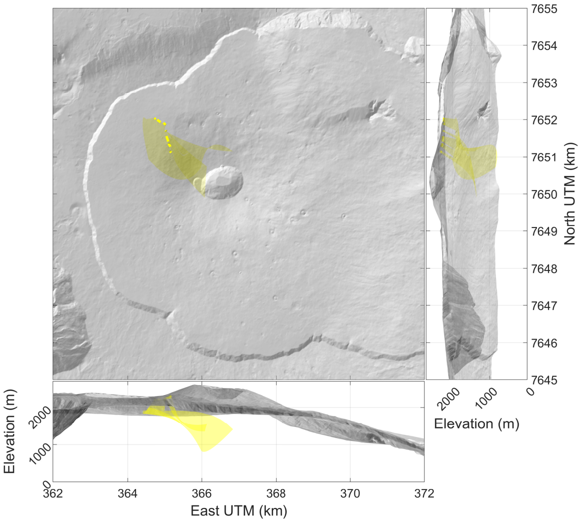

(d) N300° rift zone.

Fig. S63: 3D geometry of rift zones as shown by the best models related to each of them. Eruptive fissures are indicated by segments.

Supplementary References

[1] Fukushima, Y., Cayol, V., Durand, P. & Massonnet, D. Evolution of magma conduits during the 1998-2000 eruptions of Piton de la Fournaise volcano, Réunion Island. *Journal of Geophysical*

Research: Solid Earth **115** (2010).

[2] Fukushima, Y., Cayol, V. & Durand, P. Finding realistic dike models from interferometric synthetic aperture radar data: The February 2000 eruption at Piton de la Fournaise. *Journal of Geophysical Research B: Solid Earth* **110**, 1–15 (2005).

[3] Tridon, M., Cayol, V., Froger, J. L., Augier, A. & Bachèlery, P. Inversion of coeval shear and normal stress of Piton de la Fournaise flank displacement. *Journal of Geophysical Research: Solid Earth* **121**, 7846–7866 (2016).

[4] Cayol, V., Tridon, M., Froger, J., Augier, A. & Bachèlery, P. Inversion of coeval shear and normal stress of Piton de la Fournaise flank displacement. In *IAVCEI 2017 Scientific assembly, Forstering Integrative Studies of Volcanoes*, 180 (2017). URL `http://iavcei2017.org`.

[5] Smittarello, D. *et al.* Magma Propagation at Piton de la Fournaise From Joint Inversion of InSAR and GNSS. *Journal of Geophysical Research: Solid Earth* **124**, 1361–1387 (2019).

[6] Dumont, Q., Cayol, V. & Froger, J.-L. Mitigating bias in inversion of InSAR data resulting from radar viewing geometries. *Geophysical Journal International* **227**, 483–495 (2021).

[7] Tarantola, A. *Inverse Problem Theory*, vol. 120 (Society for Industrial and Applied Mathematics, 2005).

[8] Doin, M. P., Lasserre, C., Peltzer, G., Cavalié, O. & Doubre, C. Corrections of stratified tropospheric delays in SAR interferometry: Validation with global atmospheric models. *Journal of Applied Geophysics* **69**, 35–50 (2009).

[9] Chen, Y. *et al.* Long-term ground displacement observations using InSAR and GNSS at Piton de la Fournaise volcano between 2009 and 2014. *Remote Sensing of Environment* **194**, 230–247 (2017).

[10] Pascal, K., Neuberg, J. & Rivalta, E. On precisely modelling surface deformation due to interacting magma chambers and dykes. *Geophysical Journal International* **196**, 253–278 (2013).

[11] Letourneur, L., Peltier, A., Staudacher, T. & Gudmundsson, A. The effects of rock heterogeneities on dyke paths and asymmetric ground deformation: The example of Piton de la Fournaise (Réunion Island). *Journal of Volcanology and Geothermal Research* **173**, 289–302 (2008).

[12] Duputel, Z. *et al.* The Iquique earthquake sequence of April 2014: Bayesian modeling accounting for prediction uncertainty. *Geophysical Research Letters* **42**, 7949–7957 (2015).

[13] Got, J. L., Peltier, A., Staudacher, T., Kowalski, P. & Boissier, P. Edifice strength and magma transfer modulation at Piton de la Fournaise volcano. *Journal of Geophysical Research: Solid Earth* **118**, 5040–5057 (2013).

[14] Sambridge, M. Geophysical inversion with a neighbourhood algorithm - 1.Searching a parameter space. *Geophysical Journal International* **138**, 479–494 (1999).

[15] Cayol, V. *Analyse elastostatique tridimensionnelle du champ de deformations des edifices volcaniques par elements frontieres mixtes*. Ph.D. thesis, Paris 7 (1996).

[16] Cayol, V. & Cornet, F. H. Three-dimensional modeling of the 1983–1984 eruption at Piton de la Fournaise Volcano, Réunion Island. *Journal of Geophysical Research* **103**, 18025 (1998).

[17] Heap, M. J. *et al.* Towards more realistic values of elastic moduli for volcano modelling. *Journal of Volcanology and Geothermal Research* **390**, 106684 (2020).

[18] Sambridge, M. Exploring multidimensional surfaces without a map. *Inverse Problems* **14**, 427–440 (1998).

[19] Welstead, S. T. *Fractal and wavelet image compression techniques*. TT 40 (The International Society for Optical Engineering, 1999).

[20] Sambridge, M. Geophysical inversion with a neighbourhood algorithm - 2.Appraising the ensemble. *Geophysical Journal International* **138**, 727–746 (1999).
