# Supplementary material for: 22 years of satellite imagery reveal a major destabilization structure at Piton de la Fournaise
Source: Nat Commun. 2022 May 12;13:2649. doi: 10.1038/s41467-022-30109-w (PMC9098438; doi:10.1038/s41467-022-30109-w)
Supplement: Supplementary file 2 — Description of Additional Supplementary Files [file 41467_2022_30109_MOESM2_ESM.pdf]

Description of Additional Supplementary files

File name: Supplementary Movie 1

Description: 3D animated view of the best model geometries of the 28 intrusive events of the main NE-SE and sill intrusion zones. Colorscale indicate the normalized opening value from 0 (blue) to 1 (red). Black lines represent eruptive fissure.

File name: Supplementary Movie 2

Description: 3D animated view of the spread of models of Movie S1 within their 95% confidence interval. Colorscale show the normalized density of points for each models (point densities under 0.2 are not shown for visibility). Black lines represent eruptive fissure.
